# Deciphering the variability in air-sea gas transfer due to sea state and wind history

**DOI:** 10.1093/pnasnexus/pgae389

**Published:** 2024-09-04

**Authors:** Mingxi Yang, David Moffat, Yuanxu Dong, Jean-Raymond Bidlot

**Affiliations:** Plymouth Marine Laboratory, Plymouth PL1 3DH, United Kingdom; Plymouth Marine Laboratory, Plymouth PL1 3DH, United Kingdom; Marine Biogeochemistry Research Division, GEOMAR Helmholtz Centre for Ocean Research Kiel, Kiel 24148, Germany; Institute of Environmental Physics, Heidelberg University, 69120 Heidelberg, Germany; European Centre for Medium-Range Weather Forecasts, Reading RG2 9AX, United Kingdom

## Abstract

Understanding processes driving air-sea gas transfer and being able to model both its mean and variability are critical for studies of climate and carbon cycle. The air-sea gas transfer velocity (*K*_660_) is almost universally parameterized as a function of wind speed in large scale models—an oversimplification that buries the mechanisms controlling *K*_660_ and neglects much natural variability. Sea state has long been speculated to affect gas transfer, but consistent relationships from in situ observations have been elusive. Here, applying a machine learning technique to an updated compilation of shipboard direct observations of the CO_2_ transfer velocity (*K*_CO2,660_), we show that the inclusion of significant wave height improves the model simulation of *K*_CO2,660_, while parameters such as wave age, wave steepness, and swell-wind directional difference have little influence on *K*_CO2,660_. Wind history is found to be important, as in high seas *K*_CO2,660_ during periods of falling winds exceed periods of rising winds by ∼20% in the mean. This hysteresis in *K*_CO2,660_ is consistent with the development of waves and increase in whitecap coverage as the seas mature. A similar hysteresis is absent from the transfer of a more soluble gas, confirming that the sea state dependence in *K*_CO2,660_ is primarily due to bubble-mediated gas transfer upon wave breaking. We propose a new parameterization of *K*_CO2,660_ as a function of wind stress and significant wave height, which resemble observed *K*_CO2,660_ both in the mean and on short timescales.

**Significance Statement**

The ocean is a key sink of CO_2_, and the instantaneous rate of ocean CO_2_ uptake is proportional to the air-sea gas transfer velocity. Transfer velocity is almost universally parameterized as a function of wind speed only in global models, which is a reasonable approximation in the mean but neglects physical mechanisms and so variability. Here, combining the largest observational dataset to date we demonstrate that there are substantial variations in CO_2_ transfer at short timescales due to sea state and wind history. We propose a new parameterization of the gas transfer velocity based on wind and waves data to more accurately predict air-sea CO_2_ flux.

## Introduction

The ocean has absorbed ∼30% of the atmospheric CO_2_ emitted from human activities since the industrial revolution (e.g. (1)). Reducing the uncertainties in air-sea CO_2_ flux and improving our understanding in the processes controlling it are thus critical for monitoring ongoing change and projecting the future climate. Air-sea flux of a gas such as CO_2_ can be measured directly by the eddy covariance (EC) technique (e.g. (2)), but such monitoring has not been implemented on a large spatial and temporal scale. Instead, flux is generally estimated from a parameterization of the gas transfer velocity, *K*_660_, and the gas concentration difference between air and water near the interface, ΔC: Flux = *K*_660_ (Sc/660)^−0.5^ ΔC. Here, Sc is the Schmidt number of the gas in water. Thus, any error in *K*_660_ is directly propagated to the flux estimate and is in fact the dominant source of flux uncertainty (e.g. (3)).

Wind blowing over the ocean provides the predominant, but indirect forcing for *K*_660_. This is because for sparingly soluble gases like CO_2_ and dimethyl sulfide (DMS), their air-sea exchange is ultimately controlled by complex waterside processes (4). *K*_660_ in global models is almost universally parameterized as a simple function of wind speed (*U*_10*n*_), oversimplifying the underlying physical mechanisms. Such *K*_660_ parameterizations become especially unsatisfying when gases of different solubility (e.g. CO_2_ and DMS) require different wind speed fits (e.g. (5)).

Mechanistic models usually partition *K*_660_ into two parallel processes (*K*_660_ = *k_d_* + *k_b_*): (ⅰ) diffusive transfer through an unbroken surface (*k_d_*) due to viscous wind stress and surface renewal, which is relatively more important at lower wind speeds and (ⅱ) bubble-mediated transfer (*k_b_*) upon wave breaking, which becomes increasingly important at higher wind speeds. *k_d_* for different waterside-controlled gases should be identical when normalized to a reference Schmidt number, and the transfer velocity of the more soluble DMS (*K*_DMS,660_) is often thought to approximate *k_d_* (e.g. (6, 7)). In contrast, *k_b_* is relatively more important for the less soluble CO_2_ than for DMS (5–9). Reichl and Deike (10) estimated that *k_b_* contributes to 30% of CO_2_ exchange globally by combining a mechanistic bubble-breaking model (11) with significant wave height data from the Wavewatch III model. A survey of literature, though, shows an order-of-magnitude discrepancy in the estimation of *k_b_* (11–17), illustrating our poor understanding in bubble-mediated transfer.

Direct air-sea CO_2_ flux measurements on ships by the EC method have improved dramatically in quality over the last 1.5 decades. Yang et al. (2) synthesized and reevaluated CO_2_ flux observations from 11 cruises, primarily from the North Atlantic and Southern Ocean but also including the Tropical Indian Ocean and the Arctic. Dividing CO_2_ flux by the concurrently measured air-sea CO_2_ concentration difference and applying a Schmidt number normalization yields *K*_CO2,660_. This foundational dataset (over 2,000 h) shows that the SD in the mean *K*_CO2,660_ vs. wind speed relationship is about 20% (range >50%), with typically higher *K*_CO2,660_ at a given wind speed in regions of larger waves such as the North Atlantic.

The physical interaction between wind and waves is complex and nonlinear. Simplistically, wind blowing over the ocean surface provides the momentum to produce windsea, which after some separation in time and space becomes swell. Breaking waves generate bubbles, which are manifested as whitecaps on the ocean surface. Sea state has long been speculated to affect *K*_660_ but deciphering consistent relationships from in situ data has been difficult. This is likely due to a combination of factors:

- *K*_660_ over the open ocean is challenging to measure. Each research cruise typically returns 100–200 valid data points (if measured by EC) or a few data points (if inferred from tracers, e.g. (18)), which do not cover the full range of sea states.
- There is substantial scatter in the *K*_660_ observations. Until recently, the partitioning in scatter between natural variability and measurement uncertainty was unclear. Dong et al. (19) comprehensively determined the random uncertainty in EC *K*_CO2,660_ data to be about 30% under typical conditions for hourly measurements (generally decreasing with increasing wind speed and flux magnitude).
- Wind and wave parameters tend to correlate with each other, and which wave parameters are of importance toward *K*_660_ is not well known.

Some sea state dependencies in *K*_660_ have been proposed, but with little consensus. Zhao et al. (20) proposed the wave Reynolds number (*R*_Hw_ = u_*_ × *H_s_*/*v_w_*) that includes both wind and wave information as a parameter to describe *K*_660_. Here, u_*_, *H_s_*, and *v_w_* are the friction velocity (related to surface wind stress), significant wave height, and water viscosity, respectively. This theoretical framework implies that *K*_660_ should be greater in more developed seas than in developing seas. Brumer et al. (21) showed that the use of *R*_Hw_ collapses different field observations of *K*_660_ better than wind speed. Blomquist et al. (5) and Fairall et al. (17) adopted this $R_{\mathrm{Hw}}^{0.9}$ scaling for *k_b_*. Deike and Melville (11) developed a mechanistic model of *k_b_*, which can either take on a spectral form or be related to bulk wave parameters: proportional to $u_{*}^{5/3}$$H_{s}^{2/3}$. Using the spectral model, *k_b_* was modeled to be greater during the developing phase of a storm in the Southern Ocean (22), in contrast to the *R*_Hw_ formulation. Zavarsky and Marandino (23) further suggested a dependence in *K*_DMS,660_ on the swell-wind directional difference, which certain directions causing more “shielding” of wind from swell, resulting in suppressed transfer.

In this work, we investigate the sea state dependencies in *K*_660_ from multiple datasets of *K*_CO2,660_, *K*_DMS,660_, waves, and whitecap coverage. We first quantify the variability in the *K*_CO2,660_ observations that cannot be explained by wind speed and by measurement noise. We then use a machine learning (ML) method to “agnostically” investigate relationships between *K*_CO2,660_ and various wave parameters. The ML approach elucidates the potential key controlling parameters for *K*_CO2,660_. Further in depth analysis of these parameters as well as comparison between *K*_CO2,660_ and *K*_DMS,660_ enable us to tease out the driver for the sea state dependence in *K*_CO2,660_. Finally, we propose and evaluate a new parameterization of *K*_CO2,660_ based on wind and bulk wave data.

## Variability in the hourly *K*_CO2,660_ observations

**Fig. 1.**
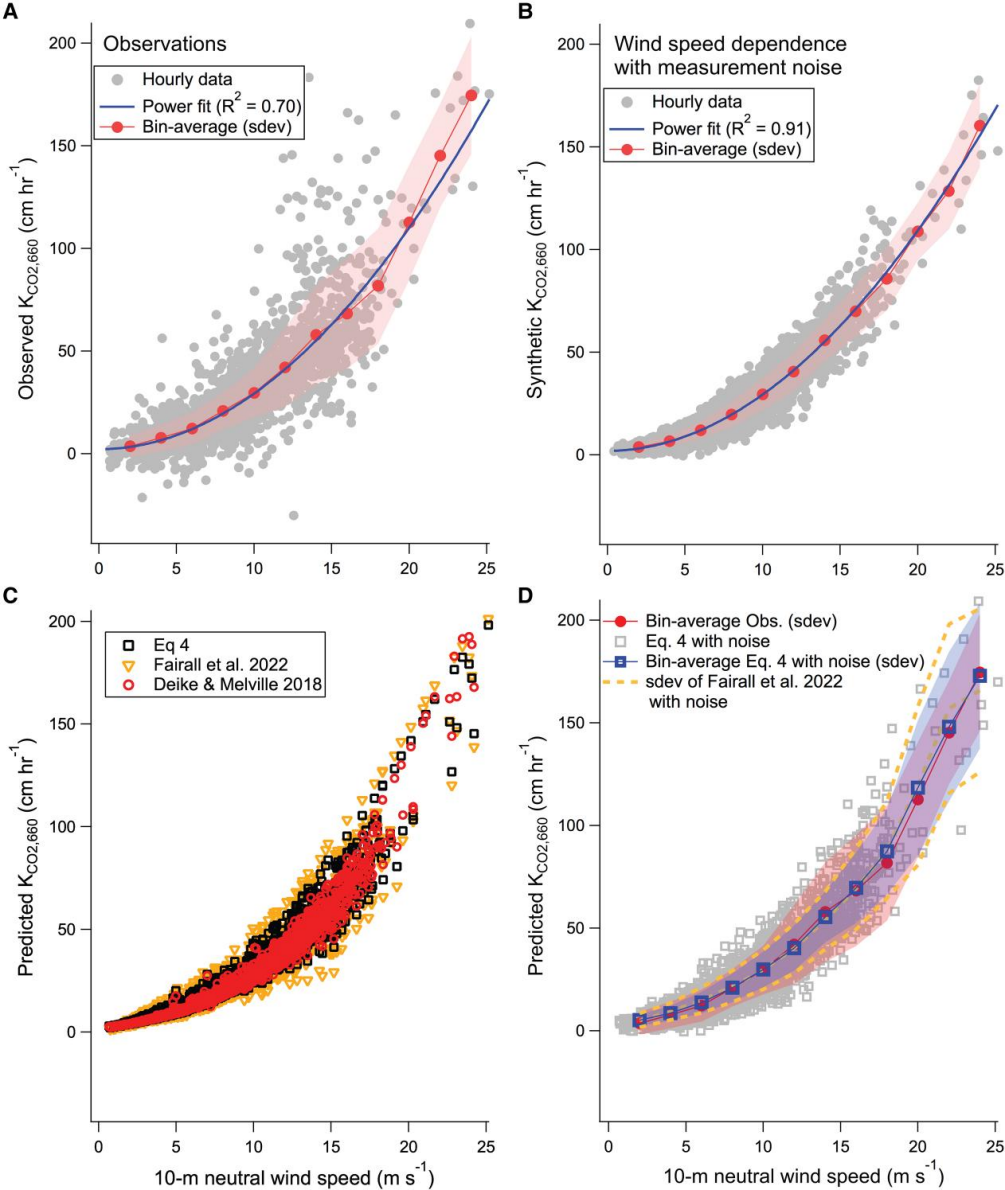
A) Observed *K*_CO2,660_ vs. wind speed (hourly) from the entire dataset; the thick solid line shows the mean wind speed dependence (power fit) and the thin line with markers show bin-average with SD. B) Synthetic *K*_CO2,660_ (hourly) based on the wind speed dependence, accounting for random measurement noise. C) Predicted *K*_CO2,660_ (hourly) from Eq. 4, Deike and Melville (11), and Fairall et al. (17). D) Predicted *K*_CO2,660_ (hourly) from Eq. 4 with random measurement noise, which approaches observations in the mean and in variability. Variability predicted by Fairall et al. (17) appears to be too large at high wind speeds relative to observations.

In this paper, we have added ∼600 hours of *K*_CO2,660_ data from four recent cruises in the Southern Ocean (24, 25) to the foundational dataset of *K*_CO2,660_ observations (∼2000 hours) from 11 cruises by Yang et al. (2), forming the largest *K*_CO2,660_ dataset to date. Please refer to these two papers for the exact location/time of the cruises. A simple power fit as a function of *U*_10*n*_ to all the *K*_CO2,660_ observations returns a *R*^2^ of 0.70 (Fig. 1A). How much of the residual variance is due to noise vs. natural variability? To answer this, we generate a set of synthetic *K*_CO2,660_ data based on the *U*_10*n*_ fit with a Gaussian random noise that averages 30% (19). This synthetic data shows that the maximum possible *R*^2^ between *K*_CO2,660_ and a dependent variable within this dataset is 0.91 (Fig. 1B). Wind is indeed the predominant driver for gas transfer, but there remains substantial variability not explained by *U*_10*n*_ (variance gap of 0.91–0.70 = 0.21). This is particularly true at high wind speeds (e.g. 15 to 20 m s^−1^), where variance in the synthetic *K*_CO2,660_ dataset is only about a quarter of the variance in the observations. Clearly, confining our attention to averages of *K*_CO2,660_ in wind speed bins, as is the norm in past decades of research, neglects much natural variability caused by other factors such as sea state.

## Deciphering sea state dependence in *K*_CO2,660_ using ML

To investigate controlling factors in gas transfer, we developed a ML model (random forest, (26)) for the prediction of hourly *K*_CO2,660_ based only on the foundational dataset of Yang et al. (2). Model input parameters, consisting primarily of wind (in situ) and bulk wave (ECMWF) parameters, are detailed in the Supplementary Material. Wave parameters from ECMWF were computed from the full modeled wave spectrum, as well as separately for windsea and swell components. In the spectral wave model, the area of the spectrum where the wind input is actively transferring momentum into waves is defined as windsea. The remaining part of the wave spectrum is defined as swell.

**Fig. 2.**
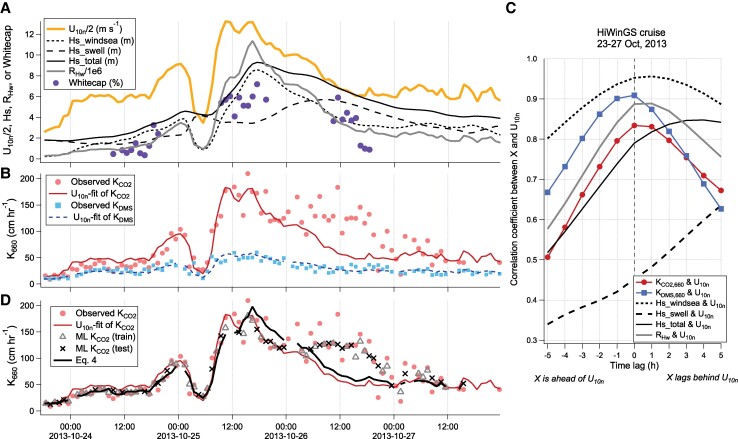
An example of the impact of waves on gas transfer during the “St. Jude” storm of the HiWinGS cruise. A) Wind speed peaked in excess of 25 m s^−1^ on 25 October, while significant wave heights (total and swell) and whitecap fraction remained elevated for hours longer. B) A simple *U*_10*n*_ dependent fit to the HiWinGS *K*_CO2,660_ observations tends to overestimate during periods of rising winds and developing seas (e.g. 24 October), and substantially underestimate during periods of falling winds and more developed seas (e.g. 26 October). C) Lag correlation analysis of the period during the St. Jude storm shows that *K*_CO2,660_ is lagged relative to *U*_10*n*_, and this hysteresis is most similar to that of the wave Reynolds number (here the viscosity of water at 20°C was used to compute *R*_Hw_). A similar sea state dependent hysteresis is not observed in *K*_DMS,660_, which is well described by a *U*_10*n*_ dependence throughout the storm. D) Predictions from the ML model and Eq. 4 of *K*_CO2,660_. Overall, the ML model generalizes the mean trend of the observations well. A small degree of over-fitting is apparent as the ML model often tries to match the individual “wiggles” in the training data, which are partially due to measurement noise. Equation 4 from this work outperforms a simple wind speed fit but does not capture as much natural variability as the ML model following this extreme storm event (e.g. 26 October).

The random forest model was developed with 200 trees, a leaf size of one, and the trees were trained to minimize the means squared error. From the cruise data collected, periods of incomplete data were removed, and standard scaling was performed to normalize the rest of the data to zero mean and unit variance. The data were then randomly split into training (65%) and testing (35%) datasets (see Fig. 2D as an example). The 65/35% split was applied to all the cruises within the foundational dataset to ensure suitable temporal and geographical representations within both the training and testing datasets. We reserve the new Southern Ocean data from Dong et al. (24, 25) as an independent dataset for further model validation.

Within the foundational dataset, the ML model captures a *R*^2^ of 0.97 in the training data and 0.81 for the testing data (Figs. S1 and S2). The *R*^2^ for training exceeds the theoretical maximum *R*^2^ of 0.91 (Fig. 1B). This reflects a small degree of “over-fitting” by the ML model, which does not appear to affect how the ML predicts nontraining data (Fig. 2D). The *R*^2^ for testing surpasses the *R*^2^ of a simple wind speed fit by 0.11 but is lower than the maximum possible *R*^2^ of 0.91, likely in part because we have neglected in the ML model some other processes that can also affect *K*_CO2,660_, such as surfactants (e.g. (27, 28)) and convection (29). A separate random forest ML model run with wind input only yields an *R*^2^ of 0.75 for the testing data. This shows that (ⅰ) inclusion of wave parameters improves the ML model of *K*_CO2,660_ (by *R*^2^ of about 0.81 − 0.75 = 0.06), and (ⅱ) ML is able to identify wind-dependent patterns in *K*_CO2,660_ (e.g. wind history) that a simple wind speed fit neglects (by *R*^2^ of about 0.75 − 0.70 = 0.05).

When we validate the ML model with wave parameters against the new *K*_CO2,660_ observations that were not used for the training (24, 25), the model slightly over predicts (*R*^2^ of 0.66, Fig. S2), perhaps due to the aforementioned other controlling factors for gas transfer that have not been considered. The Pearson correlations between observations and predictions remains high though (*r*^2^ = 0.74), implying that the ML model does capture most of the variations in *K*_CO2,660_ in the validation dataset. The random forest ML algorithm is publicly available at GITHUB (https://github.com/djmoffat/air-sea-gas-prediction). We also explored the use of a small Artificial Neural Network, but this underperformed compared with the random forest model, likely due to the still relatively limited observational dataset.

To further assess which input predictor has the largest impact on the predictions from the ML model, we turn to SHAP (SHapley Additive exPlanations; (30)) analysis. The SHAP value (Fig. S3) shows that the most important parameters in predicting *K*_CO2,660_ are *U*_10*n*_ and significant wave heights (windsea, swell, and total), while parameters such as wave age, swell-wind direction difference, swell period, and wave steepness are generally not important.

## Example of sea state dependence from the HiWinGS cruise

Clear sea state dependence in *K*_CO2,660_ is apparent in the data from the HiWinGS cruise (2013 in the North Atlantic; originally published by (5) and recomputed in the case of CO_2_ by (2)), during which the ship stationed through multiple exceptionally large storms (Fig. 2). The highest wind speed (>25 m s^−1^) was observed on 25th October 2013 in what eventually was named the “St Jude” storm upon landfall in the United Kingdom. *K*_CO2,660_ was expectedly very high at the peak in wind speed, but interestingly the high *K*_CO2,660_ values persisted for many hours following the wind peak. A simple *U*_10*n*_ fit to all the HiWinGS *K*_CO2,660_ observations clearly underestimates the observed *K*_CO2,660_ for most of 26th October, when the seas declined following the peak of the storm. At the same time, the *U*_10*n*_ fit overestimates *K*_CO2,660_ for most of 24th October, when the seas were building up. In contrast, we do not see any difference in *K*_DMS,660_ (a proxy of diffusive transfer) between rising and falling winds/seas during the HiWinGS cruise.

Fairly long periods of continuous measurements and the large range in wind conditions during HiWinGS allow us to assess the time lag between gas transfer and its drivers (Fig. 2C). Consistent with our understanding of the development of the seas, windsea lagged behind wind by 1–2 h, while swell lagged behind wind by much longer. There is a clear asymmetry in the *K*_CO2,660_:*U*_10*n*_ lag correlation. This hysteresis implies that *K*_CO2,660_ has some lag relative to *U*_10*n*_, similar to the behavior of the wave Reynolds number computed from the total significant wave height. In contrast to *K*_CO2,660_, *K*_DMS,660_ correlates symmetrically with respect to *U*_10*n*_ and its peak correlation with *U*_10*n*_ is higher than that of *K*_CO2,660_, suggesting minimal sea state effect.

**Fig. 3.**
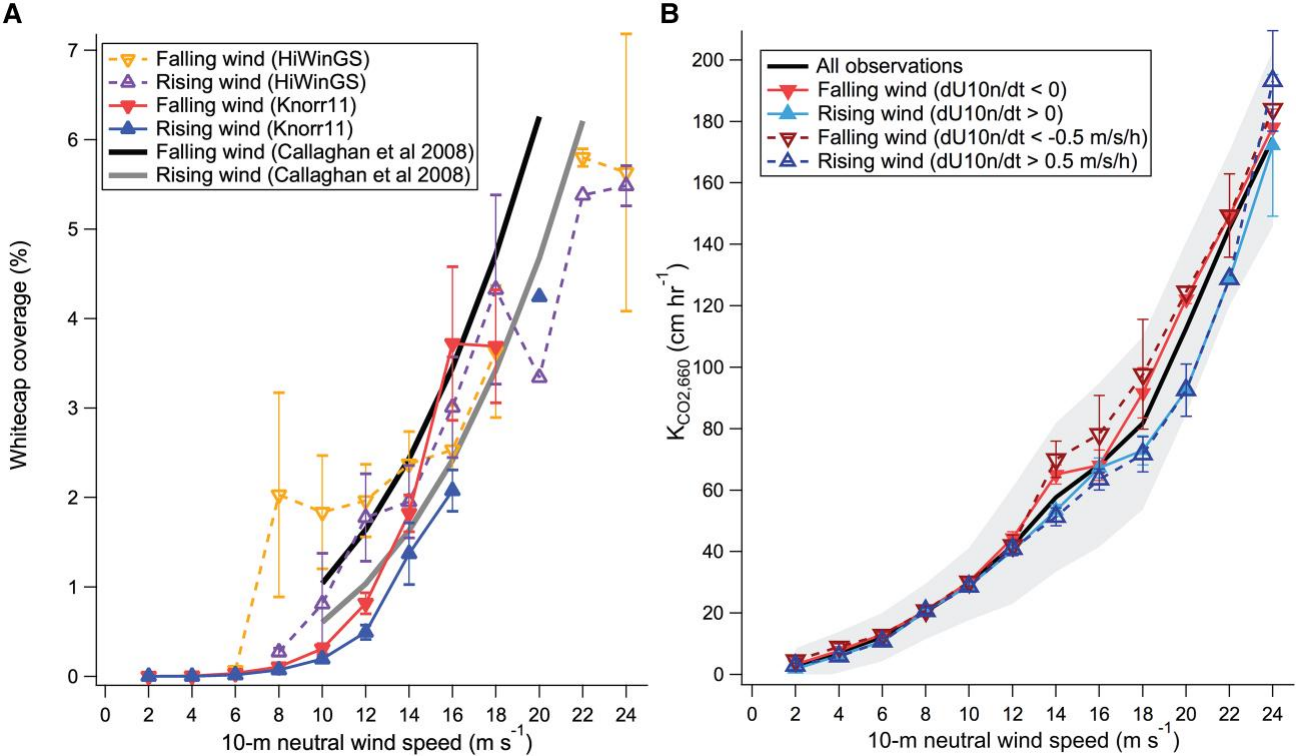
Wind history dependence in whitecap coverage from three different cruises (A) and in CO_2_ transfer from 15 cruises (B). At high wind speeds, whitecap coverage tends to be greater during falling winds than during rising winds (by about 50% for the Knorr11 cruise and (33)), while *K*_CO2,660_ is on average about 20% greater during falling winds than during rising winds. The error bars on rise/fall categories indicate SE, while the gray shading on the overall bin-average in B (black line) indicates SD.

The measured whitecap coverage (*W_f_*) was nearly three times higher for most of 26th October than on 24th October, despite very similar wind speeds at around 12 m s^−1^. Measurements of bubbles during the HiWinGS cruise also showed higher bubble void fractions during falling winds than rising winds (31). On aggregate, the higher whitecap coverage during falling winds and more developed seas agrees well with previous observations from the Knorr11 cruise (32) and with Callaghan et al. (33), as shown in Fig. 3A and Table S1. Here rising (falling) wind is defined as dU_10*n*_/dt of the preceding three hours (including hour of interest) being >0 (<0), as in Hanson and Phillips (34) and Callaghan et al. (33). An analogous relationship with wind history was observed for sea spray flux from a coastal site (35). The similar behaviors in *K*_CO2,660_ and *W_f_* toward wind history and the lack of wind history dependence in *K*_DMS,660_ suggest that the hysteresis in *K*_CO2,660_ is mostly due to bubble-mediated transfer upon wave breaking.

## Mean sea state and wind history dependencies in *K*_CO2,660_

Conditions during the HiWinGS cruise were rather extreme but a similar sea state dependence is apparent at lower wind speeds. We now turn our attention to the entire *K*_CO2,660_ dataset. As with *W_f_*, for each hour we compute wind history as dU_10*n*_/dt over the preceding three hours. The *K*_CO2,660_ data are then stratified into categories of “rising wind” (dU_10*n*_/dt >0, *N* = 1074; or >0.5 m s^−1^ h^−1^, *N* = 465) and “falling wind” (dU_10*n*_/dt <0, *N* = 1197; or <−0.5 m s^−1^ h^−1^, *N* = 487). At the same wind speed, on average *K*_CO2,660_ during falling winds exceeds *K*_CO2,660_ during rising winds by on the order of 20%, with the steeper threshold of |0.5 m s^−1^ h^−1^| usually leading to a greater enhancement than a threshold of 0 (Fig. 3B; Table S1). The enhancement appears to be most pronounced at wind speeds above 12 m s^−1^, though above 20 m s^−1^ the number of observations becomes very limited.

Periods of falling wind are typically associated with more developed seas (and relatively high wave age), while periods of increasing wind are typically associated with less developed seas (and relatively low wave age). The ML model does not find wave age (and inverse wave age) to be an important controller factor of *K*_CO2,660_. This may be because the parameter wave age (phase speed of wave divided by wind speed) is highly dependent on wind speed, with high wind speed conditions usually corresponding to less developed seas (e.g. (36)). Including it thus provides limited additional information over what is already provided by wind speed. In comparison, wind history is not very dependent on wind speed on the whole.

So what causes the wind history dependence in the *K*_CO2,660_ observations? Here, we separate the hourly *K*_CO2,660_ data in 1 m s^−1^ wind speed bins (to remove the *U*_10*n*_ dependence) and then compute the correlations between *K*_CO2,660_ and significant wave heights as well as wind history within the bins (Table S2). The key findings from this analysis are:

- Correlations between *K*_CO2,660_ and significant wave heights are almost always positive, and the correlations are usually statistically significant (95% confidence) at *U*_10n_ above 7 m s^−1^, consistent with substantial bubble-mediated transfer for CO_2_.
- Total significant wave height correlates most strongly with *K*_CO2,660_, with both windsea and swell components contributing toward the correlation, qualitatively consistent with ML SHAP analysis (Fig. S3).
- Correlations between *K*_CO2,660_ and wind history are generally negative (consistent with Fig. 3B), but the correlations are weaker than with significant wave heights.

The above findings support the approach of parametrizing *k_b_* (and not *K*_660_ directly) as a function of total *H_s_*, as in Blomquist et al. (5), Deike and Melville (11), and Fairall et al. (17). The wind history dependence in *K*_CO2,660_ appears to reflect the *H_s_* dependence to a large extent—that *K*_CO2,660_ is higher during falling winds than rising winds at the same wind speed is partly because waves (and whitecap coverage) tend to be greater during falling winds than rising winds (Fig. S4). This understanding forms the foundation for our new parameterization of *K*_CO2,660_ based on bulk wind and sea state parameters.

## Building a new wind-wave parameterization of *K*_CO2,660_

Deike and Melville (11) proposed a parameterization of *K*_660_ based on bulk wind and wave parameters:


(1) $$K_{660}=k_{d}+k_{b}=Au_{*}+B{u_{*}}^{5/3}(gH_{s}{)}^{2/3}$$

Here, we have for simplicity removed from their formula the dependence on Ostwald solubility (which for CO_2_ is close to unity). *A* and *B* are tunable coefficients, while *g* is gravity. Zhou et al. (37) adopted the same formulation as Eq. 1 but with different *A* and *B* coefficients.

Fairall et al. (17), following Blomquist et al. (5), proposed an alternative representation of *K*_660_ based on the COARE gas transfer model, which (ignoring buoyance effects at low wind speeds) can be simply represented as:


(2) $$K_{660}=k_{d}+k_{b}=Au_{v*}+BW_{f}\;=Au_{v*}+B(R_{\mathrm{Hw}}{)}^{0.9}.$$

Here, *u_v∗_* is the viscous part of *u*_∗_ — a parameter described by Mueller and Veron (38) that cannot be readily measured in the field. *W_f_* is the whitecap coverage, which is parameterized as a function of the wave Reynolds number (*R*_Hw_ = *u_∗_ H_s_*/*v_w_* at a mean HiWinGS temperature of 10°C) according to the works from Brumer et al. (39). *A* and *B* again are tunable coefficients, which are constrained by limited observations of both *K*_CO2,660_ and *K*_DMS,660_. Compared with Deike and Melville (11), *k_b_* in Fairall et al. (17) is ∼70% greater while *k_d_* is 30%∼smaller.

In both equations above, *k_b_* depends on wind stress as well as wave heights. Yang et al. (2) showed that at low to moderate wind speeds, *K*_CO2,660_ has an essentially linear dependence on u_*_, consistent with the idea that diffusive transfer is driven by wind stress. Here, we opt to parameterize *k_d_* as a function of u_*_ instead of u_v__*_, since the former is more readily measurable in the field and already available in large datasets (e.g. ECMWF reanalysis). Since *W_f_* appears to have a near linear dependence on *R*_Hw_ (39), for our parameterization we start with the basic form below:


(3) $$K_{\mathrm{CO}2,660}=k_{d}+k_{b}=Au_{*}+BW_{f}\;=Au_{*}+Bu_{*}H_{s}.$$

Here, we have neglected the dependence on *v_w_*, which is convenient for simplifying units in *R*_Hw_, but does not help to constrain variability in *K*_CO2,660_ (2). The bulk u_*_ is used, which is derived from in situ meteorological and underway seawater observations using the COARE3.5 model (with a wind speed dependent Charnock relation). We note that during windsea-dominated conditions, *H_s_* approximately scales with $u_{*}^{2}$. Then, the *k_b_* term in Eq. 3 (as well as in Eq. 1) scales with $u_{*}^{3}$, which is in line with historical *W_f_* observations and the concept that *W_f_* is strongly related to the energy flux from the wind (40). Compared with the Eq. 1, the *k_b_* term in Eq. 3 is more weighted toward wave height and less toward wind stress in the presence of swell.

**Fig. 4.**
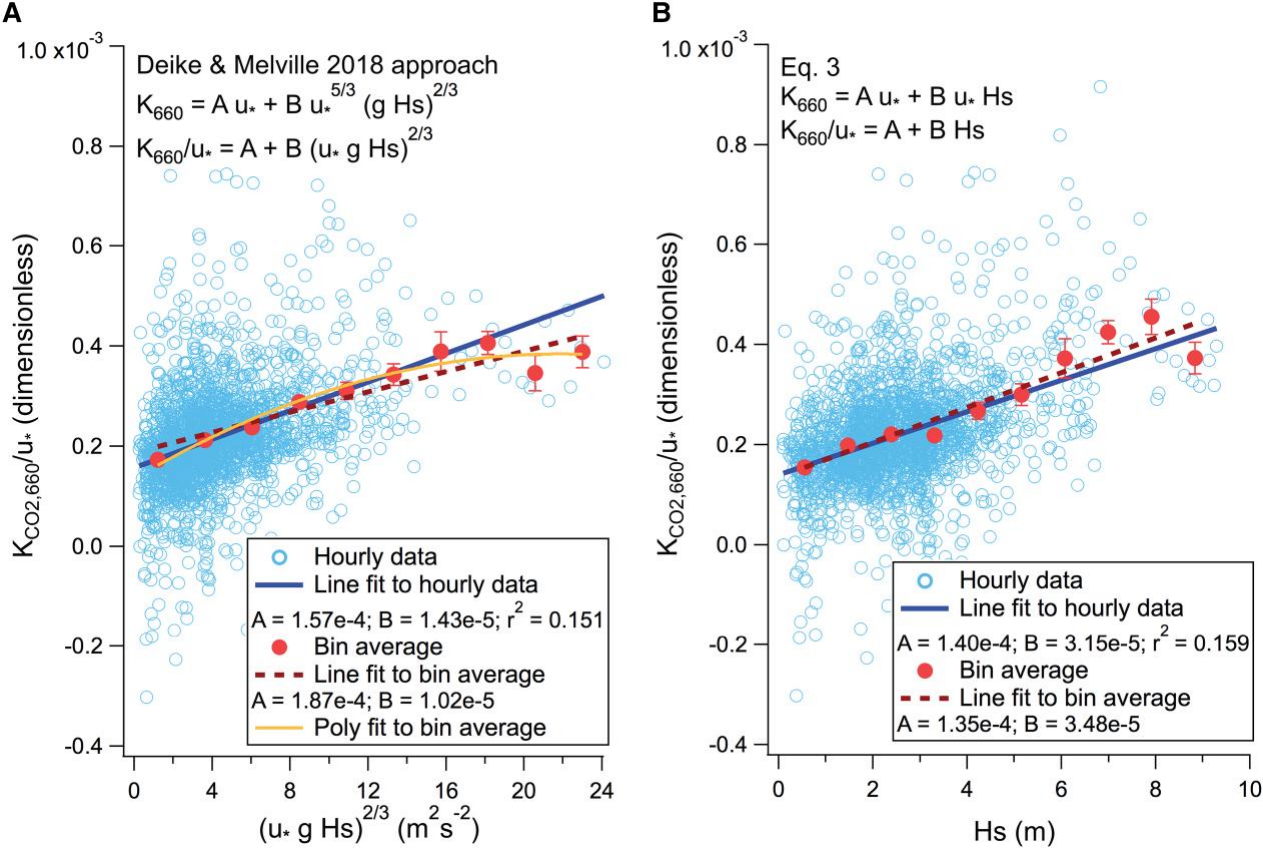
A) *K*_CO2,660_/u_*_ vs. (u_*_ g H_s_)^2/3^, which clearly shows a nonlinear relationship. Here, the intercept and the slope correspond to tuning coefficients A for diffusive transfer (dimensionless) and B for bubble-mediated transfer (*s*^2^ m^−2^); B) *K*_CO2,660_/u_*_ vs. *H_s_*, which has a more linear relationship and higher *r*^2^. A polynomial fit to (B) looks nearly identical to the linear fit. Here, the intercept and the slope correspond to tuning coefficients A for diffusive transfer (dimensionless) and B for bubble-mediated transfer (m^−1^).

Since *K*_CO2,660_ in Eqs. 1 and 3 are both dependent on u_*_, we can divide observed *K*_CO2,660_ by u_*_ to evaluate the functional form for *k_b_* and assess the coefficients *A* and *B*. For this analysis, we neglect periods when u_*_ < 0.1 m s^−1^, as the relative measurement uncertainty is substantially larger and other effects such as buoyancy may become important under these calm conditions. For the Deike and Melville (11) approach, *K*_CO2,660_/u_*_ appears to have a nonlinear relationship with (u_*_g H_s_)^2/3^ (Fig. 4A). In contrast, *K*_CO2,660_/u_*_ and *H_s_* has essentially a linear relationship (Fig. 4B). From this it seems that the functional form of Eq. 3 is reasonable and seems more consistent with *K*_CO2,660_ observations than Eq. 1 (11) over the full range of sea state.

If we fit the observed *K*_CO2,660_ as a function of u_*_ and *H_s_* simultaneously following the functional form of Eq. 3, we arrive at the following parameterizations of *K*_CO2,660_ (in units of cm h^−1^):


(4) $$K_{\mathrm{CO}2,660}=360,000(1.52e-4u_{*}+2.90e-5u_{*}H_{s}).$$

Interestingly, fitting *K*_CO2,660_ with *H_s__*_windsea_ instead of total *H_s_* does not improve the fit, similar to findings from Blomquist et al. (5) and Brumer et al. (21). Swell appears to be important for CO_2_ transfer, as also implied from the SHAP analysis (Fig. S3) as well as from correlations between *K*_CO2,660_ and the different *H_s_* components (Table S2). It is worth cautioning though that the spectral separation between windsea and swell in the ECMWF wave model is fairly simplistic. In cases of rapidly changing wind fields, the part of the spectrum that is defined as swell might still contain steep breaking waves that contribute to gas exchange.

Tuned to overlapping EC datasets, recent parameterizations based on wind and waves all predict similar *K*_CO2,660_ in the mean. However, these parameterizations differ in the partitioning between *k_d_* and *k_b_*. At a wind speed of 15 m s^−1^, *k_b_* accounts for on average 39% of total *K*_CO2,660_ in Deike and Melville (11), 66% in Fairall et al. (17), 43% in Zhou et al. (37), and 42% according to Eq. 4. At this wind speed, the relative difference in observed *K*_CO2,660_ between rising/falling wind is on the order of 20%, while the relative difference in *W_f_* between rising/falling wind is on the order of 50% (Fig. 3; Table S1). If *k_b_* scales proportionally with *W_f_*, we expect *k_b_* to contribute roughly 40% of the total *K*_CO2,660_ (20%/0.5), which is more consistent with Eq. 4 (as well as (11, 37)), and less than the prediction by Fairall et al. (17).

**Fig. 5.**
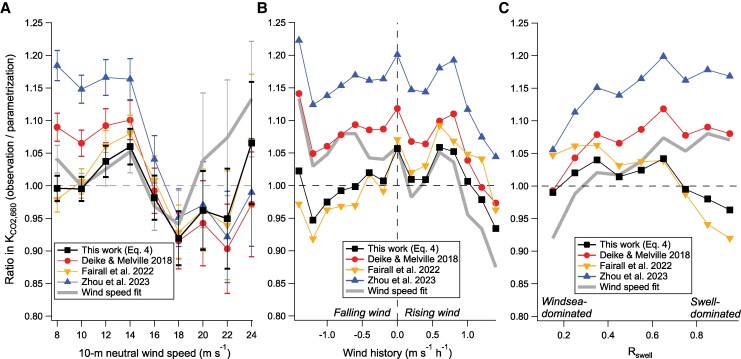
A) Bin-averaged ratio between observed and parameterized *K*_CO2,660_ at wind speeds above 7 m s^−1^ where wave breaking becomes important, with error bars indicate SE. B) Same as A), but in bins of wind history. Error bars not shown to avoid visual clutter. C) Same as B), but in bins of *R*_swell_.

Out of these parameterizations, Eq. 4 generally matches observations the best when averaged in bins of wind speed, wind history, and swell impact. This is demonstrated in Fig. 5 as the ratio between observed and parameterized *K*_CO2,660_, with the most optimal parameterization giving a ratio of unity during all conditions and showing no trend. Parametrizations from Deike and Melville (11) and Zhou et al. (37) underpredict *K*_CO2,660_ at moderate to high wind speeds (8 to 16 m s^−1^) and slightly overpredict *K*_CO2,660_ at even higher wind speeds (Fig. 5A), probably related to the nonlinearity in their formula as shown in Fig. 4A. In contrast, Eq. 4 and Fairall et al. (17) both reproduce the mean wind speed dependence in *K*_CO2,660_ reasonably well.

Figure 5B, like Fig. 3B, illustrates the wind history dependence in *K*_CO2,660_ at wind speeds over 7 m s^−1^ (i.e. observation/wind speed fit is negatively correlated with wind history). This wind history dependence is well accounted for by Eq. 4. Formulations from Deike and Melville (11) and Zhou et al. (37) also account for the relative wind history trend, but on average underestimates *K*_CO2,660_. The Fairall et al. (17) formula, which has a ∼60% larger *k_b_* than Eq. 4, appears to inadvertently over-correct for this wind history dependence.

Observed *K*_CO2,660_ at a given wind speed appears to be greater during swell-dominated conditions (here indicated by *R*_swell_ = (*Hs__s_*_well_/*H_s__*_total_)^2^) than windsea-dominated conditions (Fig. 5C). This could be another sign of the wind history dependence, and/or be related to the idea that breaking in aged seas is less energetic but more conducive to producing longer-lasting bubbles (41). Parametrizations from Deike and Melville (11) and Zhou et al. (37) do not reduce this swell dependence, while Fairall et al. (17) appears to over-correct for it. Equation 4 is again the most optimal. We note that re-tuning Eqs. 1 and 2 with the latest observations only very marginally improves their performance and does not alter the general trends above.

Turning our attention over to short term variability, Eq. 4 gives a *R*^2^ of 0.753 when applied to the hourly testing dataset for the ML model, which is significantly better than wind speed (0.693 for the same dataset) but falls short compared with the ML model (0.813), probably because there are other subtle wind/wave dependent effects that Eq. 4 does not consider. Nevertheless, we can estimate the sea state driven *K*_CO2,660_ variability by evaluating the different gas transfer parameterizations at the ambient u_*_ and *H_s_* (Fig. 1C). At *U*_10*n*_ of 15 m s^−1^, the standard deviation (*σ*) of the predicted *K*_CO2,660_ from Eq. 4 is about 10 cm h^−1^. This sea state effect can account for over a third of the variance (*σ*^2^) in observed *K*_CO2,660_ at these high wind speeds once measurement noise is considered (Fig. 1A and D). In Eq. 2, the use of u_v*_ instead of u_*_ to fit *k_d_* necessitates a ∼60% larger *k_b_* compared with Eq. 4. The sea state driven variability in *K*_CO2,660_ from Fairall et al. (17), even without considering measurement noise, exceeds the variability in observed *K*_CO2,660_ at high wind speeds (Fig. 1D), again hinting that their *k_b_* may be too large.

## Concluding remarks

Parameterizing *K*_660_ as a simple function of wind speed, given sufficient observations across a wide range of sea states, can yield a reasonable “climatological fit” in the mean. This study reveals significant sea state dependent variability in observed hourly *K*_CO2,660_ that is due to bubble-mediated transfer—a process that differs between gases of different solubility. We provide a new method of parameterizing *K*_CO2,660_ using wind/wave data (Eqs. 3 and 4) that explains more observed variability than a wind speed fit, and better accounts for the effects of wind history and swell than other recently proposed wind-wave parameterizations.

It is worth noting that Eq. 4, developed here for CO_2_, should not be used “as is” to predict transfer of other gases (e.g. DMS). A universal framework for modeling gas transfer requires the specification of the solubility dependence in *k_b_* (e.g. (8, 42)), which is not very well understood. Quantification of *K*_660_ of another waterside-controlled gas (in addition to CO_2_ and DMS) should help to further constrain the impact of bubble-mediated transfer.

This work has made use of modeled wave data to decipher patterns in field *K*_CO2,660_ measurements. There have been very few field campaigns with concurrent wave, whitecap/bubble, and *K*_660_ measurements. Further improvements in mechanistic understanding require more of these concurrent measurements under a wide range of conditions. We have used ML techniques here to mostly estimate the total variability that is explainable by wind and wave data, as well as identify wave parameters that influence *K*_CO2,660_. The total number of direct *K*_CO2,660_ observations made by the international community to date is still rather limited (a few thousand hours). We anticipate that more *K*_CO2,660_ observations will likely lead to further improvements in the ML-based predictions of gas transfer. Recent developments in buoy-based flux measurements (43) appear to offer a highly promising approach to drastically increase the number of *K*_CO2,660_ observations.

## Supplementary Material

pgae389_Supplementary_Data

## Data Availability

This article mostly makes use of already published data: Yang et al. (2) synthesis of *K*_CO2,660_ and wave data; HiWinGS *K*_DMS,660_ data from Blomquist et al. (5); whitecap data from Scanlon and Ward (32), Callaghan et al. (33), and Brumer et al. (39). New *K*_CO2,660_ observations from Dong et al. (24, 25) along with wave data are included in the Supplementary material of this article.
