# Supplementary material for: Deciphering the variability in air-sea gas transfer due to sea state and wind history
Source: PNAS Nexus. 2024 Sep 4;3(9):pgae389. doi: 10.1093/pnasnexus/pgae389 (PMC11410043; doi:10.1093/pnasnexus/pgae389)
Supplement: pgae389_Supplementary_Data [file pgae389_supplementary_data.docx]

**Deciphering the variability in air-sea gas transfer due to sea state and wind history**

Mingxi Yang^1*^, David Moffat^1^, Yuanxu Dong^2,3^, Jean-Raymond Bidlot^4^

1. Plymouth Marine Laboratory, Prospect Place, PL1 3DH, Plymouth, UK
2. Marine Biogeochemistry Research Division, GEOMAR Helmholtz Centre for Ocean Research Kiel, Kiel, Germany
3. Institute of Environmental Physics, Heidelberg University, Im Neuenheimer Feld 229, 69120 Heidelberg, Germany
4. European Centre for Medium-Range Weather Forecasts, Shinfield Park, Reading, United Kingdom

^*^Correspondence to: Mingxi Yang, Plymouth Marine Laboratory, United Kingdom ([miya@pml.ac.uk](mailto:miya@pml.ac.uk))

**Supplement**

*Machine learning (ML) analysis of gas transfer observations*


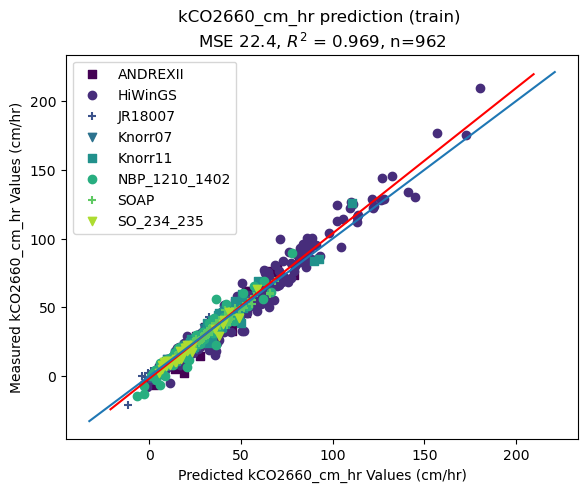


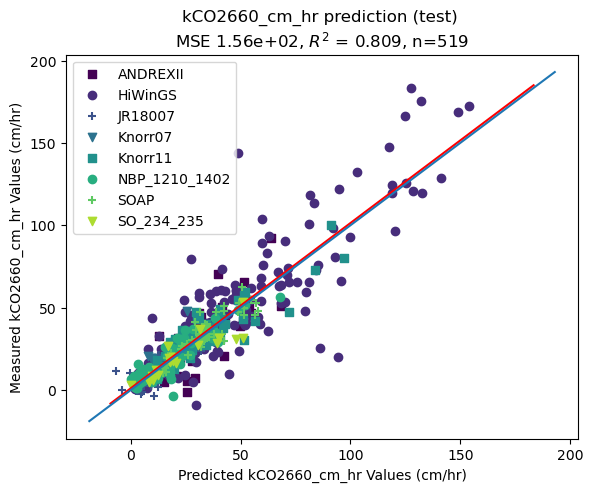


Figure S1. Performance of the ML model for the training dataset (top) and testing dataset (bottom). The mean squared error (MSE in cm^2^ hr^-2^) and the coefficient of determination (R^2^) are labeled on top of the panels. Blue line represents 1:1, while the red line is linear regression. The Pearson correlation coefficients (r^2^) are 0.97 and 0.81 for the training and testing datasets, respectively.


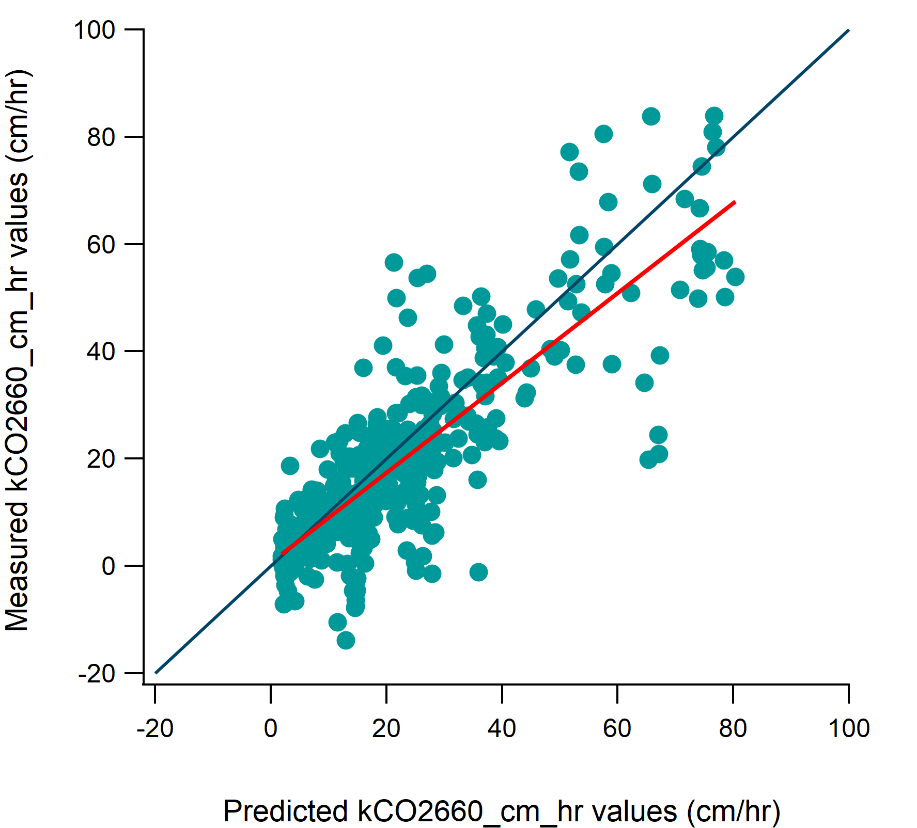


Figure S2. Performance of the ML model for the additional validation dataset from Dong et al. 2024, which was not used to train the model. The ML model overpredicts relative to the observations by 14% (R^2^ = 0.66) but the Pearson correlations between observations and predicts remain high (r^2^ = 0.74).

To understand why the ML model can better predict *K_CO2,660_* than wind speed, we interrogate the factors contributing to the predictions from ML via the SHAP value. SHAP (SHapley Additive exPlanations) is a game theory approach to understand how the ML model makes predictions. This approach looks at all the input variables and evaluates which variables has the largest impact on the model output.


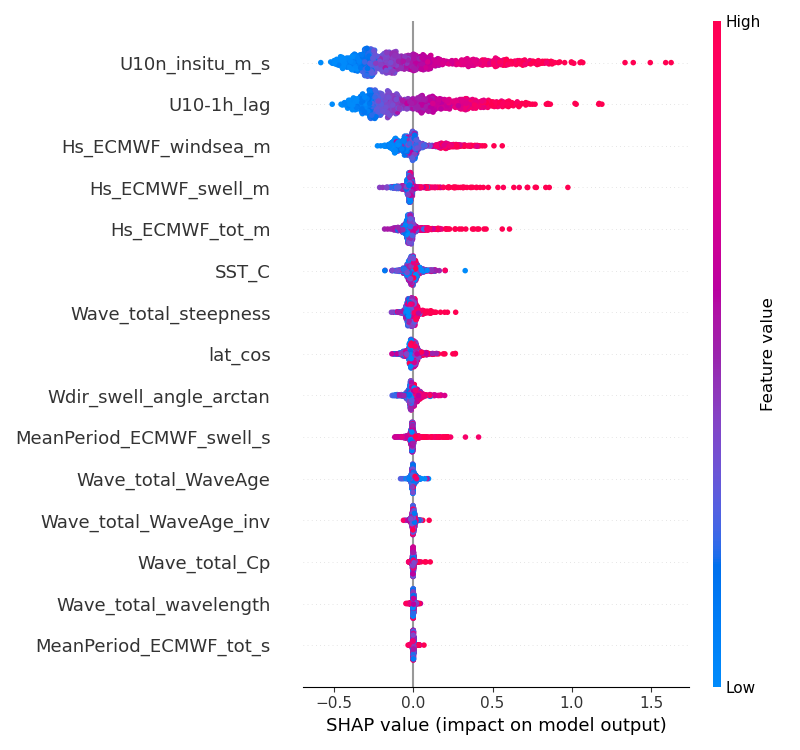


Figure S3 shows the input variables for the ML model along the Y-axis, with the variables ranked by how large their impact is on the model prediction. The X-axis shows the SHAP value, which is the scale of the output prediction from the model. Unsurprisingly we see that *U*_10_*_n_* is the most important variable for the ML model to make a prediction – the largest positive predictions of *K_CO2,660_* are for high *U*_10_*_n_* (in red), while the lowest predictions of *K_CO2,660_* are for low *U*_10_*_n_* (blue). Significant wave heights are also correlated with *K_CO2,660_* and show relatively large SHAP value, while inputs such as wave age and wave steepness have little impact on the predicted *K_CO2,660_*.

**ML model input parameters:**

*In situ*

U10n – In situ 10-m neutral windspeed

U10-1hr-lag – U10n from the previous hour

SST_C – In situ bulk sea surface temperature

Latitude – Cos(latitude in degrees / 180 $\pi$)

*ECMWF (high resolution ECWAM) wave parameters (see Yang et al. 2022)*

Hs_windsea – Significant wave height (windsea)

Hs_swell – Significant wave height of (windsea)

Hs_total – Significant wave height (total)

Wdir_swell – Arctan of the difference between the wind direction and swell direction

MeanPeriod – Energy mean period of total waves

MeanPeriod_swell – Energy mean period of swell

Total_Wavelength – Wavelength from total wave period

Wave_total_steepness – Steepness of waves

Wave_Cp – Phase speed of waves.

WaveAge – Wave age

WaveAge – Inverse wave age

*Calculations of wave parameters:*

Wavelength($\lambda$, in m) is computed from the energy mean period (P_m_) of the total waves (in s) following the deep water dispersion relationship: $\lambda$ = *g* P_m_ ^2^ / (2 $\pi$), where *g* is gravity (m s^-2^). Total wave steepness is the ratio between the total significant wave height and $\lambda$. Wave age is computed as the ratio between the phase speed (*Cp*) of waves and *U*_10_*_n_*, where

*Cp* = (*g*/(2 $\pi$ / $\lambda$))^0.5^. Inverse wave age is simply 1/wave age.

*­Variability in significant wave heights (windsea, swell, total) as a function of wind history*


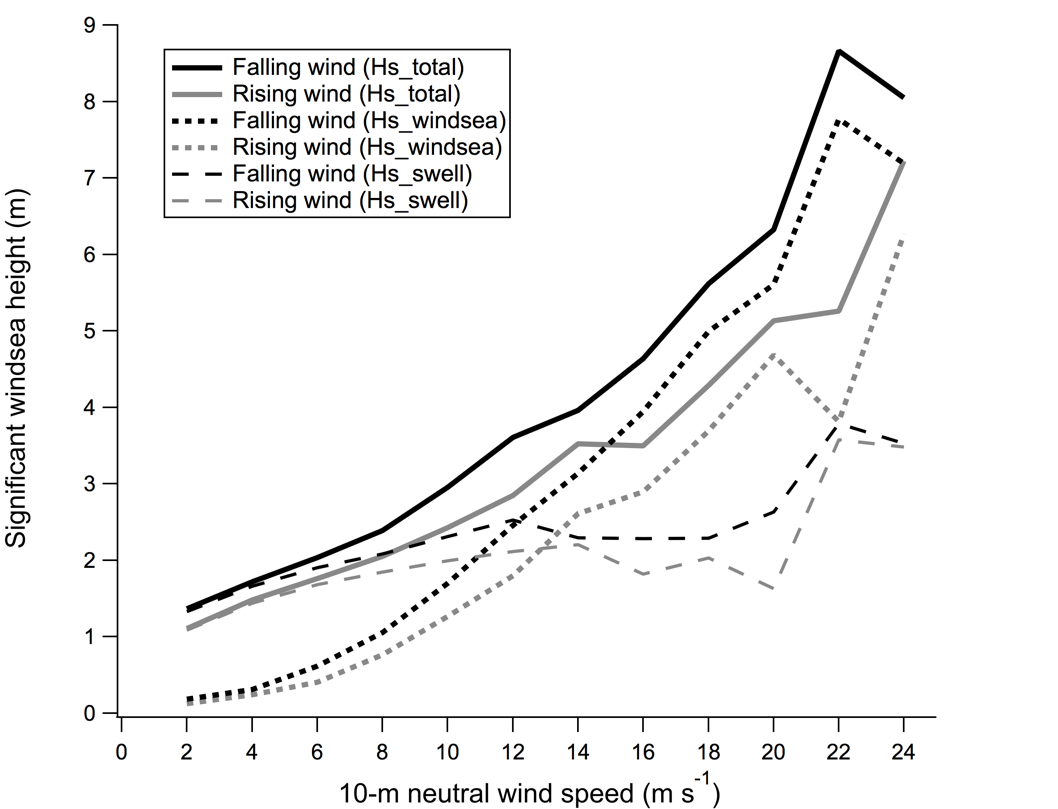


Figure S4. Bin-averages of significant wave heights over all datasets considered. Unsurprisingly, significant wave heights are on average greater during falling wind than during rising wind.

Table S1. Relative enhancement (%) in *K_CO2,660_* (all data, threshold of |0.5 m s^-1^ h^-1^|) and *W_f_* (average of Knorr11 cruise and Callaghan et al. 2008) during conditions of falling winds vs. rising winds, in different wind speed bins.

|  | 7-9 m s^-1^ | 9-11 m s^-1^ | 11-13 m s^-1^ | 13 – 15 m s^-1^ | 15-17 m s^-1^ | 17-19 m s^-1^ | 19-21 m s^-1^ | 21-23 m s^-1^ |
| --- | --- | --- | --- | --- | --- | --- | --- | --- |
| *K_CO2,660_* | 3 | 10 | 7 | 35 | 34 | 39 | 35 | 16 |
| *W_f_* | 49 | 65 | 62 | 41 | 61 | 38 | 34 | - |

Table S2. Pearson correlation coefficients (r) between *K_CO2,660_* and wind history within different wind speed bins (number of hours italicized). Wind histories (d*U*_10_*_n_*/dt) is computed as the linear trend of the preceding 3 hours. Hs_windsea, Hs_swell, and Hs_total indicate windsea, swell, and total contributions to significant wave height. Significant correlations (95% confidence) are bold faced. Correlations with wave heights are almost always positive and higher with increasing *U*_10_*_n_*. Correlations with wind history are generally negative.

|  | 2-3 m s^-1^ (*86*) | 3-4 m s^-1^ (*113*) | 4-5 m s^-1^ (*155*) | 5-6 m s^-1^ (*156*) | 6-7 m s^-1^ (*188*) | 7-8 m s^-1^ (*216*) | 8-9 m s^-1^ (*225*) | 9-10 m s^-1^ (*198*) |
| --- | --- | --- | --- | --- | --- | --- | --- | --- |
| Hs_windsea | 0.128 | 0.052 | 0.056 | 0.120 | **0.254** | **0.221** | -0.033 | 0.134 |
| Hs_swell | **0.213** | **0.229** | 0.007 | 0.083 | 0.115 | **0.185** | **0.174** | **0.180** |
| Hs_total | **0.217** | **0.223** | 0.024 | 0.090 | 0.133 | **0.217** | **0.154** | **0.201** |
| dU10n/dt | -0.211 | **-0.220** | **-0.173** | -0.091 | -0.128 | -0.030 | 0.011 | 0.113 |

|  | 10-11 m s^-1^ (*211*) | 11-12 m s^-1^ (*166*) | 12-13 m s^-1^ (*123*) | 13-14 m s^-1^ (*104*) | 14-15 m s^-1^ (*83*) | 15-16 m s^-1^ (*58*) | 16-17 m s^-1^ (*42*) | 17-18 m s^-1^ (*27*) |
| --- | --- | --- | --- | --- | --- | --- | --- | --- |
| Hs_windsea | **0.144** | **0.166** | **0.338** | **0.269** | **0.371** | **0.318** | 0.258 | **0.463** |
| Hs_swell | **0.298** | 0.103 | **0.453** | **0.604** | **0.457** | 0.247 | **0.619** | **0.458** |
| Hs_total | **0.309** | 0.146 | **0.464** | **0.533** | **0.474** | **0.319** | **0.461** | **0.569** |
| dU10n/dt | -0.122 | -0.021 | -0.167 | -0.121 | **-0.365** | -0.078 | -0.090 | -0.355 |

Data from Dong et al. (2024a), filtered for air-sea CO_2_ fugacity difference of at least 20 µatm, supplemented here with wave data from ECMWF.

Dong, Y. et al. (2024a). Data from: Direct observational evidence of strong CO2 uptake in the Southern Ocean. Dryad. <https://doi.org/10.5061/dryad.b2rbnzspm>

Eddy covariance CO2 flux and transfer velocity data from other JCR ORCHESTRA cruises (TS19, TS20, JCR19001, JCR30001)

Missing fields noted by NaN

Data column descriptions:

YearMonthDay_TimeUTC : timestamp for middle of hourly average

lat_deg : decimal latitude

lon_deg : decimal longitude

CO2flux_mmol_m2_d : eddy covariance CO2 flux, mmol/m2/d

kCO2660_cm_hr : CO2 transfer velocity at Sc=660, cm/hr

kCO2_cm_hr : CO2 transfer velocity at ambient Sc

ustar_insitu_m_s : friction velocity from in situ wind measurements, m/s

U10n_insitu_m_s : 10-m neutral wind speed from in situ wind measurements, m/s

SST_C : bulk underway water temperature, deg C

Tskin_C : skin temperature (bulk temperature corrected by COARE3.5 model cool skin effect), deg C

sal : salinity (assumed to be 35 if missing)

dfCO2_uatm : sea-air difference in fCO2 (=fCO2w - fCO2a), uatm

sol_CO2_bulk_mole_m3_atm : CO2 solubility computed at bulk water temperature, moles/m3/atm

sol_CO2_skin_mole_m3_atm : CO2 solubility computed at skin temperature, moles/m3/atm

DCCO2_mmol_m3 : sea-air difference in CO2 concentration (=sol_CO2_bulk_mole_m3_atm*fCO2w - sol_CO2_skin_mole_m3_atm*fCO2a), mmol/m3

Sc_CO2_bulk : Schmidt number of CO2 computed at bulk water temperature

U10n_ECMWF_m_s : 10-m neutral wind speed from ECMWF model, m/s

ustar_ECMWF_m_s : friction velocity from ECMWF model, m/s

Hs_ECMWF_tot_m : significant wave height of total waves from ECMWF model, m

RHw0 : wave Reynolds number at 20 deg C computed from Hs_ECMWF_tot_m and ustar_insitu_m_s

MSS_ECWAM : mean squared slope of waves from ECMWF model

Hs_ECMWF_windsea_m : significant wave height of wind sea, m

Hs_ECMWF_swell_m : significant wave height of swell, m

MeanPeriod_ECMWF_tot_s : mean wave period of total waves, s

MeanPeriod_ECMWF_swell_s : mean wave period of swell, s

MeanPeriod_ECMWF_windsea_s : mean wave period of wind sea, s

Wdir_ECMWF_deg : wind direction from ECMWF model, deg

Swell_dir_ECMWF_deg : swell direction from ECMWF model, deg

YearMonthDay_TimeUTC, lat_deg, lon_deg, CO2flux_mmol_m2_d, kCO2660_cm_hr, kCO2_cm_hr, ustar_insitu_m_s, U10n_insitu_m_s, SST_C, Tskin_C, sal, dfCO2_uatm, sol_CO2_bulk_mole_m3_atm, sol_CO2_skin_mole_m3_atm, DCCO2_mmol_m3, Sc_CO2_bulk, U10n_ECMWF_m_s, ustar_ECMWF_m_s, Hs_ECMWF_tot_m, RHw0, MSS_ECWAM, Hs_ECMWF_windsea_m, Hs_ECMWF_swell_m, MeanPeriod_ECMWF_tot_s, MeanPeriod_ECMWF_swell_s, MeanPeriod_ECMWF_windsea_s, Wdir_ECMWF_deg, Swell_dir_ECMWF_deg

2019-10-21 00:30:00, -51.5951, -50.5087, -4.47423, 8.45896, 6.06274, 0.257761, 7.17949, 8.01148, NaN, 33.8674, NaN, NaN, NaN, NaN, NaN, 5.91601, NaN, 2.32125, 823221, 0.022753, 1.15219, 1.87474, 8.12151, 9.12565, 4.27686, 273.808, 271.838

2019-11-25 15:30:00, -58.3056, -43.9682, -11.5499, 13.8998, 7.88678, 0.3868, 10.455, 0.4986, 0.422465, 33.867, -81.7806, 63.9819, 63.9433, -6.12364, 2062.13, NaN, NaN, NaN, NaN, NaN, NaN, NaN, NaN, NaN, NaN, NaN, NaN

2019-11-25 16:30:00, -58.1435, -43.7329, -17.0556, 15.5119, 8.79396, 0.350675, 9.7405, 0.472, 0.375324, 33.8186, -136.808, 64.1131, 64.0767, -8.11607, 2068.27, NaN, NaN, NaN, NaN, NaN, NaN, NaN, NaN, NaN, NaN, NaN, NaN

2019-11-25 17:30:00, -57.9875, -43.5024, -13.084, 12.4811, 7.08094, 0.336975, 9.45777, 0.494875, 0.397252, 33.8271, -126.618, 64.0515, 64.0193, -7.73217, 2065.14, NaN, NaN, NaN, NaN, NaN, NaN, NaN, NaN, NaN, NaN, NaN, NaN

2019-11-25 18:30:00, -57.8516, -43.3066, -16.2908, 15.7844, 8.96304, 0.343725, 9.59255, 0.522725, 0.445997, 33.8688, -123.343, 63.9053, 63.8844, -7.59819, 2058.08, NaN, NaN, NaN, NaN, NaN, NaN, NaN, NaN, NaN, NaN, NaN, NaN

2019-11-25 19:30:00, -57.7239, -43.1226, -9.45895, 8.68924, 4.92905, 0.271125, 8.03047, 0.490675, 0.385379, 33.8276, -129.996, 64.0652, 64.0486, -8.03074, 2065.87, NaN, NaN, NaN, NaN, NaN, NaN, NaN, NaN, NaN, NaN, NaN, NaN

2019-11-25 20:30:00, -57.5966, -42.9419, -11.806, 11.0704, 6.29727, 0.259525, 7.77227, 0.5774, 0.432338, 33.7822, -132.992, 63.9539, 63.947, -7.85648, 2059.14, NaN, NaN, NaN, NaN, NaN, NaN, NaN, NaN, NaN, NaN, NaN, NaN

2019-11-25 21:30:00, -57.4706, -42.7621, -13.0853, 13.2959, 7.59681, 0.273125, 8.07995, 0.715425, 0.644204, 33.7952, -115.989, 63.4237, 63.4198, -7.19721, 2031.17, NaN, NaN, NaN, NaN, NaN, NaN, NaN, NaN, NaN, NaN, NaN, NaN

2019-11-25 22:30:00, -57.3463, -42.5841, -14.6883, 13.6851, 7.78575, 0.237925, 7.2726, 0.581925, 0.517223, 33.7735, -132.325, 63.7526, 63.7397, -7.88198, 2048.27, NaN, NaN, NaN, NaN, NaN, NaN, NaN, NaN, NaN, NaN, NaN, NaN

2019-11-25 23:30:00, -57.2272, -42.4135, -11.7072, 13.6975, 7.82826, 0.199667, 6.30793, 0.7235, 0.582573, 33.8085, -102.085, 63.4704, 63.5669, -6.2559, 2033.89, NaN, NaN, NaN, NaN, NaN, NaN, NaN, NaN, NaN, NaN, NaN, NaN

2019-12-02 02:30:00, -53.78, -38.162, -7.392, 11.6757, 7.04876, 0.19075, 6.06952, 2.4432, 2.33312, 33.8741, -60.3279, 59.3571, 59.4358, -4.38192, 1819.47, 4.76021, 0.157696, 1.6959, 265847, 0.014175, 0.26995, 1.67345, 8.74435, 8.91725, 2.33973, 144.888, 345.911

2019-12-02 04:30:00, -53.7518, -37.9356, -4.60573, 13.3602, 8.04719, 0.1213, 4.05113, 2.37058, 2.27346, 33.8623, -37.0469, 59.5008, 59.5732, -2.39061, 1826.71, 5.18812, 0.168689, 1.75285, 303499, 0.01435, 0.303475, 1.72487, 8.84822, 9.05492, 2.44453, 154.774, 342.858

2019-12-02 06:30:00, -53.8624, -37.9356, -2.80395, 8.1892, 4.91745, 0.077825, 2.559, 2.27387, 2.11865, 33.8447, -30.2911, 59.888, 59.9278, -2.38826, 1846.47, 7.08951, 0.250548, 1.788, 452901, 0.0148333, 0.4985, 1.71257, 8.7977, 9.29387, 3.11443, 164.465, 338.876

2019-12-02 07:30:00, -53.703, -37.9344, -2.9816, 8.71996, 5.26075, 0.140775, 4.65897, 2.42165, 2.25726, 33.8625, -41.1959, 59.5297, 59.6096, -2.37306, 1828.21, 7.33767, 0.266742, 1.89063, 507502, 0.016025, 0.594425, 1.79065, 8.69492, 9.29937, 3.29397, 165.183, 339.27

2019-12-02 09:30:00, -53.8584, -37.9383, -2.74395, 7.39311, 4.45086, 0.079775, 2.6098, 2.35505, 2.22579, 33.8462, -37.7224, 59.648, 59.6854, -2.5797, 1834.06, 9.13311, 0.348441, 1.9029, 676550, 0.01895, 0.965875, 1.6126, 8.04235, 9.5836, 3.98855, 164.619, 333.37

2019-12-02 14:30:00, -53.6333, -37.9912, 1.5578, -4.24166, -2.60552, 0.07385, 2.4239, 2.9917, 2.78204, 33.8607, -42.0617, 58.3537, 58.4472, -2.50684, 1767.66, 10.3253, 0.439231, 2.21737, 959220, 0.02565, 1.41017, 1.70752, 7.43883, 9.31488, 4.68823, 174.062, 345.389

2019-12-02 16:30:00, -53.5821, -38.0117, -7.40293, 18.0762, 11.0541, 0.35575, 9.82445, 2.84982, 2.75457, 33.8636, -45.5332, 58.4365, 58.5064, -2.79706, 1771.95, 10.318, 0.434008, 2.24543, 971610, 0.02795, 1.51445, 1.6551, 7.17267, 9.12515, 4.8564, 176.65, 352.343

2019-12-02 18:30:00, -53.7187, -38.0202, -14.2366, 29.8517, 18.1807, 0.4102, 10.9097, 2.72055, 2.63202, 33.8656, -58.9157, 58.7049, 58.7748, -3.26973, 1785.75, 10.825, 0.45009, 2.1301, 959171, 0.028125, 1.47728, 1.5309, 6.96262, 8.97358, 4.80655, 178.585, 356.711

2019-12-02 22:30:00, -53.5976, -38.0044, -21.8738, 48.7734, 29.6071, 0.39805, 10.6675, 2.61658, 2.49137, 33.8675, -52.0549, 59.0033, 59.0852, -3.0886, 1801.13, 10.7185, 0.446998, 2.0994, 932579, 0.0319, 1.56018, 1.40375, 6.49215, 8.45702, 4.92875, 182.81, 23.3649

2019-12-03 00:30:00, -53.7077, -38.2031, -18.8383, 33.9091, 20.5556, 0.5128, 12.7977, 2.57295, 2.44, 33.8717, -59.103, 59.1181, 59.1981, -3.83255, 1807.11, 10.4132, 0.433064, 2.00195, 855924, 0.0322, 1.51968, 1.3022, 6.30808, 8.24773, 4.91545, 183.809, 28.8284

2019-12-03 04:30:00, -53.7946, -38.5899, -5.0487, 9.37003, 5.5951, 0.28255, 8.2844, 2.09747, 1.97741, 33.8958, -63.5422, 60.1667, 60.2329, -3.77277, 1861.75, 8.61814, 0.32505, 1.75675, 569327, 0.02855, 1.14715, 1.32915, 6.2755, 7.51498, 4.64558, 210.085, 110.615

2019-12-03 08:30:00, -53.469, -39.1628, -23.4205, 39.5461, 24.0979, 0.3683, 10.0731, 2.73765, 2.61548, 33.8748, -76.6295, 58.716, 58.8085, -4.06176, 1786.47, 9.54974, 0.382137, 1.90572, 718367, 0.0324, 1.36777, 1.3262, 6.13772, 7.44262, 4.94077, 238.353, 126.774

2019-12-03 09:30:00, -53.3437, -39.3434, -22.3121, 41.6163, 25.3442, 0.411125, 10.9153, 2.71868, 2.55448, 33.8787, -55.6271, 58.8455, 58.942, -3.68485, 1793.18, 9.69984, 0.37316, 1.94013, 732563, 0.032625, 1.40237, 1.33903, 6.17085, 7.44245, 5.03822, 241.593, 130.719

2019-12-03 10:30:00, -53.2206, -39.5213, -23.3591, 31.8178, 19.405, 0.4574, 11.7934, 2.76445, 2.62121, 33.8877, -85.5014, 58.6975, 58.7919, -5.03462, 1785.72, 10.6084, 0.418362, 1.99855, 849701, 0.033775, 1.51675, 1.29777, 6.15485, 7.56278, 5.1507, 242.199, 124.874

2019-12-03 11:30:00, -53.0971, -39.6987, -33.4094, 44.2244, 26.9218, 0.466425, 11.9614, 2.70615, 2.58897, 33.8946, -89.8687, 58.7777, 58.8609, -5.18619, 1789.94, 10.6348, 0.438886, 2.0644, 898525, 0.035025, 1.6103, 1.29015, 6.14562, 7.61523, 5.23113, 243.25, 126.199

2019-12-03 12:30:00, -52.9752, -39.8717, -39.071, 44.3283, 27.0842, 0.4658, 11.9518, 2.82218, 2.64573, 33.9173, -96.4459, 58.6487, 58.7289, -6.04235, 1783.67, 10.9305, 0.445135, 2.11905, 945660, 0.035675, 1.69762, 1.26487, 6.1676, 7.67865, 5.35635, 243.018, 123.556

2019-12-03 13:30:00, -52.8573, -40.0326, -29.2293, 25.5591, 15.6999, 0.450525, 11.64, 2.99117, 2.89591, 33.9316, -138.641, 58.0975, 58.1778, -7.77417, 1755.65, 10.7909, 0.443072, 2.1575, 939638, 0.035875, 1.75223, 1.25712, 6.20572, 7.70045, 5.4679, 242.535, 124.1

2019-12-03 16:30:00, -52.8053, -40.1519, -41.1888, 32.1498, 19.592, 0.3446, 9.59275, 2.73952, 2.61628, 33.9151, -158.684, 58.7332, 58.7945, -8.79108, 1787.98, 9.75152, 0.378367, 2.19515, 820902, 0.03425, 1.69582, 1.39075, 6.40623, 7.55143, 5.6712, 244.332, 165.905

2019-12-03 17:30:00, -52.8051, -40.1598, -35.0093, 27.0816, 16.4842, 0.3403, 9.51942, 2.70245, 2.57684, 33.9171, -160.217, 58.8195, 58.8809, -8.88173, 1792.45, 9.5053, 0.361583, 2.20195, 788092, 0.03345, 1.64625, 1.46018, 6.49235, 7.51938, 5.7212, 242.243, 184.079

2019-12-03 18:30:00, -52.8075, -40.1651, -31.2689, 23.9789, 14.5974, 0.312075, 8.92757, 2.7064, 2.57735, 33.9172, -161.367, 58.8198, 58.8797, -8.95955, 1792.47, 9.14903, 0.345096, 2.20713, 750797, 0.03265, 1.5665, 1.54775, 6.57995, 7.50552, 5.69797, 239.27, 201.518

2019-12-03 19:30:00, -52.8061, -40.1619, -21.6514, 16.7246, 10.1889, 0.2411, 7.3213, 2.73007, 2.58635, 33.9159, -161.135, 58.7849, 58.8602, -8.89063, 1790.65, 8.48213, 0.320083, 2.20402, 692398, 0.031725, 1.32012, 1.7464, 6.67225, 7.47257, 5.20702, 237.898, 218.126

2019-12-03 20:30:00, -52.804, -40.1572, -24.1196, 18.9319, 11.54, 0.26265, 7.82877, 2.74747, 2.60228, 33.9149, -158.04, 58.7323, 58.8254, -8.74242, 1787.93, 7.78115, 0.291161, 2.19015, 628200, 0.0306, 1.0274, 1.926, 6.77088, 7.42972, 4.4406, 239, 225.776

2019-12-03 21:30:00, -52.804, -40.1572, -26.9638, 21.0631, 12.8339, 0.280725, 8.23392, 2.73468, 2.5891, 33.9143, -158.057, 58.7485, 58.8547, -8.78619, 1788.75, 7.66028, 0.278786, 2.17255, 603796, 0.02955, 0.9531, 1.95077, 6.85922, 7.48605, 4.30987, 239.166, 226.903

2019-12-03 22:30:00, -52.804, -40.1572, -24.8038, 19.2164, 11.6939, 0.243925, 7.39118, 2.6949, 2.54375, 33.9146, -159.542, 58.8356, 58.9548, -8.87016, 1793.23, 7.59519, 0.276299, 2.1559, 590029, 0.028775, 0.920025, 1.94785, 6.92625, 7.53245, 4.28063, 240.176, 227.096

2019-12-03 23:30:00, -52.804, -40.1572, -19.7026, 15.0951, 9.18376, 0.210025, 6.558, 2.68737, 2.52875, 33.916, -161.437, 58.8582, 58.9876, -8.97274, 1794.42, 7.26124, 0.264379, 2.13532, 555810, 0.028025, 0.815525, 1.97142, 6.98645, 7.51548, 3.97918, 244.787, 227.267

2019-12-04 00:30:00, -52.807, -40.1769, -16.7159, 12.5971, 7.67957, 0.170075, 5.509, 2.75157, 2.57024, 33.9159, -164.603, 58.7692, 58.8958, -9.11176, 1789.84, 6.62585, 0.240505, 2.10577, 495514, 0.0271, 0.671825, 1.9933, 7.0539, 7.46445, 3.5576, 255.945, 227.493

2019-12-04 01:30:00, -52.8076, -40.2204, -19.1292, 14.3071, 8.75168, 0.17845, 5.73088, 2.85902, 2.70565, 33.9212, -166.869, 58.493, 58.5962, -9.14371, 1775.74, 6.25171, 0.220403, 2.06962, 454114, 0.02615, 0.577975, 1.9867, 7.1205, 7.45615, 3.3077, 270.306, 227.567

2019-12-04 02:30:00, -52.8011, -40.2604, -16.1758, 12.1302, 7.43301, 0.199975, 6.31793, 2.91427, 2.77147, 33.9286, -164.199, 58.3705, 58.4498, -9.10368, 1769.57, 6.51794, 0.224122, 2.03748, 464603, 0.0255, 0.623625, 1.93602, 7.15508, 7.54765, 3.48423, 277.575, 226.565

2019-12-04 03:30:00, -52.7926, -40.3052, -24.5157, 18.3918, 11.2734, 0.305075, 8.74068, 2.9241, 2.80285, 33.9344, NaN, 58.3386, 58.3796, -9.09576, 1768.03, 7.80116, 0.264674, 2.02903, 559976, 0.02575, 0.9041, 1.79047, 7.1072, 7.7496, 4.3871, 272.548, 220.089

2019-12-06 23:30:00, -53.3601, -38.116, -3.04567, 5.54492, 3.34059, 0.099275, 3.30563, 2.37767, 2.25179, 33.8365, NaN, 59.5611, 59.6299, -3.81251, 1829.41, 3.67473, 0.117227, 1.9675, 229614, 0.0133, 0.1449, 1.96198, 9.7252, 9.77575, 1.7339, 288.148, 256.858

2019-12-07 01:30:00, -53.5105, -38.0704, -3.5014, 5.45099, 3.33455, 0.11795, 3.93372, 2.86062, 2.74367, 33.8674, -83.538, 58.4353, 58.5291, -4.38732, 1771.95, 3.19409, 0.100788, 1.9933, 200214, 0.0122, 0.100025, 1.9907, 10.2439, 10.2724, 1.49103, 296.611, 256.44

2019-12-07 09:30:00, -53.6968, -37.7859, -2.65767, 4.7961, 2.94278, 0.27305, 8.04812, 2.9561, 2.83203, 33.837, -68.4017, 58.2582, 58.3452, -3.77484, 1762.41, 5.25931, 0.158402, 1.95575, 327818, 0.0103, 0.282025, 1.9331, 11.0125, 11.2014, 2.70748, 230.892, 256.135

2019-12-07 10:30:00, -53.5361, -37.84, -11.2322, 22.3695, 13.7199, 0.353575, 9.7678, 2.9431, 2.80904, 33.8642, -51.5693, 58.2929, 58.3872, -3.42282, 1764.61, 6.53671, 0.208727, 2.10387, 453075, 0.0109, 0.4449, 2.05295, 11.1131, 11.4954, 3.3576, 215.631, 253.348

2019-12-07 11:30:00, -53.3758, -37.8937, -19.4406, 25.6731, 15.7144, 0.3467, 9.63255, 2.87947, 2.67821, 33.8571, -81.3353, 58.5752, 58.6758, -5.1848, 1778.96, 7.36842, 0.250659, 2.19545, 554254, 0.011575, 0.60805, 2.1064, 11.0166, 11.6417, 3.72622, 213.869, 252.073

2019-12-07 12:30:00, -53.2197, -37.9359, -20.299, 22.3362, 13.7785, 0.3146, 8.9556, 3.1258, 3.02107, 33.9143, -111.11, 57.79, 57.9122, -6.14949, 1739.68, 7.64441, 0.263821, 2.26213, 598857, 0.012275, 0.73145, 2.13867, 10.8553, 11.6742, 3.96515, 212.911, 251.599

2019-12-07 14:30:00, -53.3372, -37.7687, -12.3369, 14.3063, 8.74434, 0.318225, 9.0401, 2.8341, 2.64661, 33.8682, -98.9639, 58.6003, 58.7419, -5.90576, 1780.43, 7.4114, 0.260883, 2.28973, 593821, 0.0134, 0.86465, 2.11945, 10.4543, 11.5066, 4.21007, 220.995, 253.094

2019-12-07 15:30:00, -53.4988, -37.7141, -14.6278, 18.2588, 11.1796, 0.3343, 9.37235, 2.8894, 2.75684, 33.8762, -104.696, 58.344, 58.4976, -5.46455, 1767.41, 7.39264, 0.258123, 2.23795, 577446, 0.01365, 0.878125, 2.0575, 10.2459, 11.3493, 4.28028, 229.101, 254.467

2019-12-07 16:30:00, -53.6606, -37.6592, -12.9649, 16.7411, 10.3359, 0.35345, 9.7646, 3.15273, 2.87101, 33.8227, -87.7701, 58.085, 58.2646, -5.26558, 1753.34, 7.31952, 0.257463, 2.09987, 530969, 0.0133, 0.808875, 1.93655, 10.0528, 11.0972, 4.14625, 236.963, 257.821

2019-12-07 19:30:00, -53.7976, -37.9302, -4.65648, 6.8767, 4.21678, 0.19165, 6.08948, 2.9364, 2.76827, 33.8399, -82.2727, 58.2633, 58.4835, -4.61277, 1762.72, 5.48504, 0.185447, 2.0516, 375486, 0.012525, 0.431075, 2.00468, 10.2339, 10.5817, 2.97342, 265.357, 258.437

2019-12-07 20:30:00, -53.798, -37.9338, -6.11567, 8.49589, 5.2174, 0.180975, 5.7964, 2.98348, 2.76396, 33.8406, -85.5863, 58.2724, 58.4928, -4.90582, 1763.19, 5.499, 0.181962, 2.07388, 378089, 0.012625, 0.414125, 2.03175, 10.2567, 10.5679, 2.93617, 276.55, 260.861

2019-12-08 08:30:00, -53.5495, -38.2502, -6.84462, 15.5532, 9.61089, 0.137075, 4.5148, 3.18065, 2.96715, 33.8645, -49.2247, 57.7995, 58.0436, -2.97863, 1739.41, 6.91513, 0.242389, 2.59845, 625307, 0.01805, 0.765075, 2.48295, 9.60997, 10.1413, 4.08833, 313.518, 274.939

2019-12-08 09:30:00, -53.4754, -38.0956, -5.3124, 9.39679, 5.81661, 0.140125, 4.58595, 3.23528, 3.0073, 33.8636, -64.3943, 57.7295, 57.9571, -3.82297, 1735.83, 6.52417, 0.231228, 2.58767, 586589, 0.0182, 0.7095, 2.48772, 9.54312, 10.0103, 3.90868, 312.972, 277.537

2019-12-08 10:30:00, -53.4163, -37.9719, -6.85703, 7.53832, 4.65084, 0.1563, 5.103, 3.13043, 3.02972, 33.8661, -102.929, 57.7162, 57.9079, -6.14584, 1735.19, 6.07438, 0.21018, 2.56727, 534540, 0.0182, 0.602975, 2.4945, 9.50593, 9.86143, 3.5649, 321.092, 279.191

2019-12-08 11:30:00, -53.379, -37.8833, -5.0518, 5.34418, 3.26743, 0.173975, 5.6133, 2.84337, 2.66887, 33.8564, -113.323, 58.5274, 58.6965, -6.46572, 1776.5, 6.2917, 0.212335, 2.54378, 547506, 0.01815, 0.548875, 2.48365, 9.474, 9.77858, 3.36777, 335.416, 279.567

2019-12-10 11:30:00, -53.988, -37.0434, -9.4622, 9.91307, 6.21063, 0.20525, 6.23508, 3.61915, 3.37742, 33.6304, -109.848, 57.0628, 57.2345, -6.38605, 1698.5, 5.60459, 0.191518, 1.06317, 203680, 0.01265, 0.371875, 0.9933, 7.3318, 8.02942, 2.66958, 214.886, 348.457

2019-12-10 12:30:00, -53.9267, -37.3329, -12.2793, 11.0901, 7.01839, 0.22185, 6.80425, 3.9408, 3.65888, 33.5364, -130.176, 56.446, 56.6698, -7.3371, 1666.03, 5.9002, 0.197901, 1.1616, 233921, 0.013125, 0.453375, 1.06575, 7.51748, 8.45435, 3.05127, 219.482, 317.66

2019-12-10 13:30:00, -53.8655, -37.618, -14.7998, 13.2087, 8.38278, 0.239275, 7.2567, 4.03115, 3.89356, 33.6293, -139.651, 55.9331, 56.1557, -7.36419, 1641.66, 6.3764, 0.217416, 1.42842, 311902, 0.01395, 0.557025, 1.3142, 8.09405, 8.9418, 3.46148, 220.331, 284.13

2019-12-10 15:30:00, -53.8117, -37.9558, -24.315, 23.9573, 14.9499, 0.262175, 7.80245, 3.49245, 3.25092, 33.7933, -121.444, 57.2853, 57.4553, -6.81761, 1712.19, 6.18289, 0.213926, 1.63932, 345498, 0.0146, 0.53455, 1.54822, 8.48235, 9.10275, 3.34075, 217.996, 261.62

2019-12-10 19:30:00, -53.7976, -37.9353, -10.7247, 10.3711, 6.44006, 0.2354, 7.17605, 3.33603, 3.14414, 33.8237, -126.625, 57.505, 57.6746, -6.96795, 1723.8, 6.04181, 0.205274, 1.58825, 324305, 0.013925, 0.4591, 1.51975, 8.54678, 9.05495, 3.07012, 215.295, 260.926

2019-12-10 20:30:00, -53.7949, -37.9295, -11.5527, 11.3688, 7.06652, 0.221, 6.83113, 3.36725, 3.18522, 33.8246, -124.252, 57.4159, 57.5863, -6.83781, 1719.29, 5.93338, 0.202657, 1.57437, 316198, 0.0138, 0.439025, 1.51115, 8.54668, 9.0256, 2.9702, 209.546, 260.935

2019-12-10 21:30:00, -53.7959, -37.9343, -14.3573, 13.8595, 8.62924, 0.217025, 6.72903, 3.42077, 3.23171, 33.8142, -127.284, 57.3227, 57.4901, -6.96103, 1714.4, 5.64922, 0.193423, 1.55755, 296449, 0.0136, 0.397325, 1.5054, 8.55765, 8.96528, 2.80082, 197.906, 260.424

2019-12-11 02:30:00, -53.7538, -38.3486, -10.4108, 12.3069, 7.63337, 0.2472, 7.46048, 3.29948, 3.10076, 33.848, -100.376, 57.6333, 57.7604, -5.71233, 1730.69, 4.09053, 0.13075, 1.58753, 207855, 0.0134, 0.208975, 1.57367, 8.7142, 8.8327, 2.1066, 168.458, 247.824

2019-12-11 05:30:00, -53.7221, -38.5226, -13.3382, 16.5575, 10.2962, 0.262975, 7.82773, 3.38125, 3.28767, 33.8466, -106.194, 57.2225, 57.3613, -5.40337, 1709.8, 4.73744, 0.154081, 1.55735, 239529, 0.0136, 0.2735, 1.53305, 8.57057, 8.76865, 2.42298, 163.827, 244.629

2019-12-11 06:30:00, -53.6237, -38.6949, -12.4547, 20.8859, 12.9328, 0.267525, 7.93168, 3.24615, 3.06643, 33.8588, -73.8588, 57.6958, 57.8311, -4.03017, 1734.04, 4.80714, 0.155938, 1.55757, 243836, 0.013725, 0.291875, 1.52978, 8.52582, 8.7481, 2.49805, 167.065, 242.967

2019-12-11 07:30:00, -53.5249, -38.8689, -15.0427, 19.4936, 12.0975, 0.28115, 8.23198, 3.31688, 3.1235, 33.8578, -85.1851, 57.5683, 57.7087, -5.20578, 1727.53, 5.12716, 0.165624, 1.5453, 259022, 0.01385, 0.32895, 1.5093, 8.45622, 8.7362, 2.6533, 169.359, 242.134

2019-12-11 08:30:00, -53.4227, -39.0469, -18.7491, 20.0095, 12.4116, 0.277525, 8.14562, 3.30125, 3.18949, 33.8532, -119.378, 57.4248, 57.5687, -6.30461, 1720.17, 5.52118, 0.181592, 1.5278, 279176, 0.014, 0.382275, 1.47845, 8.35865, 8.7333, 2.8329, 169.64, 241.755

2019-12-11 09:30:00, -53.3195, -39.2276, -11.3702, 13.123, 8.09839, 0.20685, 6.45202, 3.13848, 2.91993, 33.8387, -98.0832, 58.0011, 58.1536, -5.88254, 1749.3, 5.71813, 0.191736, 1.50802, 289268, 0.01425, 0.427725, 1.44542, 8.24002, 8.70772, 2.95417, 172.263, 240.959

2019-12-11 10:30:00, -53.2145, -39.4106, -12.9019, 13.1546, 8.13237, 0.209275, 6.48842, 3.19493, 3.10721, 33.8389, -123.737, 57.5944, 57.7493, -6.61416, 1728.57, 5.97929, 0.200142, 1.48865, 300949, 0.01455, 0.483025, 1.4071, 8.09912, 8.69207, 3.11825, 175.378, 240.526

2019-12-11 11:30:00, -53.1112, -39.5889, -19.6779, 18.4357, 11.459, 0.25755, 7.69955, 3.36672, 3.14461, 33.878, -127.144, 57.4998, 57.6574, -7.19484, 1724.35, 6.35381, 0.21665, 1.4728, 320379, 0.014975, 0.534525, 1.37165, 7.93708, 8.65365, 3.25115, 175.687, 240.108

2019-12-11 12:30:00, -53.0155, -39.7394, -12.9645, 10.6428, 6.65273, 0.2306, 7.063, 3.54693, 3.37065, 33.89, -159.189, 56.9876, 57.1724, -8.14657, 1698.54, 6.46211, 0.224507, 1.46112, 326220, 0.01545, 0.5646, 1.34712, 7.78185, 8.5716, 3.31088, 174.998, 239.502

2019-12-11 13:30:00, -52.9222, -39.8801, -11.3252, 10.3103, 6.42935, 0.20565, 6.4439, 3.47013, 3.22728, 33.8697, -131.987, 57.2626, 57.4831, -7.37761, 1712.18, 6.23686, 0.219198, 1.4521, 314246, 0.01585, 0.5573, 1.34063, 7.66445, 8.43105, 3.25845, 177.313, 238.045

2019-12-11 14:30:00, -52.8313, -40.0152, -14.9038, 11.1997, 7.00828, 0.24495, 7.37607, 3.58078, 3.39673, 33.9043, -159.663, 56.9041, 57.1131, -8.88876, 1694.53, 5.8819, 0.205387, 1.44655, 292607, 0.016, 0.515, 1.35122, 7.6107, 8.26548, 3.15252, 184.719, 236.662

2019-12-11 18:30:00, -52.8001, -40.092, -20.6449, 14.9311, 9.28985, 0.25125, 7.54873, 3.3982, 3.25563, 33.9164, -174.934, 57.2584, 57.4089, -9.28432, 1712.67, 5.75741, 0.198039, 1.50335, 294548, 0.01585, 0.43135, 1.43937, 7.79143, 8.2344, 2.92025, 213.858, 247.279

2019-12-11 19:30:00, -52.819, -40.1367, -19.6502, 14.9239, 9.25021, 0.26205, 7.80567, 3.27773, 3.12399, 33.9006, -162.72, 57.5638, 57.6949, -8.88177, 1727.95, 5.3337, 0.185838, 1.5376, 278629, 0.01565, 0.381375, 1.4883, 7.94088, 8.29127, 2.71437, 216.149, 252.043

2019-12-11 22:30:00, -52.8087, -40.1138, -8.74095, 6.75248, 4.1825, 0.11935, 3.94647, 3.25595, 3.07827, 33.9075, -161.151, 57.609, 57.7911, -8.73802, 1730.35, 3.45514, 0.117075, 1.64875, 182974, 0.01475, 0.164675, 1.63908, 8.4567, 8.53425, 1.80375, 208.294, 262.087

2019-12-11 23:30:00, -52.8087, -40.1138, -4.26593, 3.31551, 2.05145, 0.093525, 3.06223, 3.22207, 3.03272, 33.9097, -160.03, 57.6927, 57.8885, -8.69628, 1734.65, 2.54733, 0.085012, 1.684, 135971, 0.014525, 0.080575, 1.68158, 8.62115, 8.64353, 1.29565, 216.437, 263.624

2019-12-12 00:30:00, -52.8095, -40.1139, -5.6947, 4.37373, 2.70504, 0.080775, 2.61245, 3.2083, 3.01693, 33.9079, -159.589, 57.7294, 57.9231, -8.80498, 1736.5, 2.08748, 0.0667657, 1.71615, 113081, 0.0143, 0.04155, 1.71558, 8.76842, 8.77585, 1.04435, 241.969, 264.72

2019-12-12 01:30:00, -52.8319, -40.1183, -4.09553, 2.88317, 1.78298, 0.054525, 1.67268, 3.20503, 3.02733, 33.8811, -178.533, 57.7077, 57.9086, -9.60102, 1734.98, 2.17532, 0.066865, 1.74182, 118742, 0.01405, 0.0355, 1.74143, 8.90537, 8.9114, 1.06628, 274.903, 265.201

2019-12-12 04:30:00, -52.8087, -40.1136, -5.62863, 4.15372, 2.57472, 0.08565, 2.78965, 3.2794, 3.08782, 33.9157, -168.507, 57.5812, 57.7681, -9.14427, 1729.06, 3.90843, 0.118153, 1.80773, 220536, 0.0136, 0.12665, 1.80293, 9.22848, 9.27022, 1.79513, 302.623, 263.935

2019-12-12 05:30:00, -52.8087, -40.1136, -13.5288, 9.90654, 6.14619, 0.1347, 4.43192, 3.30797, 3.12812, 33.9176, -171.444, 57.516, 57.681, -9.20667, 1725.77, 4.51713, 0.141607, 1.8241, 261463, 0.013475, 0.1797, 1.81497, 9.3087, 9.38482, 2.01877, 307.463, 263.05

2019-12-12 06:30:00, -52.8086, -40.1138, -9.1779, 6.76105, 4.19534, 0.172125, 5.57682, 3.31305, 3.1452, 33.9183, -169.05, 57.4968, 57.6442, -9.14877, 1724.81, 4.56678, 0.148347, 1.83793, 270151, 0.013375, 0.204225, 1.82648, 9.38145, 9.47697, 2.02965, 315.679, 262.193

2019-12-12 07:30:00, -52.8057, -40.1179, -11.3071, 8.21025, 5.09448, 0.1902, 6.05377, 3.3124, 3.15615, 33.9181, -172.821, 57.4985, 57.6208, -9.28196, 1724.89, 4.47724, 0.144314, 1.84818, 267001, 0.01325, 0.2025, 1.837, 9.45685, 9.55147, 1.98772, 330.721, 261.383

2019-12-12 08:30:00, -52.8067, -40.1157, -9.52787, 7.20002, 4.46036, 0.1651, 5.38612, 3.2606, 3.11382, 33.9145, -167.877, 57.5966, 57.7126, -8.93122, 1729.83, 4.86959, 0.153573, 1.85505, 293357, 0.013125, 0.2159, 1.84225, 9.52425, 9.6316, 2.07008, 347.16, 260.581

2019-12-12 09:30:00, -52.8087, -40.1139, -13.2755, 10.297, 6.37501, 0.2004, 6.33395, 3.241, 3.09425, 33.9095, -161.535, 57.6553, 57.7561, -8.70905, 1732.74, 6.03472, 0.194655, 1.86397, 378132, 0.013125, 0.302275, 1.838, 9.54652, 9.74757, 2.49598, 354.534, 259.476

2019-12-12 15:30:00, -52.84, -40.0906, -12.4088, 8.86432, 5.5057, 0.213875, 6.66857, 3.3434, 3.22121, 33.8685, -173.5, 57.4382, 57.4964, -9.42403, 1721.08, 5.42489, 0.183294, 1.87648, 344722, 0.0133, 0.385675, 1.83628, 9.4964, 9.80068, 2.7279, 323.307, 258.669

2019-12-12 16:30:00, -52.8248, -40.1019, -9.6495, 6.74979, 4.19658, 0.149475, 4.93198, 3.37547, 3.21648, 33.8794, -180.657, 57.4227, 57.5033, -9.6239, 1720.45, 5.51601, 0.188086, 1.86587, 350130, 0.013325, 0.394925, 1.82345, 9.4647, 9.78463, 2.7672, 330.229, 258.282

2019-12-12 17:30:00, -52.8094, -40.1135, -8.62685, 6.34807, 3.96007, 0.159125, 5.20875, 3.4822, 3.33684, 33.892, -171.341, 57.1667, 57.2435, -9.11367, 1707.65, 5.45576, 0.188448, 1.85213, 345493, 0.0134, 0.39265, 1.80985, 9.41498, 9.73453, 2.75535, 336.045, 258.234

2019-12-12 21:30:00, -52.9541, -40.1959, -11.7722, 10.1029, 6.35474, 0.149125, 4.9291, 3.7458, 3.60707, 33.8724, -146.357, 56.5917, 56.6802, -7.74626, 1678.28, 4.62676, 0.147167, 1.73948, 262318, 0.0131, 0.236625, 1.72273, 9.2252, 9.36543, 2.21455, 19.3814, 260.397

2019-12-12 22:30:00, -53.1095, -40.2648, -9.86647, 7.87731, 4.94509, 0.16925, 5.50853, 3.68295, 3.62791, 33.8586, -162.802, 56.5604, 56.6406, -8.31845, 1676.5, 5.24292, 0.169401, 1.70198, 296368, 0.0131, 0.285225, 1.67738, 9.12932, 9.3311, 2.42335, 31.2261, 260.608

2019-12-12 23:30:00, -53.2642, -40.34, -10.9453, 8.92997, 5.55364, 0.198475, 6.2856, 3.38447, 3.18512, 33.8562, -140.71, 57.5151, 57.5772, -8.26385, 1724.8, 6.0392, 0.204468, 1.6685, 349098, 0.0132, 0.337575, 1.63322, 9.00572, 9.2916, 2.5275, 39.1822, 260.87

2019-12-13 00:30:00, -53.4213, -40.4189, -13.665, 10.332, 6.48757, 0.221625, 6.8705, 3.69055, 3.54895, 33.8625, -167.314, 56.7312, 56.8048, -8.81095, 1685.17, 6.62024, 0.236374, 1.64, 393762, 0.013425, 0.40215, 1.58885, 8.85265, 9.26085, 2.63833, 43.1638, 261.116

2019-12-13 04:30:00, -53.811, -40.6139, -6.59625, 13.9063, 8.65173, 0.281675, 8.2664, 3.39642, 3.22289, 33.813, -58.4631, 57.4455, 57.5093, -3.19376, 1720.62, 8.04967, 0.3293, 1.56397, 513177, 0.0163, 0.686825, 1.40387, 7.84272, 8.9588, 3.19498, 60.9096, 264

2019-12-13 05:30:00, -53.978, -40.6998, -10.0325, 20.2917, 12.6622, 0.30855, 8.84993, 3.49167, 3.38416, 33.8052, -49.4117, 57.1001, 57.1688, -3.31053, 1702.98, 8.10316, 0.329881, 1.5332, 505225, 0.016825, 0.7112, 1.35675, 7.64245, 8.84703, 3.2668, 65.007, 265.361

2019-12-13 06:30:00, -54.142, -40.783, -10.2021, 14.3532, 8.88219, 0.2874, 8.39295, 3.2266, 3.10436, 33.7582, -81.779, 57.7198, 57.7794, -4.80272, 1733.74, 8.25528, 0.33815, 1.5029, 510113, 0.0174, 0.726325, 1.31422, 7.44205, 8.70797, 3.29575, 71.0719, 267.408

2019-12-13 08:30:00, -54.464, -40.9479, -3.3251, 6.55548, 4.09518, 0.3216, 9.11158, 3.52672, 3.26749, 33.7649, -63.1237, 57.3545, 57.4284, -3.41148, 1715.28, 8.88378, 0.37426, 1.45765, 551418, 0.019025, 0.776875, 1.2311, 7.00847, 8.4487, 3.37735, 85.2149, 271.941

2019-12-13 11:30:00, -54.9495, -41.1973, -7.09393, 16.7361, 10.6121, 0.47315, 12.086, 4.0029, 3.91537, 33.8673, -51.2415, 55.9634, 56.0423, -2.79037, 1646.6, 9.88565, 0.433286, 1.4972, 651551, 0.024925, 0.987325, 1.12325, 6.20352, 8.1365, 3.68645, 107.848, 277.782

2019-12-13 14:30:00, -55.2585, -41.3586, -27.8203, 41.5847, 25.6691, 0.500225, 12.5741, 3.14655, 3.03336, 33.7663, -80.0172, 57.851, 57.9299, -4.52877, 1740.54, 12.3372, 0.57097, 1.81638, 1.05356e+06, 0.037675, 1.508, 1.00812, 5.55797, 8.05293, 4.44552, 116.729, 282.561

2019-12-13 15:30:00, -55.2585, -41.3586, -33.3892, 48.2401, 29.673, 0.51615, 12.8615, 3.0352, 2.92286, 33.7648, -81.079, 58.0961, 58.1694, -4.70227, 1753.02, 13.1553, 0.633359, 2.02177, 1.29525e+06, 0.043025, 1.7484, 1.01198, 5.48602, 7.88595, 4.70463, 123.179, 299.61

2019-12-13 16:30:00, -55.2586, -41.3586, -32.529, 54.9822, 33.5714, 0.558925, 13.5988, 2.8012, 2.72103, 33.7738, -71.8157, 58.5337, 58.607, -4.04437, 1775.55, 13.6518, 0.671553, 2.23613, 1.50093e+06, 0.046575, 1.97572, 1.0448, 5.54402, 7.699, 4.98275, 131.023, 329.771

2019-12-13 17:30:00, -55.2585, -41.3586, -37.1574, 61.3325, 37.4075, 0.6106, 14.4808, 2.76628, 2.63517, 33.7734, -69.9261, 58.7366, 58.7958, -4.15502, 1785.95, 13.8629, 0.681952, 2.43558, 1.65852e+06, 0.04825, 2.18622, 1.07015, 5.68363, 7.62448, 5.2639, 134.424, 337.866

2019-12-13 18:30:00, -55.2585, -41.3586, -41.9158, 69.478, 42.3761, 0.65345, 15.1865, 2.76668, 2.64348, 33.7728, -71.7796, 58.7265, 58.7777, -4.13688, 1785.42, 14.0297, 0.687027, 2.61438, 1.79316e+06, 0.04885, 2.37258, 1.09465, 5.85297, 7.63177, 5.5245, 134.138, 9.6779

2019-12-13 19:30:00, -55.2668, -41.3484, -32.2298, 55.9332, 34.2922, 0.66465, 15.3573, 2.93098, 2.7259, 33.774, -68.4803, 58.5228, 58.5962, -3.94133, 1774.99, 14.3228, 0.700918, 2.79237, 1.96415e+06, 0.04935, 2.5477, 1.1391, 6.01922, 7.67502, 5.7435, 133.179, 12.6919

2019-12-13 20:30:00, -55.2928, -41.3154, -39.1242, 68.9548, 42.3515, 0.697, 15.8689, 2.98772, 2.8843, 33.7743, -63.6716, 58.1682, 58.2503, -3.85863, 1756.85, 14.7572, 0.724599, 2.9838, 2.16318e+06, 0.0498, 2.728, 1.20432, 6.19445, 7.74552, 5.95167, 133.728, 21.5832

2019-12-13 21:30:00, -55.3194, -41.2802, -32.1535, 52.5883, 32.1305, 0.722575, 16.2626, 2.8217, 2.71096, 33.7703, -72.2604, 58.5427, 58.6301, -4.18075, 1775.95, 15.0351, 0.740369, 3.19592, 2.36521e+06, 0.0499, 2.89565, 1.34675, 6.40247, 7.81095, 6.15975, 140.211, 35.4586

2019-12-13 22:30:00, -55.3449, -41.2471, -38.2086, 58.9608, 35.9116, 0.759825, 16.8362, 2.72283, 2.59536, 33.7707, -76.3402, 58.803, 58.8845, -4.44828, 1789.31, 15.183, 0.753091, 3.43052, 2.58745e+06, 0.0499, 3.07617, 1.51415, 6.63508, 7.99582, 6.36645, 144.997, 43.5145

2019-12-13 23:30:00, -55.3727, -41.2143, -36.0507, 59.566, 36.1642, 0.775275, 17.0727, 2.6215, 2.51259, 33.7798, -71.5533, 58.9918, 59.0648, -4.16547, 1799.16, 15.8697, 0.781825, 3.70257, 2.92601e+06, 0.05015, 3.31513, 1.64502, 6.87503, 8.2915, 6.59035, 144.385, 44.0183

2019-12-14 00:30:00, -55.4082, -41.1742, -41.2761, 81.1514, 49.0324, 0.76395, 16.9024, 2.46918, 2.37611, 33.7914, -58.9279, 59.2951, 59.3651, -3.51573, 1814.96, 16.9593, 0.860017, 4.01527, 3.48746e+06, 0.05115, 3.6184, 1.73575, 7.08915, 8.56425, 6.81935, 142.296, 42.3056

2019-12-14 01:30:00, -55.4429, -41.1255, -26.1393, 51.2154, 30.9218, 0.7752, 17.0723, 2.44572, 2.28304, 33.7938, -60.2649, 59.502, 59.5728, -3.53972, 1825.68, 16.7955, 0.881896, 4.24105, 3.67847e+06, 0.0512, 3.81145, 1.85383, 7.28465, 8.72832, 7.01652, 142.596, 43.0344

2019-12-14 02:30:00, -55.4762, -41.075, -29.2295, 54.5589, 32.9586, 0.74705, 16.6432, 2.46315, 2.3545, 33.7791, -62.6775, 59.3395, 59.4172, -3.70555, 1817.06, 16.0481, 0.818774, 4.32713, 3.5136e+06, 0.050025, 3.80807, 2.04675, 7.44283, 8.84903, 7.10735, 144.964, 48.5749

2019-12-14 03:30:00, -55.5129, -41.0205, -28.3827, 59.7322, 35.8712, 0.7357, 16.4711, 2.27668, 2.18464, 33.7646, -57.4433, 59.7284, 59.8034, -3.30417, 1836.92, 16.5469, 0.819894, 4.42793, 3.64679e+06, 0.049725, 3.87467, 2.13842, 7.55112, 8.973, 7.19132, 146.958, 50.8192

2019-12-14 04:30:00, -55.5494, -40.9571, -25.1386, 72.6461, 43.5607, 0.7239, 16.2899, 2.22918, 2.0628, 33.7669, -36.9615, 60.0045, 60.0786, -2.41679, 1851.25, 16.804, 0.843237, 4.5526, 3.81774e+06, 0.049975, 3.99672, 2.17508, 7.6379, 9.01853, 7.30393, 150.081, 51.311

2019-12-14 05:30:00, -55.5821, -40.8873, -32.8037, 75.6408, 45.5763, 0.7149, 16.1488, 2.38172, 2.25491, 33.7704, -45.0323, 59.5695, 59.6432, -3.00965, 1828.8, 16.6145, 0.831409, 4.6104, 3.81473e+06, 0.04975, 4.00715, 2.27477, 7.70497, 9.05225, 7.34527, 153.62, 55.4125

2019-12-14 06:30:00, -55.609, -40.817, -28.0187, 59.1298, 35.5647, 0.684825, 15.6795, 2.32573, 2.20653, 33.766, -55.9203, 59.6834, 59.7535, -3.29355, 1834.61, 16.7173, 0.831608, 4.62295, 3.84131e+06, 0.049525, 4.0016, 2.31055, 7.7335, 9.06663, 7.36305, 156.648, 57.7111

2019-12-14 07:30:00, -55.6314, -40.7521, -23.8176, 56.4799, 33.9545, 0.66795, 15.4141, 2.31053, 2.17635, 33.7736, -49.8483, 59.7579, 59.8193, -2.93459, 1838.59, 16.7907, 0.840754, 4.6168, 3.86278e+06, 0.049475, 4.0088, 2.28535, 7.73283, 9.03315, 7.38255, 160.306, 57.9958

2019-12-14 08:30:00, -55.6499, -40.6937, -25.4479, 56.7532, 34.1092, 0.688925, 15.7478, 2.30163, 2.17547, 33.7743, -50.1951, 59.764, 59.8211, -3.12052, 1838.92, 16.59, 0.829853, 4.58058, 3.76695e+06, 0.049175, 3.95192, 2.31102, 7.72505, 9.00877, 7.35698, 164.852, 61.3845

2019-12-14 09:30:00, -55.6741, -40.6526, -29.7283, 64.0208, 38.4281, 0.685375, 15.6923, 2.26153, 2.13653, 33.7707, -51.4671, 59.8531, 59.9102, -3.23556, 1843.47, 16.0482, 0.792314, 4.5045, 3.52336e+06, 0.048375, 3.8402, 2.34972, 7.72255, 8.97942, 7.32035, 169.642, 66.0102

2019-12-14 10:30:00, -55.6946, -40.6209, -24.5374, 50.4086, 30.2675, 0.6762, 15.5475, 2.27197, 2.13276, 33.7875, -56.8484, 59.8516, 59.9135, -3.39217, 1843.66, 15.6786, 0.754722, 4.4279, 3.3201e+06, 0.047525, 3.7463, 2.35655, 7.72317, 8.94935, 7.304, 173.788, 69.1813

2019-12-14 12:30:00, -55.5796, -40.7961, -45.3787, 77.0654, 46.2058, 0.80285, 17.4866, 2.22585, 2.10506, 33.7668, -70.3402, 59.9066, 59.9827, -4.10562, 1846.18, 15.8493, 0.759821, 4.45862, 3.37423e+06, 0.047075, 3.79503, 2.33655, 7.78925, 9.03255, 7.38223, 180.29, 73.0958

2019-12-14 13:30:00, -55.4446, -41.0013, -32.2039, 53.3849, 31.851, 0.821125, 17.7562, 2.0712, 1.91277, 33.7671, -62.8316, 60.3445, 60.4208, -4.23289, 1868.91, 15.7553, 0.757942, 4.51178, 3.39715e+06, 0.0469, 3.85883, 2.33385, 7.85085, 9.09355, 7.45983, 183.322, 75.1111

2019-12-14 14:30:00, -55.3093, -41.1944, -36.1778, 57.3857, 34.4844, 0.811775, 17.613, 2.29715, 2.11732, 33.7671, -74.9933, 59.8606, 59.9548, -4.39421, 1843.8, 15.5058, 0.743177, 4.52287, 3.33182e+06, 0.0464, 3.87975, 2.32078, 7.9001, 9.08062, 7.53973, 186.309, 77.4106

2019-12-14 15:30:00, -55.1726, -41.3699, -35.8829, 104.787, 63.2091, 0.764525, 16.906, 2.4171, 2.1508, 33.7657, -60.2431, 59.7699, 59.8795, -2.385, 1839.08, 15.2524, 0.722228, 4.50323, 3.22581e+06, 0.045875, 3.84407, 2.3414, 7.92952, 9.11637, 7.54963, 188.943, 83.975

2019-12-14 16:30:00, -55.0385, -41.5361, -24.9543, 76.4521, 47.0095, 0.7323, 16.4088, 3.0237, 2.89318, 33.8471, -33.8722, 58.0741, 58.2091, -2.2175, 1753.16, 14.9494, 0.698811, 4.45265, 3.08417e+06, 0.045225, 3.77617, 2.35548, 7.94172, 9.10855, 7.549, 191.767, 91.5139

2019-12-14 20:30:00, -55.0307, -41.6157, -9.1577, 33.3207, 20.5379, 0.577825, 13.9113, 3.1, 2.96175, 33.8787, -27.3198, 57.8902, 58.051, -1.86237, 1744.25, 13.6815, 0.588156, 3.97777, 2.32144e+06, 0.04185, 3.15535, 2.41823, 7.80163, 9.02105, 7.13997, 197.133, 114.929

2019-12-14 21:30:00, -55.0634, -41.5854, -12.7292, 47.39, 29.0363, 0.56095, 13.6251, 2.91115, 2.76182, 33.8621, -27.1638, 58.3284, 58.4909, -1.83182, 1766.39, 13.3253, 0.573183, 3.87743, 2.20973e+06, 0.04135, 3.01992, 2.429, 7.7499, 9.00005, 6.9987, 197.71, 118.687

2019-12-14 22:30:00, -55.0971, -41.5576, -18.9623, 60.4806, 36.6822, 0.525075, 13.0029, 2.58942, 2.56013, 33.8225, -27.6617, 58.7987, 58.9466, -2.15081, 1789.9, 13.261, 0.571384, 3.79335, 2.16129e+06, 0.041025, 2.93335, 2.40282, 7.69523, 8.979, 6.89285, 199.574, 120.135

2019-12-14 23:30:00, -55.1402, -41.5327, -21.2843, 45.2393, 26.8847, 0.4953, 12.4758, 1.94695, 1.8215, 33.7366, -49.2198, 60.5094, 60.64, -3.30734, 1876.98, 13.0547, 0.563865, 3.71995, 2.07208e+06, 0.04075, 2.84865, 2.38963, 7.64535, 8.9327, 6.79645, 202.588, 121.56

2019-12-15 00:30:00, -55.1816, -41.5135, -22.9362, 45.7458, 27.2723, 0.458225, 11.7994, 2.04695, 1.80162, 33.7364, -58.193, 60.5486, 60.6859, -3.52909, 1879.02, 12.5027, 0.530507, 3.63765, 1.90305e+06, 0.040175, 2.69775, 2.43663, 7.61585, 8.8609, 6.65433, 206.155, 125.593

2019-12-15 01:30:00, -55.2216, -41.503, -12.7587, 23.6244, 14.1952, 0.4401, 11.4587, 2.29425, 2.13484, 33.7639, -61.3569, 59.7607, 59.9161, -3.75795, 1838.58, 12.1793, 0.502808, 3.54573, 1.76828e+06, 0.0394, 2.54885, 2.46265, 7.59147, 8.79202, 6.52578, 210.622, 128.86

2019-12-15 02:30:00, -55.2631, -41.4977, -11.8897, 23.4669, 14.0742, 0.45535, 11.735, 2.23548, 2.06617, 33.7773, -58.267, 59.9101, 60.0677, -3.53331, 1846.53, 11.9586, 0.487285, 3.45545, 1.67031e+06, 0.038725, 2.44198, 2.4431, 7.5575, 8.7108, 6.45708, 216.051, 130.085

2019-12-15 03:30:00, -55.308, -41.4939, -18.6873, 38.0154, 22.7791, 0.468025, 11.9738, 2.20697, 2.03933, 33.7786, -54.6019, 59.9711, 60.1283, -3.43103, 1849.71, 11.7092, 0.475866, 3.36445, 1.59239e+06, 0.0382, 2.3656, 2.39105, 7.51278, 8.63143, 6.4212, 220.919, 130.035

2019-12-15 04:30:00, -55.3531, -41.502, -17.9138, 35.3143, 21.1327, 0.460675, 11.8405, 2.1654, 2.01947, 33.7818, -58.3098, 60.0152, 60.1725, -3.54235, 1852.05, 11.5405, 0.470753, 3.27185, 1.53336e+06, 0.03765, 2.31743, 2.30843, 7.4596, 8.58352, 6.39215, 223.043, 128.852

2019-12-15 05:30:00, -55.3957, -41.5201, -20.3393, 40.6582, 24.2886, 0.44195, 11.4923, 2.11102, 1.95178, 33.7914, -57.2055, 60.1683, 60.324, -3.50129, 1860.15, 11.459, 0.4674, 3.18295, 1.47901e+06, 0.0372, 2.27252, 2.22735, 7.40338, 8.54665, 6.34943, 223.074, 127.86

2019-12-15 06:30:00, -55.4378, -41.5413, -19.8219, 39.6283, 23.6744, 0.44205, 11.4948, 2.11238, 1.91289, 33.7945, -58.3963, 60.2717, 60.412, -3.50704, 1865.57, 11.2978, 0.458739, 3.09983, 1.40979e+06, 0.036775, 2.21173, 2.17055, 7.34663, 8.50273, 6.27547, 223.576, 128.336

2019-12-15 08:30:00, -55.3005, -41.9686, -11.5654, 23.1866, 13.9207, 0.3828, 10.3453, 2.26852, 2.053, 33.7828, -57.7024, 59.9161, 60.0959, -3.47969, 1846.93, 10.566, 0.416037, 2.91812, 1.19958e+06, 0.03525, 2.0551, 2.07032, 7.2827, 8.34185, 6.24845, 229.452, 132.015

2019-12-15 09:30:00, -55.226, -42.1391, -10.8027, 21.8142, 13.105, 0.400525, 10.6972, 2.28828, 2.11908, 33.7869, -56.5232, 59.7747, 59.9447, -3.44698, 1839.67, 10.3637, 0.399903, 2.83392, 1.1277e+06, 0.03455, 1.97545, 2.0309, 7.24648, 8.29387, 6.17955, 229.393, 134.289

2019-12-15 10:30:00, -55.182, -42.2392, -14.9593, 28.3864, 17.0179, 0.38175, 10.3393, 2.22283, 2.10411, 33.7686, -60.1276, 59.8486, 59.9843, -3.67081, 1843.2, 10.3103, 0.396324, 2.76202, 1.08614e+06, 0.034125, 1.93792, 1.96665, 7.19648, 8.25015, 6.14825, 230.102, 134.433

2019-12-15 11:30:00, -55.1435, -42.364, -14.0865, 24.6465, 14.7552, 0.309675, 8.82185, 2.17905, 2.00225, 33.7424, -66.4998, 60.0906, 60.224, -3.99575, 1855.33, 9.9453, 0.377855, 2.68357, 1.00042e+06, 0.0336, 1.8501, 1.9424, 7.1485, 8.1537, 6.0768, 234.106, 137.974

2019-12-15 12:30:00, -55.0999, -42.4922, -11.8851, 22.6745, 13.5493, 0.299725, 8.64288, 2.12005, 1.97926, 33.7298, -63.6729, 60.1493, 60.2804, -3.66649, 1858.17, 9.79773, 0.364586, 2.60147, 943895, 0.033, 1.76133, 1.91345, 7.1058, 8.07055, 6.00387, 238.576, 141.285

2019-12-15 13:30:00, -55.0562, -42.6202, -10.5657, 21.9847, 13.1223, 0.30195, 8.71118, 2.0845, 1.92436, 33.7176, -55.0848, 60.2863, 60.4097, -3.36848, 1865.08, 9.34314, 0.352362, 2.51365, 868338, 0.032225, 1.621, 1.917, 7.0684, 7.9511, 5.8591, 242.561, 147.602

2019-12-15 15:30:00, -55.0471, -42.7561, -12.5501, 21.757, 13.0358, 0.234225, 7.17073, 2.20412, 2.03896, 33.7387, -66.9786, 60.0147, 60.1416, -4.02812, 1851.33, 7.7452, 0.284587, 2.31335, 649416, 0.0298, 1.03845, 2.0632, 7.0499, 7.66377, 4.63433, 258.107, 174.707

2019-12-15 16:30:00, -55.0471, -42.7561, -8.5747, 14.5726, 8.75585, 0.178175, 5.753, 2.29285, 2.11166, 33.7422, -68.0903, 59.838, 59.9754, -4.09915, 1842.23, 7.29653, 0.26767, 2.2238, 586333, 0.028675, 0.85085, 2.05128, 7.04683, 7.5585, 4.0894, 268.328, 180.738

2019-12-15 17:30:00, -55.0471, -42.7561, -6.7985, 11.5594, 6.96409, 0.19795, 6.27363, 2.37767, 2.20936, 33.7447, -68.4578, 59.6253, 59.7537, -4.08477, 1831.27, 6.63384, 0.243773, 2.13635, 508803, 0.0275, 0.6727, 2.02515, 7.0504, 7.44538, 3.52228, 277.648, 185.894

2019-12-15 18:30:00, -55.044, -42.7643, -6.73463, 11.4922, 6.93491, 0.1823, 5.86078, 2.42925, 2.26972, 33.7452, -68.6237, 59.5083, 59.6177, -4.06331, 1825.23, 6.06376, 0.217228, 2.05133, 440782, 0.026325, 0.549875, 1.9756, 7.06168, 7.3701, 3.16565, 289.315, 188.708

2019-12-15 19:30:00, -55.0302, -42.7935, -6.2433, 10.1201, 6.11809, 0.182, 5.86285, 2.48693, 2.33726, 33.7502, -72.1423, 59.3682, 59.4646, -4.26907, 1818.08, 6.25385, 0.216906, 1.97378, 433909, 0.025375, 0.5448, 1.89643, 7.05842, 7.3796, 3.24778, 303.007, 188.131

2019-12-15 20:30:00, -55.0163, -42.8238, -6.59, 10.1769, 6.15641, 0.213525, 6.67643, 2.5075, 2.36325, 33.7531, -76.6438, 59.3169, 59.4056, -4.47774, 1815.49, 6.88619, 0.241945, 1.91153, 468747, 0.02485, 0.600825, 1.81385, 7.01577, 7.41308, 3.45173, 314.531, 186.747

2019-12-15 23:30:00, -55.0249, -42.7757, -9.21232, 14.3863, 8.68163, 0.2938, 8.5348, 2.43032, 2.31986, 33.7509, -77.3182, 59.4436, 59.5034, -4.43536, 1821.99, 8.07978, 0.307703, 1.8001, 566574, 0.025125, 0.727025, 1.64445, 6.77687, 7.41338, 3.54735, 343.304, 189.043

2019-12-16 00:30:00, -54.9981, -42.8001, -11.4589, 20.4116, 12.2743, 0.317575, 9.05232, 2.31893, 2.19782, 33.7346, -67.7423, 59.7396, 59.7829, -3.90488, 1837.02, 8.76769, 0.352938, 1.7962, 641401, 0.02625, 0.861275, 1.57392, 6.62312, 7.48345, 3.76193, 345.155, 187.301

2019-12-16 02:30:00, -54.9825, -42.7636, -10.9316, 22.3824, 13.4259, 0.338225, 9.48428, 2.2404, 2.15118, 33.7227, -57.6313, 59.8663, 59.8919, -3.40256, 1843.38, 9.92535, 0.419188, 1.84175, 787420, 0.029675, 1.1058, 1.46888, 6.30828, 7.56377, 4.09005, 350.14, 188.998

2019-12-16 03:30:00, -54.9454, -42.7225, -10.5103, 23.5712, 14.1984, 0.388025, 10.486, 2.37242, 2.19204, 33.7394, -53.9671, 59.7814, 59.7944, -3.10474, 1839.26, 10.6909, 0.470094, 1.91655, 905468, 0.0326, 1.30052, 1.40287, 6.13603, 7.66275, 4.3589, 347.333, 185.753

2019-12-16 04:30:00, -54.9215, -42.692, -8.80357, 20.6648, 12.5499, 0.406925, 10.8586, 2.63095, 2.5182, 33.7719, -49.5735, 59.0417, 59.0549, -2.93459, 1801.6, 10.7256, 0.476999, 1.9949, 939664, 0.034875, 1.46735, 1.34872, 6.05175, 7.74617, 4.63075, 342.237, 182.429

2019-12-16 05:30:00, -54.9196, -42.6893, -12.652, 31.1962, 18.9635, 0.38875, 10.5037, 2.66057, 2.55289, 33.7771, -47.5764, 58.9672, 58.9764, -2.79072, 1797.85, 10.3915, 0.451626, 2.04713, 912939, 0.03565, 1.55565, 1.3296, 6.06292, 7.74532, 4.85585, 339.451, 185.686

2019-12-16 06:30:00, -54.9207, -42.6915, -9.71413, 23.7965, 14.4656, 0.349975, 9.7204, 2.66092, 2.57037, 33.7784, -46.2427, 58.9299, 58.9374, -2.80722, 1795.95, 10.2062, 0.427916, 2.08283, 882081, 0.035675, 1.60488, 1.32635, 6.1153, 7.71413, 5.04695, 339.284, 193.967

2019-12-16 08:30:00, -54.8963, -43.1476, -6.96995, 13.6467, 8.21204, 0.357525, 9.87692, 2.34095, 2.25747, 33.7473, -58.4225, 59.6525, 59.6446, -3.54806, 1832.72, 9.67605, 0.389462, 2.13955, 815601, 0.03475, 1.65705, 1.34842, 6.28045, 7.62205, 5.4243, 332.057, 219.267

2019-12-16 09:30:00, -54.8633, -43.462, -8.28285, 16.5171, 9.93956, 0.31355, 8.96355, 2.34173, 2.22442, 33.7761, -56.7284, 59.7163, 59.7101, -3.48791, 1836.48, 8.58363, 0.335979, 2.1315, 692870, 0.03315, 1.36658, 1.61115, 6.4164, 7.36035, 5.08915, 328.77, 292.335

2019-12-16 10:30:00, -54.828, -43.7767, -7.43645, 13.9011, 8.35829, 0.261325, 7.81297, 2.3153, 2.25093, 33.7802, -64.8021, 59.6531, 59.6492, -3.71637, 1833.27, 7.78984, 0.290795, 2.10042, 601186, 0.0314, 1.01218, 1.8352, 6.5467, 7.21163, 4.37555, 323.353, 325.13

2019-12-16 11:30:00, -54.7933, -44.0985, -6.77388, 16.6983, 10.0729, 0.20675, 6.49035, 2.418, 2.13537, 33.7882, -49.8173, 59.9179, 59.9074, -2.8325, 1847.11, 7.31306, 0.269, 2.06547, 549656, 0.03, 0.8262, 1.89148, 6.64297, 7.17977, 3.89355, 314.836, 327.181

2019-12-16 12:30:00, -54.7509, -44.4201, -7.86385, 24.2382, 15.2573, 0.2396, 7.28615, 3.76975, 3.36823, 33.8987, -37.7581, 57.1535, 57.175, -2.17887, 1707.08, 7.16099, 0.259316, 2.0372, 528430, 0.028975, 0.760875, 1.88922, 6.70862, 7.20788, 3.71725, 310.015, 325.632

2019-12-16 13:30:00, -54.7068, -44.7419, -7.47478, 22.3645, 14.452, 0.28985, 8.45322, 4.61065, 4.44419, 33.9932, -41.296, 54.8944, 54.934, -2.16661, 1595.03, 7.65761, 0.273587, 2.02045, 560132, 0.028475, 0.86235, 1.82427, 6.73955, 7.34488, 4.08012, 308.672, 324.616

2019-12-16 14:30:00, -54.6717, -45.0764, -4.39407, 15.7672, 10.206, 0.281275, 8.2605, 4.66493, 4.52284, 34.0048, -28.2051, 54.7329, 54.7739, -1.80187, 1587.17, 8.23765, 0.29744, 2.01317, 600596, 0.0284, 1.04367, 1.7179, 6.74097, 7.4977, 4.708, 309.527, 324.296

2019-12-16 15:30:00, -54.6373, -45.4047, -4.69703, 12.5968, 8.14535, 0.3234, 9.17265, 4.63172, 4.6258, 33.9851, -42.7784, 54.5422, 54.575, -2.40178, 1577.45, 8.53727, 0.308647, 2.0048, 619835, 0.028325, 1.15723, 1.6353, 6.73097, 7.5911, 5.04598, 313.961, 319.855

2019-12-16 16:30:00, -54.5956, -45.7174, -6.68503, 18.3743, 11.7986, 0.348625, 9.6973, 4.40705, 4.27813, 33.9494, -39.522, 55.255, 55.2798, -2.37089, 1612.36, 8.78285, 0.31842, 1.99415, 636168, 0.0284, 1.23408, 1.56498, 6.70502, 7.6523, 5.2171, 317.823, 313.745

2019-12-16 18:30:00, -54.5206, -45.9085, -4.32867, 15.4709, 10.088, 0.3626, 9.969, 4.90177, 4.79292, 34.017, -38.0799, 54.1712, 54.2367, -1.7928, 1559.55, 8.49517, 0.317718, 1.99008, 621584, 0.028775, 1.11187, 1.64482, 6.65375, 7.56767, 4.65982, 309.028, 320.671

2019-12-16 23:30:00, -54.5615, -45.9347, -5.29558, 14.0517, 9.12881, 0.16095, 5.22897, 4.78265, 4.60444, 34.0185, -41.1396, 54.4869, 54.608, -2.42742, 1575.17, 4.9979, 0.177271, 1.84605, 318619, 0.0243, 0.355275, 1.81053, 6.97335, 7.15498, 2.51753, 297.382, 315.447

2019-12-17 00:30:00, -54.5574, -45.9694, -3.67427, 9.31535, 6.06749, 0.1329, 4.39845, 4.86625, 4.67777, 34.0224, -44.0398, 54.3112, 54.4618, -2.53355, 1566.54, 4.57452, 0.156572, 1.815, 280003, 0.023425, 0.27615, 1.79363, 7.0448, 7.16653, 2.23442, 304.97, 315.029

2019-12-17 01:30:00, -54.5543, -46.0059, -3.63407, 8.97949, 5.86012, 0.1305, 4.3183, 4.92908, 4.74067, 34.0234, -44.4478, 54.1828, 54.3376, -2.59426, 1560.21, 4.37932, 0.147081, 1.78773, 259859, 0.02265, 0.2378, 1.77172, 7.10708, 7.20412, 2.09793, 311.173, 315.108

2019-12-17 08:30:00, -54.4418, -46.3505, -0.42585, 1.3403, 0.866331, 0.140825, 4.66793, 4.6193, 4.47985, 33.9815, -37.2307, 54.7611, 54.8661, -2.05467, 1588.25, 3.66433, 0.11542, 1.6629, 194202, 0.0193, 0.152525, 1.65575, 7.4293, 7.48178, 1.8092, 283.453, 309.969

2019-12-17 10:30:00, -54.3231, -46.972, -2.92223, 10.0867, 6.53416, 0.155125, 5.10882, 4.6904, 4.5611, 33.9848, -31.1822, 54.6212, 54.7034, -1.86942, 1581.36, 4.93456, 0.161758, 1.68898, 275443, 0.0189, 0.285, 1.66475, 7.56125, 7.717, 2.40405, 300.414, 300.553

2019-12-17 11:30:00, -54.2691, -47.2944, -4.6176, 13.0536, 8.434, 0.167275, 5.4214, 4.60597, 4.4863, 33.9572, -38.3416, 54.759, 54.8601, -2.28701, 1587.8, 4.90857, 0.163141, 1.72872, 281687, 0.018825, 0.279425, 1.70595, 7.6671, 7.8154, 2.31825, 294.928, 294.495

2019-12-17 12:30:00, -54.2159, -47.6191, -8.50215, 22.4519, 14.4887, 0.228725, 7.0254, 4.56682, 4.40085, 33.9466, -42.164, 54.9105, 55.0339, -2.45442, 1595.18, 5.58101, 0.176544, 1.79022, 333152, 0.019075, 0.357425, 1.75217, 7.7533, 7.97002, 2.72155, 262.924, 288.38

2019-12-17 13:30:00, -54.1659, -47.9479, -7.44262, 27.275, 17.5425, 0.3039, 8.71235, 4.45947, 4.32338, 33.9075, -32.3026, 55.0888, 55.2006, -1.77301, 1603.49, 7.23882, 0.238718, 1.90217, 478420, 0.020625, 0.613275, 1.7948, 7.68915, 8.16525, 3.618, 232.871, 281.428

2019-12-17 18:30:00, -53.8956, -49.2649, -16.3721, 43.9054, 28.0626, 0.442825, 11.5093, 4.25858, 4.08939, 33.8894, -41.9303, 55.5347, 55.6796, -2.4397, 1625.46, 11.4556, 0.451132, 3.03158, 1.35608e+06, 0.035125, 2.5153, 1.69035, 7.43163, 8.48003, 7.00425, 204.89, 265.275

2019-12-17 19:30:00, -53.9175, -49.2881, -18.3061, 49.2009, 31.4487, 0.449125, 11.6307, 4.26, 4.09228, 33.8895, -41.8849, 55.5334, 55.6737, -2.43425, 1625.4, 11.1366, 0.436048, 3.0405, 1.31007e+06, 0.035075, 2.49953, 1.7289, 7.4503, 8.396, 7.0468, 206.307, 253.548

2019-12-17 20:30:00, -53.9419, -49.3126, -15.6903, 40.9525, 26.1657, 0.426575, 11.1952, 4.24693, 4.08104, 33.8891, -42.1008, 55.5443, 55.6967, -2.50694, 1625.94, 10.5866, 0.409527, 2.99115, 1.20204e+06, 0.03455, 2.38645, 1.79675, 7.44707, 8.27382, 7.02192, 207.557, 240.007

2019-12-17 21:30:00, -53.9558, -49.3329, -14.3685, 35.1013, 22.408, 0.367, 10.034, 4.21945, 4.01507, 33.8835, -46.3194, 55.6549, 55.8331, -2.68335, 1631.39, 9.74145, 0.368443, 2.90053, 1.04777e+06, 0.033675, 2.09045, 1.99192, 7.4302, 8.20235, 6.73412, 210.158, 222.707

2019-12-17 23:30:00, -53.9171, -49.2837, -8.71487, 22.3812, 14.2923, 0.295275, 8.53357, 4.22963, 4.02745, 33.8826, -43.0459, 55.5999, 55.808, -2.55012, 1628.63, 8.65886, 0.317709, 2.69815, 843364, 0.03175, 1.44975, 2.26397, 7.37415, 8.10383, 5.55857, 216.227, 209.402

2019-12-18 00:30:00, -53.9437, -49.3104, -12.4461, 29.7368, 18.9676, 0.2646, 7.85095, 4.19277, 3.99887, 33.8795, -45.9779, 55.6471, 55.8674, -2.74281, 1630.94, 8.02545, 0.29391, 2.57915, 747721, 0.03085, 1.14602, 2.3071, 7.3161, 7.94987, 4.82088, 219.027, 207.656

2019-12-18 01:30:00, -53.9719, -49.3368, -9.27603, 20.2702, 12.9113, 0.257375, 7.68783, 4.148, 3.94682, 33.8695, -52.0351, 55.7615, 55.977, -3.00418, 1636.52, 7.55495, 0.276748, 2.46413, 673232, 0.02995, 0.973, 2.26282, 7.25812, 7.80923, 4.3862, 222.93, 206.768

2019-12-18 03:30:00, -53.982, -49.3929, -9.74622, 23.9309, 15.271, 0.2657, 7.87365, 4.20682, 3.98937, 33.8751, -47.2412, 55.6714, 55.8881, -2.67024, 1632.09, 6.44042, 0.23513, 2.2638, 519199, 0.0281, 0.704825, 2.14958, 7.18417, 7.5736, 3.69945, 229.063, 207.234

2019-12-18 04:30:00, -53.9921, -49.4372, -10.0009, 26.605, 16.9941, 0.212275, 6.60615, 4.23818, 4.02409, 33.8803, -42.4514, 55.5765, 55.8155, -2.4609, 1627.42, 5.48221, 0.199989, 2.16988, 416535, 0.027025, 0.5122, 2.10512, 7.17188, 7.4269, 3.10423, 229.004, 208.444

2019-12-18 08:30:00, -53.8759, -49.868, -2.139, 3.79963, 2.52607, 0.085875, 2.73878, 5.52985, 5.15979, 33.9999, -67.8096, 53.1578, 53.5283, -3.55101, 1509.59, 3.71836, 0.109871, 1.86263, 217744, 0.022525, 0.138625, 1.85702, 7.27663, 7.3158, 1.81263, 309.707, 208.769

2019-12-18 09:30:00, -53.7796, -50.1593, -5.75157, 10.3845, 6.96045, 0.151075, 4.8417, 5.79573, 5.4976, 34.0191, -64.3627, 52.5496, 52.8784, -3.45846, 1480.25, 4.89243, 0.148894, 1.79355, 281389, 0.021525, 0.18485, 1.78368, 7.30745, 7.37175, 1.94207, 31.5515, 207.642

2019-12-18 10:30:00, -53.7023, -50.4449, -7.0576, 14.0894, 9.42985, 0.26645, 7.86045, 5.74782, 5.56285, 34.0183, -63.0193, 52.5008, 52.7554, -3.12404, 1477.87, 6.79233, 0.219683, 1.7319, 408910, 0.020875, 0.289425, 1.70495, 7.2898, 7.4483, 2.25625, 23.2692, 206.305

2019-12-18 11:30:00, -53.6431, -50.6978, -10.9657, 22.2703, 14.8954, 0.3742, 10.1704, 5.7264, 5.46526, 34.021, -53.2815, 52.7278, 52.9391, -3.084, 1488.93, 8.79521, 0.332005, 1.7096, 608032, 0.0218, 0.544075, 1.61137, 7.08713, 7.58047, 2.92902, 30.5266, 204.51

2019-12-18 12:30:00, -53.5814, -50.9404, -14.2146, 26.741, 17.8613, 0.468075, 11.977, 5.68197, 5.56013, 34.012, -62.2813, 52.5873, 52.7622, -3.3194, 1481.98, 10.5208, 0.465301, 1.7678, 841667, 0.025575, 0.901175, 1.5097, 6.66235, 7.74173, 3.60305, 31.2843, 202.902

2019-12-18 13:30:00, -53.5198, -51.1777, -16.5881, 32.2814, 21.4313, 0.551375, 13.4735, 5.48438, 5.26703, 34.0079, -59.791, 53.1776, 53.3202, -3.2417, 1510.66, 11.3827, 0.521899, 1.90045, 1.00195e+06, 0.030975, 1.2521, 1.42078, 6.27685, 7.8456, 4.19632, 31.9255, 203.957

2019-12-18 14:30:00, -53.4615, -51.4238, -18.8055, 38.3691, 25.6046, 0.61025, 14.4716, 5.65217, 5.47234, 34.02, -56.1818, 52.7994, 52.9259, -3.07269, 1492.39, 12.3448, 0.573467, 2.0836, 1.2097e+06, 0.036275, 1.60408, 1.31997, 6.06917, 7.74263, 4.88363, 34.0633, 207.118

2019-12-18 15:30:00, -53.4118, -51.6791, -20.1919, 37.3925, 25.0877, 0.640875, 14.9784, 5.8277, 5.60142, 34.0165, -63.0335, 52.5758, 52.6832, -3.37393, 1481.48, 12.9507, 0.611232, 2.27773, 1.3936e+06, 0.040175, 1.9002, 1.25205, 6.0262, 7.6638, 5.33845, 37.242, 217.367

2019-12-18 16:30:00, -53.3643, -51.9304, -23.8818, 49.2249, 33.2459, 0.651275, 15.1448, 6.0442, 5.89459, 34.0213, -59.4075, 52.0394, 52.1346, -3.00357, 1455.61, 13.1935, 0.623688, 2.44395, 1.51141e+06, 0.04215, 2.12878, 1.19578, 6.0951, 7.64175, 5.63505, 38.4931, 228.153

2019-12-18 21:30:00, -53.2364, -52.0114, -13.6939, 51.6121, 34.8227, 0.5972, 14.2218, 6.01088, 5.89303, 34.0294, -28.2009, 52.1013, 52.1353, -1.64434, 1458.7, 11.3238, 0.491013, 2.5264, 1.21479e+06, 0.039225, 2.16385, 1.3008, 6.4229, 8.24458, 5.8124, 45.105, 13.1079

2019-12-18 22:30:00, -53.2166, -51.9666, -15.3095, 55.029, 37.1149, 0.7875, 17.2694, 5.99927, 5.88567, 34.0294, -30.3977, 52.1236, 52.149, -1.72483, 1459.77, 10.3859, 0.437651, 2.49995, 1.06409e+06, 0.038025, 2.06533, 1.39468, 6.48778, 8.04802, 5.82332, 38.6907, 30.2439

2019-12-19 08:30:00, -53.1437, -52.7661, -5.8676, 21.993, 14.8243, 0.425, 11.2008, 5.97927, 5.90483, 34.0041, -26.9365, 52.1195, 52.1202, -1.65355, 1459.26, 12.6093, 0.569938, 2.81305, 1.58918e+06, 0.04165, 2.23572, 1.70507, 6.57773, 7.9677, 5.81027, 292.165, 31.4694

2019-12-19 09:30:00, -53.0957, -53.0498, -10.9886, 38.1781, 25.6811, 0.385325, 10.4346, 5.91215, 5.82071, 33.9952, -31.5455, 52.2725, 52.2787, -1.78845, 1466.53, 12.0034, 0.530176, 2.91955, 1.51381e+06, 0.040825, 2.3143, 1.7776, 6.76957, 8.1019, 6.03092, 294.167, 20.3177

2019-12-19 10:30:00, -53.0323, -53.3231, 0.27025, -0.952807, -0.64071, 0.4003, 10.7292, 5.90145, 5.76336, 33.9836, -29.1499, 52.3698, 52.3887, -1.76553, 1471.08, 11.784, 0.494421, 2.97915, 1.46064e+06, 0.039575, 2.30228, 1.88702, 6.94575, 8.20195, 6.1463, 290.195, 24.0016

2019-12-19 11:30:00, -52.963, -53.5909, -6.78847, 21.642, 14.5931, 0.414375, 11.0022, 5.9913, 5.8456, 33.9779, -34.5975, 52.1981, 52.237, -1.9469, 1462.71, 11.4152, 0.474619, 2.9838, 1.40316e+06, 0.03845, 2.14825, 2.06263, 7.05128, 8.242, 5.99025, 278.189, 351.699

2019-12-19 12:30:00, -52.8904, -53.8567, -13.3995, 32.595, 22.0562, 0.4137, 10.9886, 6.10658, 5.97845, 33.9776, -47.038, 51.9449, 51.9911, -2.54061, 1450.5, 10.9181, 0.45262, 2.93065, 1.30754e+06, 0.037225, 1.93197, 2.19833, 7.09923, 8.20022, 5.7148, 264.778, 348.94

2019-12-19 13:30:00, -52.8244, -54.13, -12.1455, 32.58, 22.079, 0.418925, 11.0867, 6.15535, 6.04547, 33.9757, -41.9181, 51.8216, 51.8682, -2.29896, 1444.55, 10.6497, 0.435234, 2.8398, 1.23482e+06, 0.036175, 1.74527, 2.23717, 7.08948, 8.13645, 5.41482, 251.176, 342.86

2019-12-19 14:30:00, -52.7617, -54.3945, -14.8087, 36.8432, 25.0331, 0.46655, 11.985, 6.2406, 6.07292, 33.9714, -42.8205, 51.7768, 51.8189, -2.47754, 1442.33, 10.549, 0.437559, 2.74083, 1.19177e+06, 0.035475, 1.65163, 2.1854, 7.0337, 8.08775, 5.23395, 239.622, 340.389

2019-12-19 15:30:00, -52.7011, -54.6528, -17.1884, 36.0525, 24.656, 0.460075, 11.8618, 6.45453, 6.31003, 33.9494, -53.5906, 51.3369, 51.3919, -2.91646, 1420.98, 10.2305, 0.427062, 2.63065, 1.10739e+06, 0.034575, 1.54873, 2.1246, 6.97868, 8.03092, 5.03383, 232.308, 338.294

2019-12-19 17:30:00, -52.6469, -55.0283, -13.0617, 24.9953, 17.2735, 0.3965, 10.637, 6.79855, 6.64147, 33.915, -62.3591, 50.7065, 50.8046, -3.16205, 1390.54, 9.11932, 0.364366, 2.42148, 868373, 0.031875, 1.31393, 2.03255, 6.9768, 7.93022, 4.72133, 225.455, 337.509

2019-12-19 18:30:00, -52.6599, -55.0758, -13.4864, 25.6241, 17.7191, 0.331225, 9.31215, 6.81913, 6.65081, 33.9122, -62.2367, 50.6711, 50.7887, -3.18281, 1388.83, 8.52415, 0.335565, 2.36183, 778851, 0.030625, 1.1908, 2.0379, 7.02628, 7.87153, 4.5711, 222.293, 337.207

2019-12-19 19:30:00, -52.677, -55.1229, -13.7795, 25.695, 17.782, 0.294275, 8.53133, 6.84492, 6.67564, 33.9103, -63.3261, 50.6227, 50.7449, -3.24027, 1386.52, 8.02503, 0.308958, 2.316, 704735, 0.029525, 1.07742, 2.04883, 7.09057, 7.8285, 4.4387, 218.503, 337.856

2019-12-19 20:30:00, -52.6924, -55.1665, -13.9984, 25.5121, 17.6542, 0.2718, 8.03128, 6.84272, 6.66711, 33.9059, -65.0602, 50.6238, 50.7612, -3.31532, 1386.52, 7.6636, 0.287618, 2.28593, 649181, 0.02855, 0.965225, 2.07062, 7.16415, 7.79412, 4.27343, 214.881, 338.742

2019-12-19 21:30:00, -52.6959, -55.1863, -7.85923, 14.2167, 9.83584, 0.247025, 7.46392, 6.83587, 6.6566, 33.9032, -65.9383, 50.6368, 50.7807, -3.34092, 1387.1, 7.49345, 0.275037, 2.27405, 622174, 0.0278, 0.89355, 2.09062, 7.23878, 7.80087, 4.16805, 212.316, 340.735

2019-12-20 00:30:00, -52.6132, -54.9556, -12.3561, 22.9536, 15.8605, 0.2315, 7.09283, 6.79437, 6.59961, 33.8991, -64.2794, 50.7244, 50.8834, -3.25818, 1391.2, 6.81197, 0.252437, 2.33372, 577917, 0.02715, 0.749175, 2.20852, 7.41818, 7.831, 3.82915, 209.08, 336.422

2019-12-20 01:30:00, -52.6343, -55.008, -8.86295, 16.1595, 11.1695, 0.218975, 6.78258, 6.8049, 6.6083, 33.8973, -65.2664, 50.6958, 50.8684, -3.31792, 1389.82, 6.1544, 0.225904, 2.32575, 513321, 0.0267, 0.5847, 2.24863, 7.47048, 7.7533, 3.289, 212.624, 333.132

2019-12-20 02:30:00, -52.6557, -55.0594, -6.4667, 11.6402, 8.04577, 0.1867, 5.94942, 6.80492, 6.59911, 33.8964, -66.2946, 50.6951, 50.885, -3.36071, 1389.77, 5.78593, 0.204866, 2.3208, 473810, 0.026425, 0.492825, 2.26745, 7.50105, 7.714, 2.9874, 220.549, 330.268

2019-12-20 03:30:00, -52.6745, -55.1028, -7.27232, 12.9659, 8.96246, 0.2079, 6.49142, 6.80645, 6.61213, 33.88, -67.342, 50.7024, 50.866, -3.3932, 1389.92, 6.22488, 0.21363, 2.32457, 507882, 0.0265, 0.56085, 2.2536, 7.49455, 7.75385, 3.302, 228.746, 329.983

2019-12-20 04:30:00, -52.6906, -55.1443, -9.73522, 17.5634, 12.1434, 0.23265, 7.12643, 6.81452, 6.64307, 33.8837, -67.1839, 50.6835, 50.8098, -3.35223, 1389.07, 7.33959, 0.253426, 2.34035, 610135, 0.027075, 0.751125, 2.21177, 7.43843, 7.8429, 3.92248, 234.306, 332.623

2019-12-20 05:30:00, -52.7025, -55.1915, -14.5523, 26.2683, 18.1634, 0.29265, 8.46257, 6.81712, 6.65561, 33.892, -67.1211, 50.6774, 50.7853, -3.35017, 1388.89, 8.32781, 0.297721, 2.36655, 712889, 0.028125, 0.958075, 2.16065, 7.34015, 7.91113, 4.4312, 236.497, 334.315

2019-12-20 06:30:00, -52.7053, -55.22, -12.7804, 23.0021, 15.8963, 0.342225, 9.552, 6.79898, 6.65263, 33.891, -67.6369, 50.6989, 50.7909, -3.36112, 1389.89, 8.58402, 0.316507, 2.38978, 754183, 0.029125, 1.07308, 2.13443, 7.24812, 7.90808, 4.65027, 237.519, 327.438

2019-12-20 08:30:00, -52.5852, -55.5983, -19.5287, 28.9654, 20.0366, 0.317875, 9.0269, 6.8306, 6.66799, 33.8943, -81.092, 50.6764, 50.7626, -4.0774, 1388.87, 8.00337, 0.295343, 2.34335, 682587, 0.029775, 1.01805, 2.1093, 7.10165, 7.72635, 4.44325, 246.609, 269.174

2019-12-20 09:30:00, -52.499, -55.8926, -10.0856, 14.861, 10.281, 0.1955, 6.1852, 6.834, 6.69844, 33.8969, -86.6917, 50.5921, 50.7078, -4.0976, 1384.9, 7.52348, 0.276414, 2.2503, 612085, 0.029, 0.8707, 2.07382, 7.0524, 7.58338, 4.06557, 252.91, 244.117

2019-12-20 10:30:00, -52.4097, -56.1947, -8.84725, 14.7525, 10.159, 0.18845, 6.0344, 6.68197, 6.51232, 33.8909, -72.8483, 50.9118, 51.042, -3.64111, 1399.99, 6.9156, 0.253436, 2.13025, 531571, 0.02775, 0.7084, 2.00802, 7.01, 7.43805, 3.60265, 262.656, 228.616

2019-12-20 11:30:00, -52.3101, -56.4809, -3.55275, 6.07353, 4.19902, 0.17705, 5.7273, 6.81257, 6.58079, 33.8849, -73.003, 50.7796, 50.9208, -3.54445, 1393.65, 6.45569, 0.234894, 2.00125, 463057, 0.026275, 0.609975, 1.9059, 6.9817, 7.35117, 3.4145, 276.218, 212.462

2019-12-20 12:30:00, -52.2263, -56.7684, 8.0644, -14.5924, -10.195, 0.166625, 5.42795, 7.15948, 6.921, 33.8747, -68.3879, 50.1859, 50.3198, -3.31473, 1365.45, 6.03946, 0.218345, 1.8717, 401668, 0.024675, 0.5353, 1.7932, 6.9709, 7.30415, 3.2393, 289.172, 194.83

2019-12-20 13:30:00, -52.1536, -57.059, 5.564, -10.3739, -7.32511, 0.170675, 5.55023, 7.51268, 7.27563, 33.8327, -62.9782, 49.6131, 49.7124, -3.18478, 1338.08, 5.54934, 0.197926, 1.73843, 335722, 0.0227, 0.424825, 1.68513, 7.00793, 7.2702, 2.8636, 304.534, 175.128

2019-12-20 14:30:00, -52.0815, -57.3507, -2.67105, 4.29107, 3.07116, 0.14165, 4.65022, 7.964, 7.74726, 33.7176, -77.5686, 48.8499, 48.9358, -3.64465, 1301.27, 5.22145, 0.176455, 1.59535, 281109, 0.020425, 0.342525, 1.55795, 7.07705, 7.29433, 2.58093, 314.351, 153.162

2019-12-20 15:30:00, -52.0142, -57.6354, -2.0587, 3.7082, 2.6664, 0.091325, 3.02645, 8.12053, 7.96577, 33.6583, -69.4194, 48.4687, 48.5837, -3.22658, 1283.03, 6.08101, 0.201913, 1.44, 301127, 0.018625, 0.371975, 1.3893, 7.03637, 7.36995, 2.5797, 315.893, 139.447

2019-12-20 16:30:00, -51.9746, -57.9078, -6.54117, 13.3662, 9.65072, 0.0725, 2.37498, 8.25908, 7.99593, 33.6193, -60.628, 48.3957, 48.5429, -2.84123, 1279.24, 7.36814, 0.274084, 1.2591, 353238, 0.017975, 0.465475, 1.16595, 6.69292, 7.38738, 2.64043, 319.735, 137.437

2019-12-20 17:30:00, -51.951, -58.1811, -5.19305, 5.22029, 3.85306, 0.153825, 4.50985, 9.00387, 8.68845, 33.5928, -111.873, 47.2808, 47.4148, -5.6602, 1228.17, 8.19898, 0.327834, 0.994775, 333012, 0.0184, 0.529925, 0.8301, 5.8795, 7.33632, 2.68995, 323.258, 136.065

2020-01-13 03:30:00, -67.5986, -68.1015, -5.9616, 6.26932, 3.63767, 0.20025, 6.32113, 1.19672, 1.06487, 32.5761, -115.968, 62.6988, 62.7955, -6.85271, 1971.91, NaN, NaN, NaN, NaN, NaN, NaN, NaN, NaN, NaN, NaN, NaN, NaN

2020-01-13 04:30:00, -67.5986, -68.1015, -10.0212, 10.2368, 5.9417, 0.2238, 6.8962, 1.207, 1.05506, 32.5484, -117.129, 62.7227, 62.8282, -7.05677, 1972.69, NaN, NaN, NaN, NaN, NaN, NaN, NaN, NaN, NaN, NaN, NaN, NaN

2020-01-13 05:30:00, -67.5986, -68.1015, -7.85032, 7.79686, 4.52716, 0.172125, 5.55327, 1.21857, 1.06893, 32.5325, -121.873, 62.6711, 62.7999, -7.25203, 1969.72, NaN, NaN, NaN, NaN, NaN, NaN, NaN, NaN, NaN, NaN, NaN, NaN

2020-01-13 06:30:00, -67.5987, -68.1014, -8.79247, 8.68468, 5.04327, 0.14265, 4.70357, 1.22235, 1.0719, 32.5184, -122.415, 62.6816, 62.7973, -7.29289, 1970.02, NaN, NaN, NaN, NaN, NaN, NaN, NaN, NaN, NaN, NaN, NaN, NaN

2020-01-13 09:30:00, -67.5669, -68.2255, -2.64283, 2.69603, 1.56513, 0.0664, 2.07452, 1.2128, 1.05834, 32.4694, -116.534, 62.7068, 62.846, -7.06195, 1970.51, NaN, NaN, NaN, NaN, NaN, NaN, NaN, NaN, NaN, NaN, NaN, NaN

2020-01-13 10:30:00, -67.5669, -68.2256, -0.4312, 0.428137, 0.248421, 0.053375, 1.61508, 1.19695, 1.05719, 32.4442, -120.521, 62.7512, 62.8571, -7.25909, 1972.41, NaN, NaN, NaN, NaN, NaN, NaN, NaN, NaN, NaN, NaN, NaN, NaN

2020-01-13 11:30:00, -67.567, -68.2256, -0.872375, 0.854754, 0.495899, 0.05075, 1.52842, 1.19305, 1.05326, 32.4277, -122.887, 62.7838, 62.8719, -7.35912, 1973.83, NaN, NaN, NaN, NaN, NaN, NaN, NaN, NaN, NaN, NaN, NaN, NaN

2020-01-13 12:30:00, -67.567, -68.2258, -0.1004, 0.098714, 0.0572895, 0.0683, 2.15862, 1.20348, 1.06453, 32.4464, -122.174, 62.7352, 62.8386, -7.32916, 1971.61, NaN, NaN, NaN, NaN, NaN, NaN, NaN, NaN, NaN, NaN, NaN, NaN

2020-01-15 12:30:00, -67.5326, -68.2789, 0.6717, -0.641012, -0.371357, 0.026825, 0.751825, 1.14795, 1.0711, 32.4297, NaN, 62.8741, 62.8282, -7.56433, 1978.59, NaN, NaN, NaN, NaN, NaN, NaN, NaN, NaN, NaN, NaN, NaN, NaN

2020-11-20 13:30:00, -6.16995, -25.1294, 5.78852, 23.7064, 28.0582, 0.332225, 9.32005, 27.156, 26.8904, 34.1206, 47.4076, 27.6459, 27.8119, 0.860051, 471.659, NaN, NaN, NaN, NaN, NaN, NaN, NaN, NaN, NaN, NaN, NaN, NaN

2020-11-25 06:30:00, -26.8865, -29.3192, -5.92317, 23.013, 24.4982, 0.307267, 8.78243, 22.7861, 22.5876, 36.4356, -20.9587, 30.5746, 30.7384, -1.00734, 582.326, NaN, NaN, NaN, NaN, NaN, NaN, NaN, NaN, NaN, NaN, NaN, NaN

2020-11-25 08:30:00, -27.2342, -29.3963, -6.22192, 23.5441, 24.375, 0.3118, 8.89735, 21.6508, 21.4721, 36.0806, -25.2185, 31.5696, 31.7264, -1.06346, 615.654, NaN, NaN, NaN, NaN, NaN, NaN, NaN, NaN, NaN, NaN, NaN, NaN

2020-11-25 09:30:00, -27.407, -29.4351, -5.65433, 21.5455, 22.3406, 0.31655, 8.99853, 21.714, 21.5089, 36.1541, -25.861, 31.5192, 31.6841, -1.05495, 614.244, NaN, NaN, NaN, NaN, NaN, NaN, NaN, NaN, NaN, NaN, NaN, NaN

2020-11-27 17:30:00, -37.5062, -31.7528, -0.211775, 0.571284, 0.523232, 0.251775, 7.58812, 16.8, 16.7222, 35.5648, -39.6542, 36.2653, 36.3909, -1.68444, 785.182, 8.98192, 0.361694, 3.17362, 1.10735e+06, 0.033675, 1.37902, 2.82673, 7.77003, 8.42865, 4.94505, 261.474, 298.041

2020-11-27 18:30:00, -37.6709, -31.7908, -2.83665, 6.4517, 5.88571, 0.29755, 8.61397, 16.6482, 16.5426, 35.6027, -45.2248, 36.467, 36.5784, -2.00831, 793.15, 8.03509, 0.306257, 3.14893, 952718, 0.0312, 1.08432, 2.95503, 8.03843, 8.5558, 4.41277, 258.729, 292.938

2020-11-27 19:30:00, -37.836, -31.8322, -3.43785, 7.96027, 7.27808, 0.32895, 9.28745, 16.7334, 16.6174, 35.5995, -45.0122, 36.3884, 36.4979, -1.96901, 790.12, 7.95542, 0.294107, 3.12328, 914870, 0.0298, 1.03392, 2.946, 8.18885, 8.68923, 4.3398, 265.469, 289.703

2020-11-27 20:30:00, -37.9996, -31.8736, -6.1638, 14.7843, 13.5043, 0.33645, 9.44475, 16.6964, 16.6081, 35.5935, -44.4714, 36.4001, 36.509, -1.90109, 790.529, 8.02729, 0.294564, 3.10907, 916436, 0.029325, 1.02175, 2.9352, 8.23682, 8.73745, 4.31465, 267.545, 287.369

2020-11-27 21:30:00, -38.1626, -31.9145, -7.70975, 19.7823, 18.0576, 0.376575, 10.2568, 16.671, 16.5727, 35.5626, -39.3872, 36.4451, 36.5528, -1.77891, 792.046, 8.42259, 0.306225, 3.10993, 960608, 0.0295, 1.09608, 2.90815, 8.21482, 8.76925, 4.48635, 264.793, 286.049

2020-11-27 22:30:00, -38.3275, -31.9503, -9.2338, 25.1803, 22.9971, 0.385575, 10.4232, 16.6912, 16.5673, 35.5729, -36.5026, 36.4268, 36.5568, -1.67319, 791.413, 8.8751, 0.326215, 3.12465, 1.02214e+06, 0.030075, 1.25328, 2.85965, 8.15837, 8.82238, 4.83137, 257.958, 285.822

2020-11-27 23:30:00, -38.4919, -31.9904, -9.27305, 24.3444, 22.1984, 0.3556, 9.82467, 16.6303, 16.5502, 35.5543, -37.243, 36.4312, 36.5786, -1.73761, 791.456, 8.85382, 0.330462, 3.1405, 1.02907e+06, 0.030525, 1.3238, 2.84617, 8.1214, 8.82807, 4.9802, 253.739, 284.563

2020-11-28 00:30:00, -38.6533, -32.0314, -12.3105, 28.4722, 25.756, 0.413625, 10.9691, 16.3255, 16.2147, 35.57, -42.7422, 36.7958, 36.9428, -1.99014, 805.572, 8.66125, 0.320623, 3.16502, 1.00861e+06, 0.030625, 1.3155, 2.87723, 8.13748, 8.8205, 5.00432, 251.013, 281.869

2020-11-28 01:30:00, -38.805, -32.0677, -11.0266, 23.6167, 21.3208, 0.424225, 11.1735, 16.2488, 16.0828, 35.6094, -49.3908, 36.931, 37.0816, -2.15684, 811.058, 8.77802, 0.319584, 3.24433, 1.04018e+06, 0.03105, 1.41035, 2.91797, 8.1801, 8.88978, 5.23793, 244.887, 279.216

2020-11-28 02:30:00, -38.9468, -32.1019, -13.58, 29.6698, 26.7528, 0.43025, 11.2834, 16.2022, 16.0987, 35.6321, -48.0214, 36.8978, 37.0599, -2.11226, 809.933, 9.57353, 0.345893, 3.41307, 1.20834e+06, 0.032325, 1.77252, 2.90548, 8.22222, 9.0746, 5.95158, 237.333, 278.814

2020-11-28 07:30:00, -39.5924, -32.2719, -12.2887, 26.1486, 23.1879, 0.31515, 8.96343, 15.5704, 15.4553, 35.344, -46.3544, 37.6231, 37.8363, -2.20328, 836.107, 8.50199, 0.318543, 3.89403, 1.21465e+06, 0.029775, 1.42705, 3.61755, 9.15447, 9.7971, 5.2338, 230.76, 257.537

2020-11-28 08:30:00, -39.7334, -32.3044, -5.77255, 11.9222, 10.4784, 0.281775, 8.23722, 15.2351, 15.0922, 35.2141, -46.813, 38.0608, 38.2793, -2.29217, 852.336, 7.79387, 0.286415, 3.91145, 1.09786e+06, 0.028525, 1.18263, 3.72317, 9.37353, 9.867, 4.78932, 230.315, 255.55

2020-11-28 09:30:00, -39.8776, -32.3424, -2.63303, 5.76391, 4.9991, 0.237, 7.20825, 14.7391, 14.7308, 35.093, -39.3058, 38.5073, 38.7267, -2.18243, 869.074, 7.0036, 0.256392, 3.92115, 982368, 0.02755, 0.907125, 3.80997, 9.5474, 9.88377, 4.15415, 234.135, 253.801

2020-11-28 10:30:00, -40.025, -32.3831, -1.37858, 2.94791, 2.52193, 0.196575, 6.22018, 14.2307, 14.0513, 34.9477, -48.3914, 39.3542, 39.5738, -2.27761, 901.766, 6.47319, 0.232923, 3.92007, 902099, 0.0268, 0.7322, 3.8494, 9.6754, 9.91787, 3.73985, 238.605, 252.83

2020-11-28 11:30:00, -40.1735, -32.427, -0.216025, 0.407963, 0.347346, 0.1847, 5.91013, 14.0543, 13.9274, 34.8934, -53.2062, 39.5217, 39.7368, -2.58745, 908.087, 6.09433, 0.218066, 3.90758, 836359, 0.02625, 0.611, 3.8578, 9.76572, 9.95175, 3.41003, 242.416, 252.367

2020-11-28 12:30:00, -40.3229, -32.4644, 1.15962, -2.1156, -1.80004, 0.188525, 6.0124, 14.0293, 13.8249, 34.7913, -59.0841, 39.6672, 39.8834, -2.68676, 913.159, 5.51876, 0.196575, 3.88545, 745769, 0.02575, 0.479675, 3.85432, 9.82963, 9.96077, 2.9867, 249.162, 251.989

2020-11-28 13:30:00, -40.4721, -32.5044, 2.1959, -3.94138, -3.36535, 0.20035, 6.31765, 14.1596, 13.9928, 34.7762, -57.9179, 39.4678, 39.6788, -2.7183, 905.01, 5.02228, 0.173731, 3.85477, 663676, 0.0253, 0.3962, 3.8336, 9.87615, 9.97563, 2.72155, 259.951, 251.664

2020-11-28 14:30:00, -40.6257, -32.542, 2.25802, -4.19859, -3.58845, 0.208525, 6.52738, 14.1953, 14.0078, 34.7975, -54.8254, 39.4526, 39.6563, -2.62362, 904.564, 5.20205, 0.174581, 3.81933, 671955, 0.024925, 0.4046, 3.7971, 9.9071, 10.0098, 2.8212, 273.312, 251.279

2020-11-28 15:30:00, -40.7803, -32.5801, 2.66508, -4.99594, -4.28041, 0.209425, 6.54908, 14.2859, 14.1254, 34.7878, -50.2607, 39.3144, 39.5138, -2.594, 898.945, 5.72471, 0.190013, 3.77923, 730390, 0.02455, 0.48755, 3.74665, 9.92083, 10.0577, 3.16167, 283.737, 250.715

2020-11-28 16:30:00, -40.9146, -32.6483, -1.91993, 3.49275, 2.98716, 0.19965, 6.30395, 14.2198, 14.0675, 34.7707, -55.6423, 39.3938, 39.5881, -2.67747, 901.999, 6.21289, 0.210459, 3.72757, 790888, 0.02425, 0.561825, 3.68415, 9.91473, 10.0878, 3.39375, 288.457, 250.018

2020-11-28 17:30:00, -41.0594, -32.7016, -1.14787, 2.1444, 1.83381, 0.201725, 6.36175, 14.2163, 14.0637, 34.7663, -55.9109, 39.4112, 39.5936, -2.6084, 902.661, 6.42693, 0.222738, 3.67302, 816631, 0.02405, 0.596175, 3.62345, 9.88895, 10.0841, 3.44053, 288.17, 249.439

2020-11-28 18:30:00, -41.2201, -32.7343, 1.00425, -2.13493, -1.82997, 0.204125, 6.42568, 14.3023, 14.1607, 34.7976, -42.9691, 39.2971, 39.4688, -2.28661, 898.325, 6.17692, 0.217977, 3.61252, 776723, 0.023875, 0.5632, 3.567, 9.8458, 10.03, 3.26777, 288.256, 249.117

2020-11-28 19:30:00, -41.3821, -32.7696, 0.778075, -1.49058, -1.27319, 0.22665, 6.9898, 14.1727, 14.0917, 34.7828, -50.675, 39.4095, 39.556, -2.54324, 902.722, 5.81012, 0.204842, 3.5352, 714339, 0.02365, 0.48395, 3.50073, 9.78645, 9.93597, 2.97665, 293.575, 249.025

2020-11-28 20:30:00, -41.5458, -32.8073, 1.90675, -3.81735, -3.2449, 0.235325, 7.20262, 13.9948, 13.9243, 34.8652, -49.6049, 39.6103, 39.746, -2.44534, 911.438, 5.75949, 0.197411, 3.43998, 681025, 0.0234, 0.4651, 3.40773, 9.7041, 9.8478, 2.9458, 302.784, 248.97

2020-11-28 21:30:00, -41.7098, -32.8453, -0.157375, 0.322625, 0.273748, 0.21245, 6.6381, 13.9282, 13.7842, 34.9904, -48.6886, 39.7501, 39.8952, -2.39739, 918.063, 6.01874, 0.205414, 3.33022, 688075, 0.023175, 0.516675, 3.28885, 9.591, 9.76755, 3.16118, 312.612, 249.007

2020-11-28 22:30:00, -41.874, -32.8804, -4.84405, 10.4204, 8.86064, 0.228975, 7.04093, 14.0066, 13.9499, 35.0364, -43.1639, 39.5442, 39.6813, -2.27396, 910.103, 6.70922, 0.225663, 3.2129, 742841, 0.0231, 0.645075, 3.14562, 9.4422, 9.70638, 3.61585, 317.982, 249.002

2020-11-28 23:30:00, -42.0409, -32.9171, -9.55015, 20.781, 17.6567, 0.2888, 8.40795, 13.9781, 13.8512, 34.9799, -50.9361, 39.6975, 39.8142, -2.25596, 915.853, 7.73392, 0.265083, 3.0964, 837629, 0.023175, 0.8245, 2.98215, 9.25088, 9.66038, 4.15053, 319.073, 248.841

2020-11-29 00:30:00, -42.2103, -32.9546, -8.70735, 17.7569, 15.0793, 0.3545, 9.8046, 13.9588, 13.9122, 34.8764, -45.8107, 39.6593, 39.7589, -2.40342, 913.504, 8.70676, 0.305141, 2.99193, 930725, 0.023625, 1.01757, 2.8105, 9.0101, 9.6109, 4.5875, 318.937, 248.676

2020-11-29 01:30:00, -42.3857, -32.9874, -12.8896, 24.4176, 20.474, 0.389675, 10.5191, 13.4937, 13.5697, 34.7101, -53.7759, 40.1374, 40.218, -2.61162, 931.561, 9.61298, 0.348411, 2.91355, 1.03104e+06, 0.0246, 1.24057, 2.63195, 8.7135, 9.56535, 4.97527, 316.494, 248.351

2020-11-29 02:30:00, -42.5587, -33.0271, -10.7826, 20.3762, 16.643, 0.36295, 9.98522, 12.5441, 12.5992, 34.5489, -52.7914, 41.4347, 41.4998, -2.69047, 983.558, 10.0734, 0.38419, 2.86108, 1.10294e+06, 0.02605, 1.43855, 2.47058, 8.39468, 9.49202, 5.23093, 312.415, 248.695

2020-11-29 03:30:00, -42.7262, -33.0722, -8.1346, 14.5397, 11.7093, 0.3123, 8.93342, 12.0386, 12.0497, 34.4603, -57.1782, 42.1919, 42.2527, -2.88942, 1014.45, 10.1108, 0.393899, 2.81978, 1.10743e+06, 0.027375, 1.54895, 2.35445, 8.12402, 9.36623, 5.31685, 310.085, 250.616

2020-11-29 04:30:00, -42.8912, -33.1147, -7.5434, 13.2251, 10.5437, 0.310175, 8.89225, 11.6798, 11.6935, 34.4394, -59.2289, 42.6948, 42.744, -2.97625, 1035.5, 10.0969, 0.394494, 2.79127, 1.09684e+06, 0.028575, 1.63587, 2.2599, 7.91015, 9.23728, 5.43645, 308.284, 252.874

2020-11-29 05:30:00, -43.0557, -33.1578, -8.21795, 14.5731, 11.5541, 0.295775, 8.57897, 11.4838, 11.4781, 34.4286, -57.4228, 42.9987, 43.045, -2.96091, 1048.32, 10.0384, 0.388868, 2.774, 1.07039e+06, 0.029525, 1.68962, 2.19835, 7.75802, 9.07018, 5.5947, 308.771, 254.695

2020-11-29 06:30:00, -43.2192, -33.2023, -8.71895, 16.016, 12.6847, 0.304875, 8.77325, 11.4464, 11.3677, 34.44, -56.8559, 43.1521, 43.197, -2.86835, 1054.97, 9.94214, 0.381748, 2.76887, 1.05502e+06, 0.030125, 1.71893, 2.16905, 7.6721, 8.92748, 5.73442, 310.235, 256.229

2020-11-29 07:30:00, -43.3833, -33.246, -11.5347, 21.3569, 17.0105, 0.35275, 9.777, 11.6461, 11.599, 34.4894, -57.6583, 42.825, 42.8642, -2.82712, 1041.47, 10.1205, 0.389268, 2.7866, 1.08834e+06, 0.0306, 1.7744, 2.1468, 7.63795, 8.90635, 5.8404, 309.654, 257.156

2020-11-29 08:30:00, -43.5485, -33.2885, -9.11273, 17.5731, 13.9813, 0.35905, 9.90735, 11.6072, 11.556, 34.5508, -56.2437, 42.8729, 42.9107, -2.71784, 1044.06, 10.2968, 0.401928, 2.82945, 1.13127e+06, 0.030925, 1.83988, 2.1476, 7.6515, 8.94003, 5.95688, 308.762, 257.237

2020-11-29 09:30:00, -43.7146, -33.3346, -8.92105, 19.7758, 15.7001, 0.380875, 10.3467, 11.5313, 11.5159, 34.6278, -46.035, 42.9189, 42.9501, -2.36702, 1046.72, 10.0147, 0.38748, 2.8828, 1.10529e+06, 0.03095, 1.7566, 2.28095, 7.72518, 8.91152, 5.78855, 310.466, 261.162

2020-11-29 10:30:00, -43.8792, -33.3792, -7.4419, 15.3679, 12.1795, 0.3571, 9.86675, 11.4698, 11.4935, 34.6142, -49.2156, 42.9535, 42.9841, -2.54214, 1048.08, 10.0338, 0.384066, 2.94317, 1.13048e+06, 0.0308, 1.75028, 2.36415, 7.82627, 8.9733, 5.80537, 310.035, 262.469

2020-11-29 11:30:00, -44.0403, -33.422, -9.02372, 19.708, 15.6727, 0.352825, 9.7807, 11.5908, 11.5462, 34.6417, -51.0986, 42.8704, 42.9051, -2.40054, 1044.78, 9.99102, 0.384365, 3.00705, 1.144e+06, 0.03055, 1.77493, 2.42427, 7.93723, 9.06493, 5.90415, 308.215, 262.204

2020-11-29 12:30:00, -44.1996, -33.4647, -3.45838, 7.37746, 5.85267, 0.327925, 9.2654, 11.505, 11.5815, 34.623, -46.7414, 42.8217, 42.86, -2.4538, 1042.54, 9.72213, 0.368985, 3.06365, 1.12158e+06, 0.030225, 1.71245, 2.53735, 8.05805, 9.11905, 5.80732, 308.472, 263.639

2020-11-29 13:30:00, -44.3617, -33.5082, -3.21745, 7.63448, 6.011, 0.34805, 9.67765, 11.2387, 11.1271, 34.5848, -45.9754, 43.4705, 43.505, -2.23661, 1069.95, 9.55103, 0.359729, 3.11033, 1.11486e+06, 0.0298, 1.652, 2.63203, 8.1751, 9.18327, 5.69955, 310.531, 264.484

2020-11-29 14:30:00, -44.5247, -33.5543, -4.07488, 10.4493, 8.32522, 0.3849, 10.4267, 11.6567, 11.6187, 34.6677, -37.4387, 42.7524, 42.7992, -2.0397, 1039.99, 9.59177, 0.361182, 3.1472, 1.13271e+06, 0.02945, 1.61855, 2.69647, 8.27652, 9.2648, 5.625, 312.118, 265.029

2020-11-29 15:30:00, -44.6937, -33.5955, -2.33013, 5.74825, 4.51821, 0.38535, 10.4349, 11.179, 11.4333, 34.6092, -49.36, 43.0368, 43.0692, -2.1295, 1051.58, 9.54198, 0.359284, 3.17502, 1.13465e+06, 0.029075, 1.57132, 2.75715, 8.36202, 9.32333, 5.50868, 313.338, 266.082

2020-11-29 16:30:00, -44.8665, -33.6425, 0.34615, -0.867401, -0.658568, 0.3632, 9.9926, 9.97193, 10.0769, 34.4337, -47.5031, 45.0579, 45.0666, -2.182, 1137.62, 9.45969, 0.354545, 3.19175, 1.12585e+06, 0.028775, 1.52412, 2.8028, 8.42833, 9.35287, 5.41485, 314.502, 267.209

2020-11-29 17:30:00, -45.0418, -33.6932, 0.391375, -0.923843, -0.690901, 0.374425, 10.2157, 9.4524, 9.53441, 34.3837, -34.2999, 45.8985, 45.8997, -2.35376, 1174.39, 9.49729, 0.352592, 3.1989, 1.12758e+06, 0.0286, 1.52358, 2.81087, 8.46665, 9.39255, 5.4219, 315.315, 267.707

2020-11-29 18:30:00, -45.2172, -33.7437, -8.16187, 16.6857, 12.4961, 0.38745, 10.4755, 9.5008, 9.49441, 34.4123, -50.6265, 45.9433, 45.9546, -2.72139, 1176.68, 9.41202, 0.35367, 3.19667, 1.12173e+06, 0.028475, 1.52163, 2.80852, 8.47852, 9.40053, 5.43205, 317.057, 268.311

2020-11-29 19:30:00, -45.389, -33.8015, -14.3897, 30.1699, 22.6534, 0.410675, 10.929, 9.58995, 9.51006, 34.4381, -49.3953, 45.9051, 45.9246, -2.65267, 1175.24, 9.19689, 0.344277, 3.18112, 1.08711e+06, 0.028325, 1.46212, 2.82267, 8.48088, 9.36137, 5.30457, 321.803, 269.706

2020-11-29 20:30:00, -45.5615, -33.8571, -14.2276, 27.3381, 20.6808, 0.389675, 10.5149, 9.84625, 9.81365, 34.5092, -54.363, 45.4096, 45.4453, -2.86738, 1153.92, 9.08142, 0.337195, 3.15508, 1.06126e+06, 0.028075, 1.42727, 2.8119, 8.4824, 9.33735, 5.27727, 328.136, 270.397

2020-11-29 21:30:00, -45.7381, -33.892, -14.0897, 29.1064, 21.9102, 0.388, 10.4867, 9.6767, 9.74884, 34.4635, -50.7496, 45.52, 45.554, -2.67098, 1158.36, 9.44378, 0.347621, 3.12908, 1.09565e+06, 0.027925, 1.50398, 2.7417, 8.47438, 9.4026, 5.49458, 332.217, 269.285

2020-11-29 22:30:00, -45.9119, -33.9322, -6.94243, 15.3383, 11.4461, 0.385725, 10.4412, 9.3786, 9.33853, 34.3674, -46.7668, 46.1793, 46.2057, -2.52917, 1186.77, 9.77356, 0.363982, 3.11385, 1.12954e+06, 0.0279, 1.57975, 2.682, 8.4615, 9.46415, 5.6635, 335.497, 268.132

2020-11-29 23:30:00, -46.0799, -33.9799, -6.24805, 13.0524, 9.6285, 0.3643, 10.014, 8.98503, 9.12595, 34.2631, -46.1263, 46.5452, 46.5616, -2.69001, 1202.11, NaN, NaN, NaN, NaN, NaN, NaN, NaN, NaN, NaN, NaN, NaN, NaN

2020-11-30 00:30:00, -46.2449, -34.0235, -9.18517, 17.8153, 12.8763, 0.389175, 10.5003, 8.2937, 8.37203, 34.1723, -49.7645, 47.7965, 47.7888, -2.9648, 1257.91, NaN, NaN, NaN, NaN, NaN, NaN, NaN, NaN, NaN, NaN, NaN, NaN

2020-11-30 10:30:00, -47.8791, -34.48, -26.3492, 60.9067, 42.0838, 0.493925, 12.2029, 6.79305, 6.84136, 33.9802, NaN, 50.4929, 50.4332, -2.60748, 1381.22, NaN, NaN, NaN, NaN, NaN, NaN, NaN, NaN, NaN, NaN, NaN, NaN

2020-11-30 11:30:00, -48.0195, -34.521, -17.3365, 32.4103, 22.4091, 0.329525, 9.07765, 6.8152, 6.78495, 33.9813, -57.8563, 50.503, 50.5326, -3.22501, 1381.71, NaN, NaN, NaN, NaN, NaN, NaN, NaN, NaN, NaN, NaN, NaN, NaN

2020-11-30 12:30:00, -48.1384, -34.5562, -29.2222, 59.4455, 41.2005, 0.667875, 15.3859, 6.89445, 6.83698, 33.9856, -51.3824, 50.3428, 50.4395, -2.95555, 1374.19, NaN, NaN, NaN, NaN, NaN, NaN, NaN, NaN, NaN, NaN, NaN, NaN

2020-11-30 13:30:00, -48.2347, -34.5835, -21.6578, 51.3867, 35.7251, 0.7211, 16.2324, 6.99653, 6.8557, 34.0439, -43.179, 50.2643, 50.3916, -2.53208, 1371.18, NaN, NaN, NaN, NaN, NaN, NaN, NaN, NaN, NaN, NaN, NaN, NaN

2020-11-30 14:30:00, -48.3025, -34.6032, -21.4679, 50.5277, 34.6567, 0.676325, 15.5323, 6.5507, 6.7302, 34.0102, -38.7227, 50.4986, 50.6222, -2.55834, 1381.85, NaN, NaN, NaN, NaN, NaN, NaN, NaN, NaN, NaN, NaN, NaN, NaN

2020-11-30 15:30:00, -48.3594, -34.6227, -27.6824, 55.7723, 36.8277, 0.582325, 14.004, 5.30938, 5.3197, 33.8798, -49.7456, 53.1598, 53.2543, -3.12522, 1508.1, NaN, NaN, NaN, NaN, NaN, NaN, NaN, NaN, NaN, NaN, NaN, NaN

2020-11-30 16:30:00, -48.4236, -34.6459, -24.0635, 45.6639, 29.4312, 0.49605, 12.507, 4.52675, 4.62767, 33.8319, -54.2726, 54.5395, 54.6141, -3.38959, 1575.2, NaN, NaN, NaN, NaN, NaN, NaN, NaN, NaN, NaN, NaN, NaN, NaN

2020-11-30 17:30:00, -48.4974, -34.6687, -20.8384, 37.5741, 23.97, 0.424525, 11.1883, 4.19723, 4.14416, 33.7934, -56.4754, 55.5284, 55.5955, -3.62497, 1623.79, NaN, NaN, NaN, NaN, NaN, NaN, NaN, NaN, NaN, NaN, NaN, NaN

2020-11-30 18:30:00, -48.5778, -34.6948, -15.3778, 28.1433, 17.9913, 0.3458, 9.63373, 4.26438, 4.17656, 33.7888, -57.1857, 55.4565, 55.531, -3.56807, 1620.13, NaN, NaN, NaN, NaN, NaN, NaN, NaN, NaN, NaN, NaN, NaN, NaN

2020-11-30 19:30:00, -48.6733, -34.7325, -11.658, 20.6048, 13.1839, 0.29105, 8.47847, 4.2931, 4.33329, 33.8122, -59.1853, 55.1425, 55.2076, -3.67466, 1604.82, NaN, NaN, NaN, NaN, NaN, NaN, NaN, NaN, NaN, NaN, NaN, NaN

2020-11-30 20:30:00, -48.7847, -34.7716, -6.37622, 11.7446, 7.48817, 0.287275, 8.38935, 4.17935, 3.97809, 33.8496, -60.8122, 55.8633, 55.9185, -3.57071, 1641.33, NaN, NaN, NaN, NaN, NaN, NaN, NaN, NaN, NaN, NaN, NaN, NaN

2020-11-30 21:30:00, -48.9071, -34.805, -3.73057, 8.13467, 5.30878, 0.256675, 7.70852, 4.92905, 4.90184, 33.8965, -45.3388, 53.9735, 54.0568, -2.92635, 1548.17, NaN, NaN, NaN, NaN, NaN, NaN, NaN, NaN, NaN, NaN, NaN, NaN

2020-11-30 22:30:00, -48.9973, -34.8251, -7.13103, 13.5114, 8.94108, 0.2853, 8.33207, 5.37897, 5.27643, 33.9191, -56.5528, 53.1593, 53.3263, -3.32498, 1508.59, NaN, NaN, NaN, NaN, NaN, NaN, NaN, NaN, NaN, NaN, NaN, NaN

2020-12-08 18:30:00, -53.2176, -43.0821, -11.9558, 22.274, 13.8471, 0.2558, 7.67802, 3.37222, 3.33443, 33.868, -67.3013, 57.1636, 57.2557, -3.59681, 1707.14, 8.35015, 0.314897, 3.79455, 1.16444e+06, 0.026125, 1.19035, 3.5979, 9.64585, 10.2102, 4.76555, 223.1, 227.681

2020-12-08 19:30:00, -53.1916, -43.3353, -8.20308, 15.2891, 9.48255, 0.22255, 6.89637, 3.29773, 3.27203, 33.8591, -54.9217, 57.298, 57.3909, -3.60204, 1713.82, 7.42182, 0.274577, 3.67915, 995244, 0.02535, 0.903725, 3.56343, 9.64173, 10.029, 4.06188, 226.79, 227.273

2020-12-08 20:30:00, -53.1628, -43.597, -10.2216, 19.5955, 12.1404, 0.226025, 6.98093, 3.26342, 3.20088, 33.8521, -53.718, 57.4435, 57.5447, -3.51012, 1721.11, 7.15272, 0.260378, 3.5708, 922268, 0.024625, 0.78535, 3.48212, 9.63465, 9.95877, 3.78825, 233.358, 226.983

2020-12-08 21:30:00, -53.1347, -43.8662, -8.10695, 15.4047, 9.55718, 0.220125, 6.84235, 3.30737, 3.28829, 33.8456, -53.5225, 57.2528, 57.3603, -3.53026, 1711.32, 7.31378, 0.258535, 3.47005, 903998, 0.02405, 0.816225, 3.3709, 9.6116, 9.96915, 3.96165, 236.088, 226.822

2020-12-08 22:30:00, -53.1078, -44.1422, -7.06317, 13.2616, 8.19534, 0.22435, 6.94525, 3.18257, 3.14802, 33.8491, -54.158, 57.5552, 57.6587, -3.5892, 1726.74, 7.15811, 0.259019, 3.3712, 860042, 0.0235, 0.76915, 3.28037, 9.58312, 9.92415, 3.82425, 236.268, 226.793

2020-12-08 23:30:00, -53.0817, -44.4228, -12.0763, 22.3448, 13.8105, 0.2507, 7.5559, 3.18708, 3.11762, 33.8505, -57.3913, 57.6234, 57.7235, -3.64655, 1730.23, 6.68735, 0.241423, 3.27018, 776508, 0.0229, 0.66125, 3.2008, 9.56263, 9.84532, 3.49653, 238.645, 226.652

2020-12-09 00:30:00, -53.053, -44.7018, -11.2944, 21.8175, 13.5189, 0.1975, 6.1256, 3.26778, 3.21721, 33.8396, -52.6086, 57.4044, 57.5135, -3.48098, 1718.93, 6.23803, 0.223797, 3.1693, 700476, 0.022275, 0.570275, 3.11578, 9.54753, 9.78208, 3.2276, 242.364, 226.465

2020-12-09 01:30:00, -53.0216, -44.981, -6.47783, 11.1873, 6.94368, 0.13805, 4.56977, 3.32125, 3.253, 33.8424, -58.9715, 57.2917, 57.4364, -3.88716, 1713.25, 5.74219, 0.203146, 3.0708, 619505, 0.0216, 0.471, 3.0331, 9.53422, 9.71542, 2.90282, 246.323, 226.405

2020-12-09 02:30:00, -52.9884, -45.2622, -4.91398, 9.02115, 5.61892, 0.13775, 4.57505, 3.43303, 3.30678, 33.877, -68.9592, 57.166, 57.3117, -3.65175, 1707.39, 5.37854, 0.192492, 2.97625, 557625, 0.021025, 0.406575, 2.94753, 9.51887, 9.66765, 2.70175, 248.776, 226.396

2020-12-09 03:30:00, -52.9605, -45.5453, -4.10545, 11.2015, 7.07788, 0.144325, 4.7767, 3.8912, 3.76847, 33.9129, -37.7323, 56.1651, 56.3324, -2.42059, 1657.39, 5.06283, 0.173692, 2.88548, 499247, 0.0205, 0.364125, 2.8617, 9.50183, 9.63198, 2.57715, 251.692, 226.46

2020-12-09 04:30:00, -52.9307, -45.8312, -5.38485, 12.5131, 7.98504, 0.1486, 4.90308, 4.20697, 4.11307, 33.9199, -38.4365, 55.4219, 55.6227, -2.80934, 1620.27, 5.05371, 0.171826, 2.798, 478641, 0.0199, 0.3557, 2.77475, 9.48873, 9.6185, 2.6088, 259.594, 226.481

2020-12-09 05:30:00, -52.9012, -46.1203, -4.57618, 8.02558, 5.17955, 0.1398, 4.62223, 4.56977, 4.38925, 33.9457, -59.8712, 54.8475, 55.0574, -3.69151, 1592.03, 5.04446, 0.171183, 2.7134, 459095, 0.0193, 0.343275, 2.69123, 9.48133, 9.608, 2.61955, 271.506, 226.598

2020-12-09 06:30:00, -52.8706, -46.4124, -1.09912, 1.90044, 1.23984, 0.115425, 3.80848, 4.9184, 4.79337, 33.9755, -63.7503, 54.0248, 54.2473, -3.69531, 1551.77, 4.61185, 0.157027, 2.63203, 398744, 0.018775, 0.285575, 2.61578, 9.48057, 9.57925, 2.40955, 285.293, 227.033

2020-12-09 07:30:00, -52.8385, -46.7077, -5.1306, 9.77282, 6.36819, 0.109925, 3.62695, 4.8801, 4.73804, 33.9849, -55.7813, 54.1101, 54.3534, -3.35913, 1556.09, 3.87514, 0.130481, 2.55395, 318296, 0.0182, 0.18705, 2.54643, 9.48448, 9.53872, 1.96352, 303.154, 227.744

2020-12-09 08:30:00, -52.8084, -47.004, -4.999, 10.1069, 6.60094, 0.1211, 3.99878, 4.95395, 4.834, 34.0042, -46.4884, 53.8979, 54.1597, -3.15385, 1545.91, 3.30775, 0.105386, 2.4794, 258113, 0.01765, 0.1149, 2.47645, 9.48513, 9.51338, 1.62157, 329.965, 228.436

2020-12-09 09:30:00, -52.7756, -47.3049, -5.76677, 9.69528, 6.30488, 0.14145, 4.66775, 4.81475, 4.70607, 33.9719, -61.8, 54.1868, 54.42, -3.80966, 1559.7, 3.61853, 0.108121, 2.40687, 271025, 0.01715, 0.1004, 2.40457, 9.47822, 9.5018, 1.51117, 10.9835, 229.041

2020-12-09 10:30:00, -52.7411, -47.6178, -2.90813, 4.98148, 3.2414, 0.1622, 5.28127, 4.83383, 4.70345, 33.9716, -61.8202, 54.2256, 54.4253, -3.7422, 1561.61, 4.72645, 0.141299, 2.33487, 349942, 0.01675, 0.156875, 2.32892, 9.45155, 9.4976, 1.69165, 15.4884, 229.596

2020-12-09 11:30:00, -52.71, -47.9352, 1.2795, -2.22491, -1.45284, 0.2249, 6.92448, 4.94765, 4.8533, 33.9749, -60.3127, 53.9692, 54.13, -3.67114, 1549.02, 5.98594, 0.194655, 2.26632, 457442, 0.016525, 0.273775, 2.24852, 9.37615, 9.49587, 2.08765, 29.0946, 230.181

2020-12-09 12:30:00, -52.6773, -48.2529, -5.80085, 9.00573, 5.88488, 0.2995, 8.65865, 4.97103, 4.93095, 33.9449, -61.8633, 53.8558, 53.9867, -4.10313, 1543.04, 6.89025, 0.244999, 2.2059, 556558, 0.016575, 0.4211, 2.16338, 9.22963, 9.49597, 2.5145, 38.2176, 230.752

2020-12-09 13:30:00, -52.6378, -48.5685, -8.83292, 11.336, 7.3877, 0.32655, 9.2358, 4.88418, 4.82939, 33.8536, -89.9389, 54.0865, 54.2104, -4.98023, 1553.15, 7.62609, 0.293755, 2.1591, 647505, 0.017075, 0.592175, 2.074, 8.99493, 9.49545, 2.96867, 41.466, 231.368

2020-12-09 14:30:00, -52.6032, -48.8795, -11.8236, 17.9208, 11.7255, 0.348075, 9.67907, 5.01248, 4.87588, 33.9368, -73.0278, 53.977, 54.0964, -4.21303, 1548.89, 8.35978, 0.336534, 2.13295, 725693, 0.01815, 0.7704, 1.98647, 8.67628, 9.47523, 3.40095, 45.1055, 232.598

2020-12-09 15:30:00, -52.5737, -49.19, -12.0889, 24.468, 16.1685, 0.397425, 10.666, 5.3328, 5.24987, 34.0042, -63.6491, 53.2426, 53.354, -3.11813, 1513.79, 8.65375, 0.357492, 2.12343, 754322, 0.019525, 0.9024, 1.921, 8.34852, 9.3751, 3.7013, 50.0397, 235.743

2020-12-09 16:30:00, -52.5407, -49.5013, -10.3856, 39.7557, 26.4649, 0.39455, 10.6083, 5.57228, 5.44706, 34.0361, -21.8, 52.8625, 52.9695, -1.63919, 1495.67, 8.44524, 0.344542, 2.11752, 717006, 0.0207, 0.961225, 1.88618, 8.09558, 9.19003, 3.89135, 53.4021, 241.064

2020-12-09 18:30:00, -52.4764, -50.1113, -4.97598, 13.427, 9.05941, 0.2947, 8.55157, 6.01158, 5.9246, 34.0317, -31.2178, 51.9769, 52.0763, -2.2913, 1452.73, 6.57149, 0.254942, 2.0773, 507185, 0.02135, 0.743125, 1.93253, 7.89188, 8.54445, 3.60072, 52.7659, 261.996

2020-12-09 19:30:00, -52.4448, -50.4159, -4.02438, 9.7698, 6.61222, 0.2371, 7.24078, 6.11265, 6.09824, 34.0164, -45.8145, 51.6584, 51.7606, -2.53221, 1437.22, 5.18005, 0.193847, 2.03923, 374789, 0.020975, 0.442175, 1.9853, 7.892, 8.16157, 2.74487, 45.4051, 278.649

2020-12-09 22:30:00, -52.3455, -51.3314, -1.2903, 3.10006, 2.1569, 0.119625, 3.98653, 7.02217, 6.90907, 34.0384, -35.7244, 50.1377, 50.2991, -2.49444, 1365.14, 2.44985, 0.0801047, 1.90338, 148006, 0.0196, 0.073475, 1.9017, 7.88575, 7.89695, 1.22585, 60.7289, 304.95

2020-12-09 23:30:00, -52.31, -51.6324, -2.48315, 4.09501, 2.85328, 0.120575, 4.0197, 7.07017, 6.98933, 34.0401, -60.2274, 50.0028, 50.1578, -3.62516, 1358.81, 2.36174, 0.0732023, 1.86268, 139208, 0.0192, 0.06685, 1.86118, 7.88235, 7.89277, 1.21835, 86.3356, 308.625

2020-12-10 00:30:00, -52.2762, -51.9431, -3.57553, 5.98419, 4.14859, 0.132, 4.3948, 6.903, 6.87393, 34.0358, -75.5805, 50.2201, 50.3616, -3.58462, 1369, 3.23274, 0.0948143, 1.83597, 187883, 0.018875, 0.128775, 1.82995, 7.8886, 7.92715, 1.63635, 112.339, 312.231

2020-12-10 01:30:00, -52.242, -52.2652, -2.16112, 4.25479, 2.91177, 0.190425, 6.02248, 6.47663, 6.4975, 34.0322, -42.9173, 50.9221, 51.032, -3.08353, 1402.22, 4.874, 0.145504, 1.83505, 291219, 0.0189, 0.29325, 1.80765, 7.8856, 8.0403, 2.49523, 128.337, 315.708

2020-12-10 02:30:00, -52.2062, -52.6009, -4.89145, 7.73223, 5.25021, 0.27455, 8.10437, 6.21903, 6.22543, 34.0252, -79.5992, 51.4438, 51.5258, -3.87487, 1427.04, 6.73488, 0.223016, 1.87528, 438038, 0.0196, 0.508825, 1.80083, 7.8279, 8.20717, 3.1974, 135.509, 318.684

2020-12-10 03:30:00, -52.171, -52.9433, -3.61558, 13.9517, 9.46292, 0.286725, 8.37582, 6.1833, 6.12509, 34.0449, -22.31, 51.6233, 51.7039, -1.5928, 1435.89, 7.68234, 0.27676, 1.95708, 552006, 0.021, 0.7135, 1.81927, 7.72628, 8.3628, 3.60363, 139.202, 321.543

2020-12-10 04:30:00, -52.1355, -53.2789, -4.50903, 13.4436, 9.0935, 0.345525, 9.6217, 6.09403, 6.06718, 34.0381, -34.8173, 51.7428, 51.8118, -2.06526, 1441.55, 8.13974, 0.308763, 2.0594, 638392, 0.022525, 0.874325, 1.86242, 7.65375, 8.477, 3.91392, 144.521, 324.738

2020-12-10 06:30:00, -52.0651, -53.9259, -3.2704, 9.03963, 6.27885, 0.379325, 10.2958, 6.96652, 6.88652, 34.0376, -37.7175, 50.249, 50.339, -2.17245, 1370.38, 9.30892, 0.348416, 2.28295, 822292, 0.0257, 1.2112, 1.93007, 7.5421, 8.7067, 4.57373, 160.926, 331.883

2020-12-10 08:30:00, -51.9965, -54.5429, -5.7698, 15.4363, 10.7115, 0.43105, 11.2975, 6.93427, 6.8862, 34.0245, -33.5455, 50.2575, 50.3429, -2.24436, 1370.63, 10.4187, 0.428308, 2.45275, 1.04207e+06, 0.029525, 1.55942, 1.89195, 7.29405, 8.83405, 5.03388, 165.404, 334.999

2020-12-10 14:30:00, -51.8009, -56.3742, -4.643, 9.24548, 6.53236, 0.1727, 5.58268, 7.53322, 7.39745, 33.9905, -51.1805, 49.3005, 49.4631, -2.96576, 1325.33, 5.94377, 0.211252, 1.7395, 362040, 0.024025, 0.52465, 1.65777, 6.81188, 7.17885, 3.14538, 201.03, 141.394

2020-12-10 15:30:00, -51.7665, -56.6774, -6.32592, 9.98954, 7.08634, 0.1409, 4.64387, 7.66657, 7.57836, 33.9943, -61.382, 48.9658, 49.1533, -3.71664, 1309.81, 5.6719, 0.197764, 1.60745, 316672, 0.02295, 0.470975, 1.5364, 6.70073, 7.04997, 3.00188, 199.202, 137.75

2020-12-10 16:30:00, -51.7336, -56.9794, -8.05685, 8.32595, 5.92465, 0.160925, 5.21875, 7.77065, 7.59907, 33.867, -99.2923, 48.9707, 49.1497, -5.67916, 1308.57, 5.64845, 0.196191, 1.49202, 290331, 0.022175, 0.456275, 1.42025, 6.5657, 6.93778, 2.98742, 199.319, 131.927

2020-12-10 17:30:00, -51.7006, -57.2818, -10.6777, 10.4448, 7.54759, 0.19135, 6.08052, 8.28655, 8.08816, 33.72, NaN, 48.1737, 48.365, -5.91146, 1270.18, 5.64097, 0.192486, 1.37553, 264388, 0.021525, 0.4533, 1.2981, 6.3965, 6.80863, 3.04975, 196.514, 123.453

2020-12-13 11:30:00, -53.435, -55.6374, -10.6071, 19.1061, 13.3954, 0.340475, 9.48002, 7.2761, 6.48023, 33.9965, -55.2335, 50.8972, 51.0722, -3.38161, 1400.6, 9.66434, 0.366747, 2.73325, 993997, 0.026675, 1.76435, 2.08087, 8.1013, 9.73753, 5.89625, 262.825, 228.172

2020-12-13 12:30:00, -53.5702, -55.4884, -6.2101, 11.6185, 7.95904, 0.32425, 9.1737, 6.5093, 6.44115, 34.028, -59.6819, 50.9807, 51.1344, -3.24907, 1404.96, 9.53519, 0.358462, 2.8229, 1.00612e+06, 0.027175, 1.7611, 2.20035, 8.1574, 9.5708, 6.0224, 265.983, 228.456

2020-12-13 13:30:00, -53.6997, -55.3365, -9.6124, 25.5588, 17.5555, 0.4266, 11.1917, 6.59735, 6.4523, 34.0201, -40.4959, 50.9521, 51.1164, -2.28579, 1403.5, 9.55327, 0.357628, 2.91405, 1.03866e+06, 0.027525, 1.7188, 2.34563, 8.23655, 9.48297, 5.97607, 269.488, 229.328

2020-12-13 14:30:00, -53.8261, -55.1784, -7.94448, 24.3679, 16.8112, 0.383175, 10.3515, 6.74225, 6.62923, 34.0176, -28.454, 50.6295, 50.8, -1.97025, 1388.14, 9.48823, 0.357243, 2.994, 1.05903e+06, 0.02765, 1.64875, 2.4868, 8.3264, 9.44632, 5.81827, 271.738, 231.338

2020-12-13 15:30:00, -53.9542, -55.0191, -0.794875, 2.41088, 1.66343, 0.27155, 8.01955, 6.74595, 6.64666, 34.0258, -31.1419, 50.5825, 50.7668, -1.99074, 1386.02, 8.83266, 0.336431, 3.04443, 994981, 0.02745, 1.42303, 2.6707, 8.42563, 9.32578, 5.2739, 274.205, 235.773

2020-12-13 16:30:00, -54.0846, -54.8675, -1.25473, 3.89076, 2.68315, 0.227425, 7.00197, 6.72918, 6.6231, 34.0219, -28.2522, 50.6271, 50.8098, -1.9487, 1388.08, 7.68042, 0.290313, 3.0511, 861111, 0.026825, 1.08203, 2.8379, 8.53375, 9.14805, 4.43532, 277.223, 240.996

2020-12-13 17:30:00, -54.2164, -54.7117, 1.86005, -6.01245, -4.13865, 0.163875, 5.29017, 6.6683, 6.65129, 34.0113, -26.8051, 50.5631, 50.7623, -1.86629, 1384.92, 6.63736, 0.249215, 3.01895, 726096, 0.0259, 0.76835, 2.91207, 8.6419, 9.01685, 3.64085, 282.075, 244.856

2020-12-13 18:30:00, -54.3509, -54.5485, -0.2822, 0.849139, 0.576221, 0.087325, 2.88585, 6.19925, 6.0278, 34.0085, -30.8042, 51.6719, 51.892, -2.04439, 1437.77, 5.50394, 0.202388, 2.96067, 578403, 0.0248, 0.517825, 2.91133, 8.7467, 8.95397, 2.94998, 290.734, 247.743

2020-12-13 19:30:00, -54.4912, -54.3819, -1.25022, 3.88249, 2.63006, 0.08425, 2.77995, 6.1423, 6.07924, 34.0302, -28.3497, 51.574, 51.7918, -1.97667, 1433.34, 4.60069, 0.163936, 2.88845, 456062, 0.023625, 0.3322, 2.86765, 8.84828, 8.95478, 2.37378, 304.058, 250.263

2020-12-13 20:30:00, -54.6308, -54.2106, -2.10233, 7.49075, 4.84229, 0.09865, 3.27428, 4.6224, 4.91255, 33.9206, -31.023, 53.8886, 54.0292, -1.78512, 1544.32, 4.02371, 0.135912, 2.81173, 373089, 0.022525, 0.228125, 2.80195, 8.94567, 9.00703, 2.02153, 320.282, 252.434

2020-12-13 21:30:00, -54.7679, -54.0539, -1.4602, 4.29056, 2.6709, 0.137275, 4.55898, 3.4149, 3.37044, 33.7907, -28.7233, 57.1079, 57.2021, -2.27794, 1703.16, 3.67761, 0.119865, 2.7355, 324040, 0.02145, 0.1746, 2.72942, 9.0386, 9.08188, 1.83372, 328.089, 254.31

2020-12-13 22:30:00, -54.8998, -53.911, -2.60255, 5.5909, 3.48308, 0.175, 5.66818, 3.43972, 3.37796, 33.7865, -47.452, 57.0834, 57.1874, -3.11478, 1701.86, 3.96791, 0.1235, 2.66393, 337888, 0.020475, 0.18705, 2.6568, 9.12343, 9.172, 1.98505, 8.14777, 255.784

2020-12-13 23:30:00, -55.0315, -53.7586, -3.47158, 7.27059, 4.63612, 0.204175, 6.4262, 4.18293, 3.93193, 33.8672, -53.5459, 55.8665, 56.0082, -3.14105, 1641.75, 4.73291, 0.148989, 2.60047, 398584, 0.0197, 0.233475, 2.58955, 9.19848, 9.2668, 2.2453, 10.5453, 257.018

2020-12-14 00:30:00, -55.165, -53.5862, -5.31263, 11.3905, 7.38775, 0.2391, 7.2815, 4.7294, 4.55656, 33.9324, -43.6015, 54.5719, 54.7271, -3.00669, 1578.19, 5.45164, 0.177203, 2.54727, 459542, 0.019, 0.282275, 2.5311, 9.26262, 9.35888, 2.3999, 15.5629, 258.149

2020-12-14 01:30:00, -55.307, -53.4033, -2.3927, 7.30086, 4.71369, 0.239175, 7.29085, 4.58248, 4.57701, 33.9447, -40.2115, 54.5411, 54.683, -2.1099, 1576.83, 6.13507, 0.205911, 2.50433, 526978, 0.0185, 0.340675, 2.48035, 9.31595, 9.4543, 2.54157, 17.2701, 259.156

2020-12-14 02:30:00, -55.4532, -53.2197, -2.68843, 7.79152, 4.99754, 0.2523, 7.60175, 4.37127, 4.36692, 33.9359, -36.0416, 54.9836, 55.105, -2.23683, 1598.66, 6.71903, 0.236556, 2.47107, 593782, 0.018075, 0.40635, 2.43663, 9.35505, 9.55092, 2.68095, 17.444, 260.08

2020-12-14 08:30:00, -56.3584, -52.0949, -4.22675, 13.9794, 8.65643, 0.2855, 8.3457, 3.24703, 3.24751, 33.8527, -24.8434, 57.4004, 57.445, -2.03287, 1718.92, 7.3646, 0.281712, 2.36735, 657063, 0.0175, 0.786825, 2.23235, 9.30645, 10.0117, 3.69752, 352.859, 265.767

2020-12-14 09:30:00, -56.5093, -51.916, -4.42035, 12.1057, 7.43958, 0.285125, 8.34533, 3.0063, 3.03917, 33.8392, -35.7188, 57.8628, 57.8956, -2.47126, 1742.25, 7.24985, 0.270036, 2.35438, 634245, 0.01735, 0.778075, 2.22168, 9.32555, 10.0221, 3.7152, 353.672, 267.341

2020-12-14 11:30:00, -56.7887, -51.577, -5.7647, 18.6648, 11.1515, 0.2575, 7.7285, 2.11505, 2.12589, 33.7922, -26.9438, 59.9171, 59.9276, -2.15267, 1847.13, 7.38624, 0.266601, 2.33918, 624176, 0.01705, 0.782475, 2.20403, 9.3703, 10.0842, 3.7748, 353.588, 269.736

2020-12-14 12:30:00, -56.9272, -51.3951, -5.07335, 15.3034, 9.12851, 0.253, 7.62125, 2.06458, 2.03775, 33.7844, -28.4224, 60.116, 60.1301, -2.31752, 1857.32, 7.48665, 0.271673, 2.33617, 632936, 0.016975, 0.80005, 2.1944, 9.38697, 10.133, 3.84612, 352.454, 270.539

2020-12-14 13:30:00, -57.0665, -51.2172, -6.3808, 16.0391, 9.58635, 0.2735, 8.09103, 2.127, 2.0983, 33.781, -39.8452, 59.9711, 59.9936, -2.77536, 1849.75, 7.34533, 0.265819, 2.33367, 620289, 0.0169, 0.8017, 2.19088, 9.40328, 10.1558, 3.85848, 352.185, 271.617

2020-12-14 16:30:00, -57.5148, -50.7276, -3.79647, 9.73811, 5.96418, 0.226067, 6.9884, 2.8981, 2.86445, 33.8525, -35.5017, 58.1974, 58.27, -2.6523, 1759.54, 7.23195, 0.261037, 2.32785, 609640, 0.0168, 0.802, 2.18357, 9.42697, 10.1866, 3.89365, 349.741, 274.692

2020-12-14 19:30:00, -57.9798, -50.2091, 2.13495, -5.75054, -3.47512, 0.2006, 6.35475, 2.47455, 2.5215, 33.8138, -33.0978, 58.9907, 59.0347, -2.55337, 1799.64, 6.51204, 0.236305, 2.31688, 539025, 0.0166, 0.6916, 2.20653, 9.45065, 10.0469, 3.58225, 342.963, 279.612

2020-12-15 22:30:00, -60.7005, -45.5776, -12.8432, 29.0649, 15.9331, 0.305475, 8.78983, -0.571525, -0.562405, 33.7677, -39.8628, 66.5179, 66.4904, -3.35881, 2196.42, 9.40121, 0.400907, 1.22805, 502758, 0.014375, 0.727825, 0.980925, 7.40455, 9.95932, 3.13302, 108.003, 291.309

2020-12-16 11:30:00, -60.6994, -45.5773, -12.5211, 29.474, 16.1762, 0.354225, 9.79767, -0.53545, -0.556439, 33.7279, -39.4888, 66.4414, 66.4886, -3.22556, 2191.55, 11.1188, 0.47516, 1.69407, 800067, 0.02735, 1.37735, 0.971225, 6.3007, 10.0867, 4.5757, 141.639, 287.974

2020-12-16 12:30:00, -60.6994, -45.5773, -10.5599, 22.9957, 12.6288, 0.32605, 9.2131, -0.515775, -0.533581, 33.7214, -42.3514, 66.3912, 66.431, -3.48445, 2188.72, 11.262, 0.473846, 1.71443, 811515, 0.0278, 1.40402, 0.9677, 6.28125, 10.0528, 4.64255, 143.091, 287.783

2020-12-16 13:30:00, -60.6994, -45.5773, -10.4814, 21.3192, 11.713, 0.32905, 9.2767, -0.5028, -0.518211, 33.7173, -47.3546, 66.3551, 66.3921, -3.72875, 2186.69, 11.297, 0.475763, 1.735, 821554, 0.02835, 1.42782, 0.9673, 6.259, 9.98302, 4.70005, 144.257, 287.683

2020-12-16 14:30:00, -60.6994, -45.5773, -13.4962, 25.5616, 14.0499, 0.3029, 8.72585, -0.489225, -0.503314, 33.7117, -51.0881, 66.3187, 66.3551, -4.00248, 2184.62, 11.3329, 0.475638, 1.7533, 832080, 0.028825, 1.44775, 0.968325, 6.24125, 9.90573, 4.74825, 145.501, 287.605

2020-12-16 15:30:00, -60.6994, -45.5773, -15.3842, 26.4258, 14.5319, 0.30945, 8.86638, -0.474425, -0.491469, 33.7071, -56.1952, 66.2912, 66.3257, -4.41171, 2183.05, 11.2672, 0.474983, 1.7683, 834344, 0.029125, 1.4616, 0.9723, 6.23475, 9.8264, 4.78895, 147.617, 288.533

2020-12-16 17:30:00, -60.6994, -45.5773, -14.1556, 21.1998, 11.6631, 0.281725, 8.26853, -0.460925, -0.468849, 33.6933, -69.0564, 66.2537, 66.2714, -5.05736, 2180.77, 10.8391, 0.450197, 1.77425, 791851, 0.0286, 1.456, 0.98845, 6.30465, 9.70628, 4.8709, 149.062, 289.756

2020-12-16 18:30:00, -60.6994, -45.5773, -11.8262, 16.6968, 9.1845, 0.242275, 7.3637, -0.4652, -0.466369, 33.6902, -73.0338, 66.2494, 66.266, -5.36402, 2180.49, 10.7255, 0.440039, 1.77027, 773632, 0.02795, 1.44137, 0.998625, 6.37218, 9.7181, 4.89522, 148.1, 291.74

2020-12-16 19:30:00, -60.6994, -45.5773, -7.89423, 10.692, 5.88052, 0.231833, 7.1198, -0.469867, -0.476697, 33.6837, -76.7891, 66.2777, 66.2952, -5.59352, 2181.89, 10.3713, 0.423263, 1.7553, 730958, 0.026825, 1.40415, 1.02145, 6.47523, 9.7001, 4.90005, 147.806, 295.404

2020-12-16 21:30:00, -60.6993, -45.5773, -9.2479, 10.9939, 6.05799, 0.239175, 7.29102, -0.411325, -0.431829, 33.6878, -90.835, 66.1456, 66.1768, -6.36281, 2174.85, 9.33895, 0.357291, 1.6658, 586635, 0.023, 1.22313, 1.10272, 6.81102, 9.48133, 4.8185, 147.565, 296.844

2020-12-16 23:30:00, -60.6989, -45.5771, -8.3619, 10.7623, 5.92736, 0.142475, 4.69928, -0.42715, -0.432097, 33.6899, -80.8607, 66.1663, 66.1767, -5.87825, 2176, 8.53074, 0.315069, 1.55882, 482511, 0.01965, 1.03005, 1.14803, 7.12365, 9.3429, 4.6063, 145.407, 294.606

2020-12-17 00:30:00, -60.699, -45.5772, -5.85197, 7.46831, 4.11316, 0.129025, 4.30518, -0.427275, -0.432669, 33.6896, -80.9146, 66.1704, 66.1784, -5.92863, 2176.21, 7.85614, 0.286287, 1.49717, 420190, 0.01815, 0.891325, 1.18538, 7.27163, 9.09863, 4.3503, 145.315, 280.434

2020-12-17 03:30:00, -60.699, -45.5774, -6.27862, 7.04494, 3.88818, 0.131375, 4.3599, -0.361875, -0.377699, 33.6789, -94.8562, 66.0016, 66.0391, -6.72869, 2166.93, 6.47002, 0.228943, 1.30163, 293441, 0.01425, 0.5439, 1.17625, 7.65618, 8.68567, 3.33723, 142.552, 237.675

2020-12-17 04:30:00, -60.6991, -45.5774, -5.53943, 6.39699, 3.52882, 0.121575, 4.0455, -0.377325, -0.393916, 33.6792, -92.1719, 66.0357, 66.0811, -6.54048, 2168.77, 5.94511, 0.210426, 1.2431, 256444, 0.01315, 0.457375, 1.15132, 7.78672, 8.63145, 3.00682, 143.25, 230.791

2020-12-17 07:30:00, -60.6989, -45.5769, -2.3995, 2.82839, 1.55856, 0.122525, 4.07135, -0.410725, -0.437086, 33.6838, -89.8557, 66.1421, 66.1919, -6.41666, 2174.58, 4.48951, 0.150412, 1.10262, 162992, 0.01005, 0.251, 1.07215, 8.39805, 8.77535, 2.25925, 143.958, 249.58

2020-12-17 08:30:00, -60.6992, -45.5768, -1.27767, 1.45571, 0.802659, 0.09935, 3.285, -0.39145, -0.404507, 33.6833, -92.8632, 66.0708, 66.1072, -6.63223, 2170.73, 4.27425, 0.139621, 1.07263, 148642, 0.0092, 0.2159, 1.04952, 8.64262, 8.95355, 2.13388, 137.448, 258.253

2020-12-17 11:30:00, -60.705, -45.5975, -3.1313, 3.31034, 1.82522, 0.0736, 2.3937, -0.392375, -0.391926, 33.6313, -102.845, 66.1165, 66.0924, -7.15069, 2172.23, 3.45304, 0.112886, 1.03048, 112379, 0.0075, 0.1377, 1.02035, 9.3879, 9.55222, 1.76865, 100.279, 270.473

2020-12-17 12:30:00, -60.705, -45.5975, -2.03037, 2.15829, 1.19194, 0.06635, 2.13902, -0.34215, -0.327784, 33.6349, -100.674, 65.9662, 65.9247, -7.09803, 2164.2, 2.80133, 0.0912623, 1.02318, 89536.4, 0.007075, 0.08455, 1.01895, 9.64265, 9.71555, 1.4224, 88.8011, 273.106

2020-12-17 13:30:00, -60.705, -45.5974, -3.7023, 3.88275, 2.14463, 0.0664, 2.14365, -0.337325, -0.320122, 33.6351, -103.6, 65.9505, 65.9048, -7.19308, 2163.36, 2.26644, 0.072688, 1.01875, 72027.9, 0.006675, 0.04415, 1.01763, 9.87528, 9.89672, 1.1211, 73.6485, 275.21

2020-12-17 14:30:00, -60.705, -45.5975, -5.0515, 5.19898, 2.87311, 0.0692, 2.23937, -0.32155, -0.314162, 33.6301, -105.984, 65.9211, 65.8911, -7.32704, 2161.69, 2.10181, 0.065709, 1.01662, 66647.6, 0.006425, 0.028025, 1.0162, 10.0736, 10.0823, 1.0322, 61.2045, 276.672

2020-12-17 15:30:00, -60.705, -45.5975, -3.30093, 3.24704, 1.79609, 0.063, 2.02557, -0.292475, -0.281063, 33.6347, -111.094, 65.8494, 65.8039, -7.65952, 2157.92, 2.14526, 0.066476, 1.0156, 67648.9, 0.0062, 0.02315, 1.01525, 10.2362, 10.2423, 1.0429, 53.6641, 277.611

2020-12-17 16:30:00, -60.705, -45.5974, -2.48473, 2.34561, 1.29946, 0.051375, 1.62085, -0.244875, -0.216425, 33.6308, -117.687, 65.7208, 65.6385, -7.96824, 2150.93, 2.26764, 0.06935, 1.01445, 71072.5, 0.00605, 0.0228, 1.01415, 10.366, 10.3718, 1.08805, 51.9426, 278.218

2020-12-17 17:30:00, -60.705, -45.5974, -2.46127, 2.23785, 1.242, 0.04545, 1.41185, -0.188775, -0.160533, 33.624, -122.084, 65.6091, 65.4971, -8.26199, 2144.8, 2.53611, 0.0764373, 1.01258, 78827.7, 0.005925, 0.027875, 1.01217, 10.4661, 10.4746, 1.16047, 55.0426, 278.607

2020-12-17 18:30:00, -60.7051, -45.5973, -1.7087, 1.45781, 0.810478, 0.0441, 1.36978, -0.135325, -0.0730747, 33.6377, -133.315, 65.3852, 65.2685, -8.77873, 2133.04, 2.87106, 0.0867123, 1.00972, 89115.1, 0.005825, 0.041375, 1.00877, 10.5344, 10.5534, 1.16735, 58.6511, 278.872

2020-12-17 21:30:00, -60.7007, -45.5768, -0.179533, 0.13164, 0.0730098, 0.058975, 1.8931, -0.21005, -0.195936, 33.6456, -156.431, 65.6338, 65.5807, -10.2486, 2146.53, 3.01398, 0.0956517, 0.984525, 91477.2, 0.00565, 0.076825, 0.98125, 10.5677, 10.6333, 1.37107, 50.2964, 280.539

2020-12-17 23:30:00, -60.7001, -45.5768, -1.2189, 1.08798, 0.600734, 0.05045, 1.6054, -0.34805, -0.312365, 33.682, -124.894, 65.9876, 65.8686, -8.45763, 2166.23, 3.60571, 0.108915, 0.962975, 106737, 0.0055, 0.091075, 0.958475, 10.5571, 10.6518, 1.4259, 49.291, 280.908

2020-12-18 00:30:00, -60.7001, -45.5769, -2.0268, 1.9273, 1.06364, 0.03365, 1.04407, -0.363475, -0.170795, 33.6835, -127.194, 65.9167, 65.5032, -7.9297, 2162.44, 3.98123, 0.121686, 0.95195, 117136, 0.0055, 0.114875, 0.9447, 10.4994, 10.6506, 1.5108, 51.0092, 281.058

2020-12-18 01:30:00, -60.7001, -45.5765, -4.4442, 4.31192, 2.37666, 0.045875, 1.46928, -0.402775, -0.293427, 33.6908, -104.676, 66.2274, 65.8166, -7.80626, 2179.31, 4.28003, 0.132838, 0.9407, 125815, 0.005575, 0.1401, 0.929725, 10.4133, 10.6387, 1.60635, 50.6052, 281.234

2020-12-18 02:30:00, -60.7005, -45.5757, 1.5937, -1.30159, -0.720327, 0.090575, 2.99925, -0.27725, -0.258585, 33.6432, -143.729, 65.7109, 65.7429, -9.20737, 2150.63, 4.31856, 0.137527, 0.928425, 126881, 0.0056, 0.1578, 0.914375, 10.3232, 10.6127, 1.67175, 48.9052, 281.421

2020-12-18 03:30:00, -60.7006, -45.5755, 0.437, -0.356811, -0.197339, 0.081825, 2.6873, -0.297175, -0.328938, 33.6519, -131.194, 65.8977, 65.9219, -9.23505, 2160.83, 4.23525, 0.135168, 0.914625, 123114, 0.0056, 0.166425, 0.898775, 10.2511, 10.5806, 1.7257, 48.8838, 281.652

2020-12-18 04:30:00, -60.7008, -45.5757, -0.059175, 0.0420475, 0.0233995, 0.06885, 2.20975, -0.10485, -0.145215, 33.6256, -157.883, 65.4214, 65.4573, -10.548, 2134.76, 4.29105, 0.1361, 0.900175, 122669, 0.005575, 0.172525, 0.882775, 10.1874, 10.5499, 1.77265, 48.5995, 281.864

2020-12-18 05:30:00, -60.7007, -45.5755, 1.35653, -0.90412, -0.503637, 0.090725, 2.98405, -0.07445, -0.0876469, 33.6424, -169.41, 65.2316, 65.3041, -11.2161, 2124.89, 4.3953, 0.139738, 0.88585, 123795, 0.0055, 0.17975, 0.86665, 10.1162, 10.5184, 1.81157, 49.0737, 282.155

2020-12-18 06:30:00, -60.7006, -45.5753, 0.324925, -0.215935, -0.119787, 0.0748, 2.42487, -0.203575, -0.195162, 33.6645, -171.144, 65.5545, 65.5723, -11.2954, 2142.62, 4.4731, 0.142415, 0.872175, 124291, 0.0055, 0.188975, 0.850625, 10.0392, 10.4921, 1.85997, 52.0375, 282.495

2020-12-18 07:30:00, -60.7006, -45.5755, 0.0452, -0.0361398, -0.0199606, 0.035125, 1.00335, -0.339175, -0.213417, 33.6853, -155.303, 65.9144, 65.6121, -9.43207, 2162.35, 4.63187, 0.147165, 0.86005, 127340, 0.005575, 0.2005, 0.83535, 9.95003, 10.4692, 1.90535, 55.0914, 282.775

2020-12-18 08:30:00, -60.7007, -45.5757, -0.2, 0.171884, 0.0948327, 0.035275, 1.05165, -0.372375, -0.291873, 33.6793, -135.287, 66.0129, 65.8165, -8.7858, 2167.54, 4.91853, 0.156795, 0.850575, 134760, 0.00565, 0.21805, 0.82105, 9.82868, 10.4482, 1.9591, 57.2782, 282.985

2020-12-18 09:30:00, -60.7009, -45.5759, -0.397167, 0.365392, 0.20157, 0.0436, 1.34085, -0.3763, -0.3349, 33.6845, -121.505, 66.0455, 65.9261, -8.21134, 2169.39, 5.30184, 0.171173, 0.845125, 146222, 0.0058, 0.2447, 0.8076, 9.6589, 10.4353, 2.03978, 60.0791, 283.197

2020-12-18 16:30:00, -60.6654, -45.6253, -7.2561, 8.27302, 4.55728, 0.0853, 2.82422, -0.420975, -0.407451, 33.7491, -91.5908, 66.139, 66.0922, -6.63533, 2175.63, 4.87027, 0.161592, 0.831775, 132397, 0.0064, 0.27575, 0.782225, 9.12728, 10.1033, 2.35498, 52.6578, 288.676

2020-12-18 21:30:00, -60.701, -45.5754, -1.62963, 1.05116, 0.584354, 0.0701, 2.29232, -0.1377, -0.132042, 33.6735, -175.259, 65.4213, 65.4072, -11.6198, 2135.63, 4.90933, 0.154643, 0.869475, 137495, 0.005525, 0.222975, 0.83935, 10.0431, 10.6636, 2.11407, 76.1655, 283.381

2020-12-18 22:30:00, -60.701, -45.5753, -1.54413, 1.00514, 0.556757, 0.0493, 1.56945, -0.24985, -0.164096, 33.684, -183.244, 65.6012, 65.4858, -11.5375, 2145.49, 5.32678, 0.173926, 0.890975, 155967, 0.005725, 0.258, 0.851725, 9.99065, 10.7762, 2.17298, 78.8511, 282.639

2020-12-19 01:30:00, -60.7005, -45.5765, -1.2997, 2.22926, 1.21607, 0.050775, 1.5976, -0.723725, -0.662609, 33.8274, -67.5059, 66.8243, 66.7332, -4.44853, 2214.12, 5.74736, 0.19548, 0.952775, 186045, 0.0063, 0.328325, 0.892775, 9.82662, 10.9217, 2.37672, 73.879, 280.445

2020-12-19 02:30:00, -60.7005, -45.5764, -0.803, 1.81758, 0.988834, 0.026325, 0.7043, -0.807025, -0.596799, 33.8478, -47.3797, 67.1024, 66.5529, -3.38333, 2229.57, 5.79293, 0.198757, 0.967125, 191487, 0.00645, 0.33805, 0.90435, 9.80942, 10.9312, 2.41925, 68.7354, 279.686

2020-12-19 03:30:00, -60.7005, -45.5769, 1.7187, -5.04553, -2.744, 0.029875, 0.837225, -0.817975, -0.654838, 33.8685, -32.7485, 67.1166, 66.6984, -2.60928, 2230.73, 5.58147, 0.193195, 0.97595, 185314, 0.006475, 0.333825, 0.915175, 9.83843, 10.9112, 2.44223, 65.1136, 279.205

2020-12-19 04:30:00, -60.7005, -45.5762, 1.1086, -2.86787, -1.56001, 0.038975, 1.16015, -0.811375, -0.764408, 33.8731, -27.8028, 67.1705, 66.9863, -2.96358, 2233.75, 5.1994, 0.176939, 0.9771, 170003, 0.00635, 0.31325, 0.923725, 9.93185, 10.8743, 2.43838, 64.4203, 279.064

2020-12-19 08:30:00, -60.701, -45.576, 0.9439, -1.784, -0.971244, 0.042475, 1.28335, -0.78545, -0.705734, 33.8186, -59.3806, 67.0302, 66.8501, -4.0475, 2225.1, 4.82909, 0.157851, 0.955875, 150217, 0.0059, 0.252875, 0.92075, 10.1812, 10.8373, 2.27545, 79.9441, 278.909

2020-12-19 09:30:00, -60.701, -45.5758, -1.2092, 2.0772, 1.13267, 0.04025, 1.22285, -0.736, -0.662582, 33.789, -44.035, 66.9749, 66.7464, -4.45045, 2221.55, 4.6814, 0.154042, 0.9474, 144225, 0.005875, 0.24175, 0.9148, 10.1652, 10.7817, 2.20657, 83.2202, 278.82

2020-12-19 11:30:00, -60.7005, -45.5772, -3.2701, 7.6339, 4.15359, 0.032625, 0.997525, -0.8037, -0.686624, 33.8597, -37.1297, 67.1578, 66.7852, -3.2834, 2232.8, 4.64932, 0.149354, 0.9253, 139142, 0.0058, 0.232975, 0.894225, 10.1036, 10.6957, 2.17938, 83.2324, 278.254

2020-12-19 12:30:00, -60.7005, -45.5772, -4.1124, 7.82278, 4.2649, 0.03195, 0.9745, -0.74165, -0.584028, 33.8389, -51.8402, 66.9162, 66.5225, -4.0164, 2219.31, 5.01474, 0.161427, 0.916025, 149987, 0.0059, 0.2514, 0.8794, 9.9826, 10.6716, 2.2185, 82.2757, 277.918

2020-12-19 13:30:00, -60.7005, -45.5772, 1.59315, -2.90623, -1.58386, 0.022925, 0.677975, -0.7531, -0.513712, 33.8451, -55.7626, 67.0243, 66.3361, -4.19465, 2225.29, 5.39158, 0.178351, 0.90965, 163121, 0.0061, 0.281575, 0.86325, 9.7817, 10.6436, 2.28645, 82.5648, 277.677

2020-12-20 06:30:00, -61.3922, -51.1073, -4.20958, 9.87257, 5.42622, 0.295875, 8.58373, -0.49055, -0.468561, 34.3382, -36.8596, 66.0219, 66.048, -3.22835, 2180.28, 7.62172, 0.28655, 1.17893, 345392, 0.01525, 0.641675, 0.98525, 7.06185, 8.64663, 3.2896, 331.073, 278.333

2020-12-20 13:30:00, -61.7055, -53.7562, 16.9587, 62.5575, 34.5947, 0.5709, 13.8268, -0.300425, -0.300083, 34.3289, 48.5693, 65.5802, 65.6151, 2.04144, 2156.23, 10.9486, 0.498882, 2.07418, 992493, 0.036325, 1.73612, 1.1321, 6.04887, 8.4176, 5.0865, 17.9908, 281.534

2020-12-20 14:30:00, -61.7436, -54.1269, 10.2792, 31.1506, 17.2075, 0.427225, 11.2134, -0.334675, -0.34583, 34.3342, 53.8398, 65.7279, 65.7313, 2.49, 2164.31, 9.22802, 0.389313, 1.9963, 741122, 0.032375, 1.53215, 1.26607, 6.28988, 8.12567, 5.0758, 24.8823, 293.835

2020-12-20 23:30:00, -62.2378, -57.1565, -7.0588, 27.091, 15.6679, 0.4833, 12.2849, 1.09465, 1.06091, 34.1481, -16.2066, 62.2757, 62.2946, -1.87907, 1976.48, 10.2592, 0.420236, 1.44137, 589772, 0.029975, 1.28025, 0.65665, 5.5483, 8.58465, 4.7553, 354.959, 304.599

2020-12-21 00:30:00, -62.3037, -57.4794, -9.18243, 30.9069, 17.9103, 0.351425, 9.71555, 1.15673, 1.15918, 34.1481, -21.284, 62.0575, 62.06, -2.13592, 1964.95, 9.23649, 0.367162, 1.3622, 488695, 0.027675, 1.14397, 0.7307, 5.61463, 8.14982, 4.58315, 351.903, 17.9731

2020-12-21 01:30:00, -62.3754, -57.8006, -2.84613, 9.45514, 5.47153, 0.30745, 8.82282, 1.11302, 1.13555, 34.1555, -26.6651, 62.1495, 62.1139, -2.16675, 1969.94, 9.12057, 0.344787, 1.33955, 484424, 0.0267, 1.05495, 0.821175, 5.6701, 7.92055, 4.33365, 341.365, 13.9846

2021-01-01 02:30:00, -67.752, -67.2702, -3.2668, 1.8046, 1.07509, 0.038475, 1.06478, 2.02493, 1.96445, 33.2718, -215.643, 60.2548, 60.4573, -12.6474, 1856.25, NaN, NaN, NaN, NaN, NaN, NaN, NaN, NaN, NaN, NaN, NaN, NaN

2021-01-01 03:30:00, -67.6954, -67.1882, -0.9501, 0.464166, 0.269701, 0.047625, 1.43162, 1.24045, 1.2962, 33.2389, -242.737, 61.8891, 62.0268, -14.6143, 1940.74, NaN, NaN, NaN, NaN, NaN, NaN, NaN, NaN, NaN, NaN, NaN, NaN

2021-01-01 08:30:00, -67.8302, -67.5327, 1.2415, -1.02979, -0.594176, 0.0351, 1.08185, 1.02135, 1.01064, 33.32, -146.948, 62.7758, 62.684, -8.72234, 1988.67, 1.80737, NaN, 0.023, 1298, 0.0005, 0.0145, 0.0179, 5.309, 6.8398, 3.0055, 55.7617, 247.929

2021-01-01 10:30:00, -67.8066, -67.399, -9.6341, 5.86386, 3.39118, 0.1934, 6.05455, 1.09332, 1.02804, 33.1903, -185.241, 62.6592, 62.6843, -11.8622, 1980.32, NaN, NaN, NaN, NaN, NaN, NaN, NaN, NaN, NaN, NaN, NaN, NaN

2021-01-01 13:30:00, -67.8023, -67.3016, -24.227, 10.231, 5.9643, 0.211775, 6.61402, 1.34355, 1.33539, 33.095, -283.316, 61.9251, 61.98, -16.9153, 1940.21, NaN, NaN, NaN, NaN, NaN, NaN, NaN, NaN, NaN, NaN, NaN, NaN

2021-01-01 14:30:00, -67.8021, -67.3013, -48.7164, 20.4421, 11.8575, 0.2106, 6.57925, 1.18697, 1.23227, 33.1033, -284.808, 62.1857, 62.2226, -17.0788, 1953.99, NaN, NaN, NaN, NaN, NaN, NaN, NaN, NaN, NaN, NaN, NaN, NaN

2021-01-01 15:30:00, -67.8021, -67.3013, -61.3671, 26.5813, 15.3478, 0.259725, 7.74565, 1.04317, 1.02002, 33.1552, -276.453, 62.7015, 62.7151, -16.6713, 1981.94, NaN, NaN, NaN, NaN, NaN, NaN, NaN, NaN, NaN, NaN, NaN, NaN

2021-01-01 16:30:00, -67.8021, -67.3013, -41.1241, 17.7248, 10.1912, 0.328425, 9.28032, 0.91175, 0.944274, 33.1623, -276.22, 62.8973, 62.8959, -16.7929, 1992.36, NaN, NaN, NaN, NaN, NaN, NaN, NaN, NaN, NaN, NaN, NaN, NaN

2021-01-01 17:30:00, -67.8021, -67.3015, -60.1174, 28.3007, 16.2736, 0.32125, 9.11855, 0.914725, 0.904158, 33.2061, -264.512, 62.9942, 62.9788, -15.4024, 1998.22, NaN, NaN, NaN, NaN, NaN, NaN, NaN, NaN, NaN, NaN, NaN, NaN

2021-01-01 18:30:00, -67.8019, -67.3018, -64.3281, 31.0978, 17.9081, 0.265475, 7.91057, 0.9603, 0.951418, 33.2455, -246.936, 62.8359, 62.8514, -14.9686, 1990.56, NaN, NaN, NaN, NaN, NaN, NaN, NaN, NaN, NaN, NaN, NaN, NaN

2021-01-02 02:30:00, -68.014, -67.6767, -6.71017, 6.54751, 3.68973, 0.1514, 4.96718, 0.2854, 0.289841, 33.4389, -117.606, 64.4127, 64.4172, -7.5757, 2077.46, NaN, NaN, NaN, NaN, NaN, NaN, NaN, NaN, NaN, NaN, NaN, NaN

2021-01-02 03:30:00, -67.962, -67.6789, -6.11512, 6.33263, 3.5714, 0.09065, 2.98293, 0.3095, 0.333458, 33.4698, -108.645, 64.3394, 64.2976, -7.13236, 2074.11, 2.83081, NaN, 0.094, 8298.7, 0.0154, 0.0589, 0.0732, 1.9778, 2.4333, 1.2757, 62.9668, 36.2636

2021-01-02 04:30:00, -67.9084, -67.6619, -3.64935, 3.6319, 2.05648, 0.0345, 1.06917, 0.434, 0.515101, 33.4675, -109.486, 64.1175, 63.8464, -7.40202, 2062.27, 2.77804, 0.087436, 0.08795, 7606.92, 0.014325, 0.0554, 0.0684, 1.9837, 2.46742, 1.2457, 64.2269, 37.2446

2021-01-02 06:30:00, -67.8955, -67.6493, -19.8089, 18.4002, 10.4233, 0.075725, 2.4861, 0.4476, 0.489686, 33.4625, -121.952, 63.9557, 63.9111, -7.91114, 2053.58, 2.16522, 0.070476, 0.0645, 4389.94, 0.00885, 0.03085, 0.05585, 2.16538, 2.49575, 1.15848, 64.5844, 47.3568

2021-01-02 07:30:00, -67.9522, -67.6688, 0.4745, -0.45807, -0.258847, 0.069825, 2.28465, 0.3709, 0.412002, 33.4685, -116.473, 64.1183, 64.1021, -7.62779, 2062.33, 1.91235, 0.060235, 0.0588, 3527, 0.0074, 0.0205, 0.0544, 2.2593, 2.4611, 1.3443, 63.8382, 50.8184

2021-01-02 08:30:00, -68.0097, -67.6691, 1.87165, -1.7321, -0.976421, 0.04675, 1.48197, 0.295775, 0.318388, 33.4483, -118.228, 64.4434, 64.3425, -7.99234, 2079.27, NaN, NaN, NaN, NaN, NaN, NaN, NaN, NaN, NaN, NaN, NaN, NaN

2021-01-02 11:30:00, -68.1423, -67.5815, 6.0368, -4.07528, -2.22954, 0.014525, 0.4143, -0.634, -0.72051, 32.3538, -167.623, 68.3587, 67.4028, -11.4785, 2268.92, NaN, NaN, NaN, NaN, NaN, NaN, NaN, NaN, NaN, NaN, NaN, NaN

2021-01-03 12:30:00, -67.7154, -68.3962, 1.11122, -2.38725, -1.31993, 0.027075, 0.8167, -0.30605, -0.125854, 33.456, -49.1544, 65.9258, 65.4655, -3.50764, 2158.73, NaN, NaN, NaN, NaN, NaN, NaN, NaN, NaN, NaN, NaN, NaN, NaN

2021-01-03 13:30:00, -67.7154, -68.3962, 1.8228, -3.75365, -2.07433, 0.02565, 0.77265, -0.322325, -0.102669, 33.4508, -52.1485, 65.981, 65.4078, -3.66182, 2161.59, NaN, NaN, NaN, NaN, NaN, NaN, NaN, NaN, NaN, NaN, NaN, NaN

2021-01-03 14:30:00, -67.7154, -68.3962, -1.2763, 2.39145, 1.32256, 0.043525, 1.3693, -0.2987, -0.244207, 33.4442, -56.6566, 65.942, 65.7741, -4.02257, 2159.37, NaN, NaN, NaN, NaN, NaN, NaN, NaN, NaN, NaN, NaN, NaN, NaN

2021-01-03 15:30:00, -67.7154, -68.3962, -0.457467, 0.792064, 0.43882, 0.053025, 1.69568, -0.2435, -0.193064, 33.4437, -61.9064, 65.7346, 65.6424, -4.34123, 2148.23, NaN, NaN, NaN, NaN, NaN, NaN, NaN, NaN, NaN, NaN, NaN, NaN

2021-01-08 09:30:00, -64.8283, -63.5347, 3.95437, -15.0016, -8.70996, 0.17775, 5.66138, 1.2166, 1.20581, 33.6497, -20.0552, 62.0746, 62.1095, -1.89141, 1957.4, NaN, NaN, NaN, NaN, NaN, NaN, NaN, NaN, NaN, NaN, NaN, NaN

2021-01-08 21:30:00, -64.8274, -63.5344, -2.5152, 8.20914, 4.78655, 0.075125, 2.4394, 1.34967, 1.28368, 33.6495, -23.6488, 61.7811, 61.9245, -2.18993, 1941.98, NaN, NaN, NaN, NaN, NaN, NaN, NaN, NaN, NaN, NaN, NaN, NaN

2021-01-09 00:30:00, -64.8276, -63.5333, -0.4639, 1.73612, 1.01202, 0.0326, 0.8555, 1.3415, 1.32149, 33.604, -21.5215, 61.7794, 61.8495, -1.90925, 1941.13, NaN, NaN, NaN, NaN, NaN, NaN, NaN, NaN, NaN, NaN, NaN, NaN

2021-01-09 06:30:00, -64.8277, -63.5348, 4.43765, -12.6675, -7.35279, 0.044875, 1.32527, 1.2081, 1.21077, 33.6123, -30.0309, 62.0834, 62.1097, -2.51339, 1957.23, NaN, NaN, NaN, NaN, NaN, NaN, NaN, NaN, NaN, NaN, NaN, NaN

2021-01-09 07:30:00, -64.828, -63.5349, 1.27367, -4.05124, -2.34863, 0.096575, 3.16375, 1.1697, 1.128, 33.6092, -28.0484, 62.2101, 62.3083, -2.25964, 1963.84, NaN, NaN, NaN, NaN, NaN, NaN, NaN, NaN, NaN, NaN, NaN, NaN

2021-01-11 11:30:00, -64.6456, -62.1211, -3.76107, 13.298, 7.90632, 0.1497, 4.65387, 1.96125, 2.0332, 33.1335, -20.099, 60.3643, 60.3433, -1.9774, 1859.68, 3.38428, 0.108104, 0.1305, 14059.9, 0.0026, 0.0761, 0.106, 5.6401, 7.8589, 1.36147, 180.06, 349.791
